# SNPs in Genes Functional in Starch-Sugar Interconversion Associate with Natural Variation of Tuber Starch and Sugar Content of Potato (*Solanum tuberosum* L.)

**DOI:** 10.1534/g3.114.012377

**Published:** 2014-07-31

**Authors:** Lena Schreiber, Anna Camila Nader-Nieto, Elske Maria Schönhals, Birgit Walkemeier, Christiane Gebhardt

**Affiliations:** Max Planck Institute for Plant Breeding Research, Department of Plant Breeding and Genetics, 50829 Cologne, Germany

**Keywords:** complex trait, starch metabolism, association mapping, marker-assisted selection, potato (*Solanum tuberosum* L.)

## Abstract

Starch accumulation and breakdown are vital processes in plant storage organs such as seeds, roots, and tubers. In tubers of potato (*Solanum tuberosum* L.) a small fraction of starch is converted into the reducing sugars glucose and fructose. Reducing sugars accumulate in response to cold temperatures. Even small quantities of reducing sugars affect negatively the quality of processed products such as chips and French fries. Tuber starch and sugar content are inversely correlated complex traits that are controlled by multiple genetic and environmental factors. Based on *in silico* annotation of the potato genome sequence, 123 loci are involved in starch-sugar interconversion, approximately half of which have been previously cloned and characterized. By means of candidate gene association mapping, we identified single-nucleotide polymorphisms (SNPs) in eight genes known to have key functions in starch-sugar interconversion, which were diagnostic for increased tuber starch and/or decreased sugar content and *vice versa*. Most positive or negative effects of SNPs on tuber-reducing sugar content were reproducible in two different collections of potato cultivars. The diagnostic SNP markers are useful for breeding applications. An allele of the plastidic starch phosphorylase *PHO1a* associated with increased tuber starch content was cloned as full-length cDNA and characterized. The *PHO1a-H_A_* allele has several amino acid changes, one of which is unique among all known starch/glycogen phosphorylases. This mutation might cause reduced enzyme activity due to impaired formation of the active dimers, thereby limiting starch breakdown.

Starch biosynthesis and breakdown in photosynthetic tissues and storage organs like seeds, roots, and tubers are vital plant processes that have been studied extensively at the biochemical and molecular level (Geigenberger 2011; Tetlow *et al.* 2004; Zeeman *et al.* 2010). The starch polymers amylose and amylopectin are in a dynamic equilibrium with their sugar building blocks, which varies between different organs and in response to developmental or environmental signals. For example, starch accumulates transiently in immature tomato fruits and is completely degraded into glucose and fructose in ripe tomato fruits (Luengwilai and Beckles 2009; Schaffer and Petreikov 1997). Tomato (*Solanum lycopersicum*) and potato (*Solanum tuberosum*) are closely related species with highly syntenic genomes and high gene sequence similarity (The Tomato Genome Consortium 2012). Despite this high similarity at molecular level, potato plants do not form fleshy fruits like tomatoes whereas tomato plants do not form tubers. During maturation, potato tubers accumulate nearly 100% of their carbohydrate reserve as starch and only a small amount in form of the soluble sugars sucrose, glucose, and fructose. During tuber dormancy, the amount of sugars increases at the expense of starch in response to storage at low temperatures (Isherwood 1973). This “cold-induced sweetening” phenomenon is observed in potato and other plants (Müller-Thurgau 1882). The soluble sugars function as cryoprotectants of the cells. The temperature-dependent conversion of starch into sugars is reversible (Isherwood 1973). As a consequence of starch-sugar interconversion in growing as well as dormant tubers, tuber starch and sugar content are inversely correlated (Li *et al.* 2013).

Starch and sugar content of plant storage organs are controlled, besides environmental factors, by multiple genetic factors and therefore show genotype-dependent, quantitative variation. They are complex traits of high agronomic importance. Whereas high sugar content is a positive quality attribute of tomato and other fruits, the opposite is the case for sugars in potato tubers. Even a low content of the reducing sugars glucose and fructose has unwanted effects on the quality of processed tuber products such as chips and French fries. Reducing sugars undergo with amino acids at high temperatures a nonenzymatic Maillard reaction, which results in dark brown−colored products and potentially harmful byproducts such as acrylamide (Dale and Bradshaw 2003; Medeiros Vinci *et al.* 2012). Tuber starch content has to have an optimal range for table and processing potatoes, and starch yield (starch per area unit) is a decisive criterion for the production of potato starch for industrial uses. These quality traits are therefore important in potato breeding programs aiming at the selection of improved varieties for direct consumption, processing and industrial use.

Genes encoding enzymes and transport proteins functional in starch-sugar interconversion (Figure 1) have been cloned and characterized from model and crop plants including potato and tomato. Their function has been elucidated by comparing wild-type plants with loss-of-function mutants and transgenic plants silenced for or overexpressing a particular gene (Fernie *et al.* 2002; Zeeman *et al.* 2010). In contrast, very little is known about the genes and their allelic variants that control the natural variation of starch-sugar interconversion. Genetic evidence and plausibility suggest that genes functional in starch-sugar interconversion underlay, among others, quantitative trait loci (QTL) for starch and sugar related traits in plant storage organs. In tomato, a single-nucleotide polymorphism (SNP) in an invertase gene changed the enzymatic activity of the encoded protein, which was responsible for a sugar yield QTL (Fridman *et al.* 2004). Candidate gene association mapping showed association of SNPs in starch synthases (*SS*) with amylose content of rice grains (Tian *et al.* 2009). SNPs in sucrose synthase (*Sh1*, *SUS*), starch branching enzyme (*ae1*, *SBE*), ADP-glucose pyrophosphorylase (large subunit) (*sh2*, *AGPaseS*), alpha-amylase (*AMY*), and beta-amylase (*BMY*) associated with starch properties of maize kernels (Cook *et al.* 2012; Thévenot *et al.* 2005; Wilson *et al.* 2004). In potato, single-strand conformation polymorphism (SSCP) markers in invertases (*INV*), L-type starch phosphorylases (*PHO1*), soluble starch synthase (*Sss*), and *AGPaseS* were associated with chip or processing quality (corresponding to reducing sugar content) and tuber starch content, in some cases also with tuber yield and starch yield (Li *et al.* 2005, 2008, 2013).

**Figure 1:**
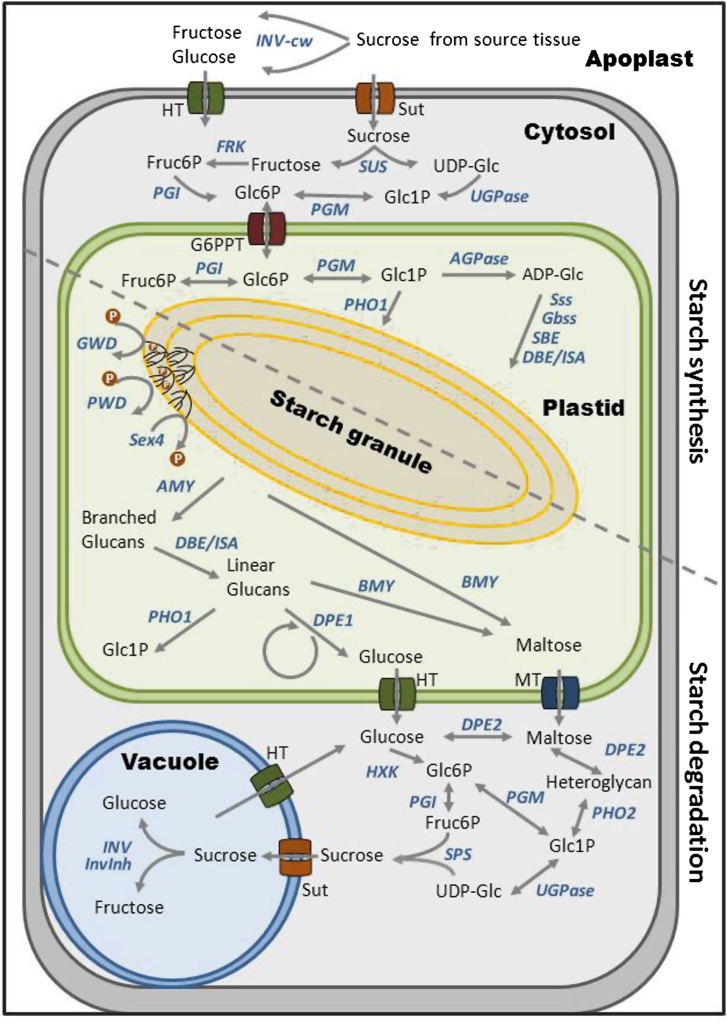
Metabolic scheme for starch-sugar interconversion in potato tubers, adapted and modified from Zeeman *et al.* (2004, 2007, 2010). AGPase, ADP-glucose pyrophosphorylase (EC 2.7.7.27); AMY, alpha-amylase (EC 3.2.1.1); BMY, beta-amylase (EC 3.2.1.2); DBE/ISA, debranching enzyme/isoamylase (EC 3.2.1.68); DPE1 and DPE2, disproportionating enzyme 1 and 2 (EC 2.4.1.25); FRK, fructokinase (EC 2.7.1.4); G6PPT, glucose-6-phosphate/phosphate translocator; GWD, glucan water dikinase (EC 2.7.9.4); HT, hexose transporter; HXK, hexokinase (EC 2.7.1.1); INV, invertase, INV-cw, cell wall−bound invertase (EC 3.2.1.26); InvInh, invertase inhibitor; MT, maltose transporter; PGI, glucose-6-phosphate isomerase (EC 5.3.1.9); PGM, phosphoglucomutase (EC 5.4.2.2); PHO1, L-type starch phosphorylase (EC 2.4.1.1); PHO2, H-type starch phosphorylase (EC 2.4.1.1); PWD, phosphoglucan water dikinase (EC 2.7.9.5); SBE, starch branching enzyme (EC 2.4.1.18); SEX4, phosphoglucan phosphatase 4 (EC 23.1.3.-); SPS, sucrose phosphate synthase (EC 2.4.1.14); Sss, soluble starch synthase, Gbss, granule bound starch synthase (EC 2.4.1.21); SUS, sucrose synthase (EC 2.4.1.13); Sut, sucrose transporter; UGPase, UDP-glucose pyrophosphorylase (EC 2.7.7.9); ADP-Glc, ADP-glucose; Fruc6P, fructose 6-phosphate; Glc6P, glucose 6-phosphate; Glc1P, glucose 1-phosphate; UDP-Glc, UDP-glucose.

So far, 18 loci encoding genes functional in starch-sugar interconversion have been probed for association of natural DNA variation with tuber starch content and/or processing quality (Baldwin *et al.* 2011; Draffehn *et al.* 2010; Fischer *et al.* 2013; Kawchuk *et al.* 2008; Li *et al.* 2005, 2008, 2013). The metabolic scheme shown in Figure 1, however, suggests that this number does not include all important steps in starch biosynthesis and degradation. Furthermore, natural alleles of only few genes functional in starch-sugar interconversion have been characterized at the molecular level (Datir *et al.* 2012; Draffehn *et al.* 2010, 2012; Sowokinos *et al.* 1997). DNA variants associated with tuber quality traits were initially discovered based on the analysis of SSCPs (Dong and Zhu 2005), which were highly suitable for *de novo* discovery of DNA variation in specific loci but not for cost- and labor-effective high-throughput screenings in breeding programs (Li *et al.* 2005, 2008, 2013; Urbany *et al.* 2011). The markers of choice are SNPs, as the description of SNP position and allelic variation is most precise and high-throughput assays can be developed for individual SNPs or SNP haplotypes (De Koeyer *et al.* 2010; Li *et al.* 2005; Li *et al.* 2013; Reed *et al.* 2007; Semagn *et al.* 2014). In addition, the question of reproducibility of a marker-trait association in different germ plasm collections is important for breeding applications and is just at the beginning to be addressed in potato (Li *et al.* 2013).

In this study, we (i) probed genes with key functions in starch-sugar interconversion for association of SNPs with tuber starch and sugar content, chip quality, tuber yield, and starch yield; (ii) cloned and functionally characterized a starch phosphorylase cDNA allele diagnostic for increased tuber starch content and better processing quality; (iii) identified SNP alleles and haplotypes corresponding to known SSCP markers diagnostic for tuber quality traits; (iv) assessed the diagnostic value of markers associated with processing quality in new germplasm; and (v) provide an overview on the genomic organization of the genes functional in starch-sugar interconversion based on the potato genome sequence (Potato Genome Sequencing Consortium *et al.* 2011).

## Materials and Methods

### Plant material and phenotypes

For association analysis of SNP markers, 208 tetraploid genotypes of the ‘CHIPS-ALL’ population (Li *et al.* 2008) were used, which consisted of 34 standard varieties and 76, 91, and 7 breeding clones from Böhm-Nordkartoffel Agrarproduktion OHG (BNA, Ebstorf, Germany), Saka Pflanzenzucht GmbH & Co. KG (Windeby, Germany), and Nordring-Kartoffelzucht-und Vermehrungs-GmbH (NORIKA, Groß Lüsewitz, Germany), respectively. This population has been evaluated in replicated trials for chip color after harvest in autumn (CQA) and after 3−4 months’ storage at 4° (CQS), for tuber yield (TY), tuber starch content (TSC), and tuber starch yield (TSY) (Li *et al.* 2008). Chip color was rated from 1 to 9, 1 corresponding to very dark, and 9 to very light chip color. High chip or processing quality corresponds to light chip color and low reducing sugar content. *Vice versa*, low processing quality corresponds to dark chip color and high reducing sugar content.

TSC was measured as specific gravity and is expressed in percent fresh weight. TY and TSY are expressed in decitons per hectar. The varieties Diana, Theresa, Saturna, and Satina included in CHIPS-ALL were used for cloning PHO1a cDNA alleles. Eighty-five varieties (panel ‘BRUISE85’) were part of a second, independent population of 205 tetraploid genotypes (the ‘BRUISE’ population) (Urbany *et al.* 2011). The BRUISE85 panel was used for correlating *PHO1* SSCP markers diagnostic for tuber quality traits in the CHIPS-ALL population with *PHO1* SSCP markers diagnostic for tuber quality traits in the BRUISE population. The BRUISE population has been phenotyped in replicated trials for tuber susceptibility to bruising (quantified as bruising index BI), yield, starch content, tuber shape, and plant maturity (Urbany *et al.* 2011). Bruising relates to the formation of dark-colored pigments upon mechanical impact. The variety panel BRUISE85 and the CHIPS-ALL standards had 11 varieties in common (Eldena, Ilona, Karlena, Kolibri, Marabel, Panda, Satina, Saturna, Solara, Tomensa, and Valisa).

The third panel, ‘SUGAR40,’ was used for validation of markers diagnostic for chip quality in the CHIPS-ALL population. It consisted of 39 varieties and one breeding clone. Twenty genotypes of the SUGAR40 panel have been pre-selected for superior and the other 20 for inferior processing quality. The SUGAR40 panel has been evaluated in triplicate samples for tuber reducing sugar content (mg/100 mg dry weight) in response to cold storage (Fischer *et al.* 2013). Six varieties (Christa, Goldika, Milva, Satina, Solara, and Solist) in the SUGAR40 panel also were included in the standard varieties of the CHIPS-ALL population.

### Amplicon sequencing and SNP calling

A total of 50 ng of genomic DNA template in 25 μL of 1x ammonium buffer (Ampliqon, Odense, Denmark), 2.5 mM MgCl_2_, 0.2 mM dNTP each, and 5 μM each forward and reverse primer were amplified with 1 U Ampliqon Taq III polymerase (Ampliqon) using the following cycling conditions: Initial denaturation 4 min at 94°, 34 cycles denaturation at 94° for 45 sec, annealing for 30 sec at the temperature specified in Table 1, elongation at 72° for 1 min per kbp, and final elongation at 72° for 6 min. Exon 3 of the *InvDE141* gene was amplified as described (Draffehn *et al.* 2010). Amplicons were custom sequenced at the Max-Planck-Genome-Center Cologne using the dideoxy chain-termination sequencing method, an ABI PRISM Dye Terminator Cycle Sequencing Ready Reaction Kit, and an ABI PRISM 3730 automated DNA Sequencer (Applied Biosystems, Weiterstadt, Germany). SNPs were detected by visual examination of the sequence trace files for overlapping base calling peaks. Biallelic SNPs were assigned in each tetraploid individual to one of five genotype classes (*AAAA*, *AAAB*, *AABB*, *ABBB*, *BBBB*). In the case of triallelic SNPs additional classes such as *AAAC* or *AABC* were assigned. The SNP allele dosage in heterozygous individuals (1:3, 2:2, or 3:1) was estimated from the height ratio of the overlapping base calling peaks manually and using the Data Acquisition and Analysis Software DAx (Van Mierlo Software Consultancy, Eindhoven, The Netherlands). For statistical analysis, genotypes were converted in numerical values from 0 to 4 (biallelic SNPs) or 0 to N (triallelic SNPs, N = number of genotype classes present in the population).

**Table 1:** Loci analyzed, primers for amplicon sequencing, cDNA cloning and allele-specific amplification, annealing temperatures (T_a_), amplicon sizes, and number of SNPs scored

Gene Acronym (GenBank Accession No.)	Locus PGSC0003DMG	Chr.	Primer Sequences 5′-3′ (* Primer Used for Amplicon Sequencing)	T_a_, °C	Amplicon Size, bp	No. SNPs and Indels Scored
*GWD* (Y09533)	400007677	V	f-ATAAAAGTCAAAGCAAAGAAGAGCCT	57	770	30
			*r-GGATAATGCCCAGTGAAGAGTAAT			
*PWD* (AY747068)	400016613	IX	*f-GGTCTGATGATCTATCTGATTGC	57	871	29
			r-GACATCTTGAGGAGAACCAAACTT			
*BMY-8/2* (AF393847)	400001855	VIII	*f-GCTACTGGA(Ino)CATGGTGACAGA*^a^* r-TTACATAGAGGTCTGTCCTGCTTGAG	57	560	14
*PHO1b* (*StpL*) (X73684)	400028382	V	f-TGTTGCAAGAAAAGCTAAACCAA	57	1178	38
			*r-GATCACCAATCTCGGGATCA			
*SssI* (Y10416)	401018552	III	f-GGATACTCATGGGAAATAACAACTCC	57	1022	10
			*r-CAATCAGGTCGAATTGGAAGG			1 indel
*PGM-3* (AJ240053)	Not annotated	III	*f-ATGGCTATGGAGAGTGCATTGA	57	1060	15
			r-GTATCCAATTGGCAAGGTAATTGTC			
*AGPaseS* (X61187)	400000735	I	*f-GGAGATGGATTTGTCGAGGT	56	933	25
			r-TGACCAGCCCAAAATCTGAT			
*INV-8/2* (NM_001247140)	400004790	VIII	*f-GTTCTCATCCCACCACCCG	56	1096	15
			r-CGTTCACCAGATCCACCACTC			
*PHO1a* (*Stp23*) (X52385, D00520)	Not annotated	III	f-ATCACTCTCATTCGAAAAGCTAGAT	65	2976	-*^b^*
			r-TGCCTTTGTTATTTTTCATTCACTTC			
			f-GCTAGATTTGCATAGAGAGCACA	66	3031	-*^b^*
			r-GTGGTAATAACATCATCCTCTTACAC			
			*f-GAGGACCGAACACAACACACTT	59	2100	C_22_T
			r-CACCTCCTCCTGACCATCTT			
			*f-GCGACCTGAGTTCTTTTGCT	59	1400	G_322_A
			r-CATGCTTTGGTGTGCTCTCC			
			f-AGTTCTTTTGCTCCTGATGCC	59–54 *^c^*	-*^d^*	G_824_
			r-GAACATCATACGCAACTGTCC			
			f-TGAAATAAGGGAAGAGGTTGGA	59–54 *^c^*	-*^d^*	A_2776_
			r-AACAAACTGGACTTACTTTCTGATT			
			f-CTTAGAAAAGAAAGAGCTGGCA	59–54*^c^*	-*^d^*	A_2578_
			r-CAAAGTCAATAGCATACACAAAACAT			

SNP, single-nucleotide polymorphism; PCR, polymerase chain reaction.

a Modified from (Krusiewicz *et al.* 2011)

b Primers for cDNA synthesis.

c Touch down PCR with ΔT° of −0.5°C.

d Primers for SNP allele specific amplification.

### Allele-specific polymerase chain reaction (PCR) assays

Allele-specific PCR assays for the markers *Stp23-8b*, *StpL-3b*, *StpL-e3*, *Pain1-8c*, and *InvGE-6f* were performed as described previously (Li *et al.* 2005, 2013). The alleles were scored as present (1) or absent (0) without considering allele dosage. Specific SNP alleles in the *PHO1a* gene were amplified from 40 ng of genomic DNA template in 25 μL of total volume containing 1x PCR buffer (10 mM Tris-HCL pH 8.3; 50 mM KCl; 1.5 mM MgCl_2_; 0.1% Triton X-100), 0.2 mM of each dNTP, 1 μM each forward and reverse primers (Table 1), and 1.5 U *Taq*-Polymerase. PCR conditions were as follows: Initial denaturation for 2 min at 94°; 10 cycles of touch down PCR: 30 sec denaturation at 94°, 30 sec annealing at 59°, decreasing T_a_ by 0.5° per cycle, 1 min per kbp extension at 72°; then 25 cycles as before with constant annealing at 54°; 5 min final extension at 72°. Presence or absence of the SNP allele was scored as described previously.

### Cloning and sequencing of *PHO1a* cDNA alleles

Total RNA was extracted from tubers and leaves of the tetraploid varieties Diana, Theresa, Saturna, and Satina. Leaf RNA was isolated using the ToTALLY RNA Total RNA Isolation Kit (Ambion, Austin, TX). Tuber RNA was isolated either with the PureLink Plant RNA Reagent (Invitrogen Life Technologies, Carlsbad, CA) or using the protocol described in Kumar *et al.* (2007). Total RNA was eluted in diethylpyrocarbonate-treated water and quantified with a Qubit fluorometer (Invitrogen Life Technologies) or a NanoDrop UV-Vis spectrophotometer (Thermo Scientific). Contaminating DNA was removed with DNaseI using the DNA-free Kit (Ambion). First-strand cDNA was synthesized from 1 μg of total RNA using the Transcriptor First Strand cDNA Synthesis Kit (Roche Applied Science, Mannheim, Germany) according to the manufacturer’s protocol and anchored-oligo (dT)_18_ primers and one sequence specific primer, or two sequence-specific primers (Table 1). PHO1a cDNA was selectively amplified from ca. 40 ng of cDNA template in 25 μL of PCR buffer (10 mM Tris-HCL pH 8.3; 50 mM KCl; 1.5 mM MgCl_2_; 0.1% Triton X-100) including 0.2 mM each dNTP, 1 μM each forward and reverse primers, and 1.5 U of Taq-Polymerase (KAPA2G Fast PCR Kit (PEQLAB Biotechnology, Erlangen, Germany) or FastStart High Fidelity PCR System (Roche) or AccuPrime Pfx DNA Polymerase (Invitrogen Life Technologies). Cycling conditions were as follows: initial denaturation for 2 min at 94°, 20 cycles of denaturation (30 sec at 94°), annealing (30 sec at Ta according to Table 1) and extension (1 min/kb at 72°), and final extension for 5 min at 72°. Products of two to three independent PCRs per genotype and tissue (19 in total) were cloned in *Escherichia coli* plasmid vector TOPO XL (Invitrogen Life Technologies) and transformed into competent cells of strains DH5α or One Shot *ccd*B Survival (Invitrogen Life Technologies) according to the manufacturer’s instructions. Plasmid DNA was isolated using the Plasmid Mini Kit (QIAGEN, Hilden, Germany). Plasmid insertions were custom Sanger sequenced at the Max-Planck-Genome-Center Cologne on Abi Prism 377, 3100 and 3730 sequencers (Applied Biosystems) using BigDye-terminator v3.1 chemistry. Sequences were aligned and examined for SNPs. An SNP was considered factual when it was detected in at least three clones originated from two independent amplifications.

### Native polyacrylamide gel electrophoresis (PAGE) and Pho1a enzyme activity test

Approximately 1 g of deep frozen tuber tissue was homogenized in 500 μL of grinding buffer (100 mM HEPES-NaOH, pH 7.5; 1 mM ethylenediaminetetraacetic acid; 5 mM dithiothreitol; 10% [v/v] glycerol). The homogenate was centrifuged at 14,800 g for 15 min at 4°. The supernatant was removed, and total protein was quantified with a *Qubit* Fluorometer (Invitrogen) and stored at −20°. A total of 50 µg of total native protein were loaded onto NativePAGE Novex 3–12% Bis-Tris gels (Life Technologies) and electrophoresed at 200 V and 4° in a XCell *SureLock* Mini-Cell (Invitrogen), following the manufacturer’s instructions. Glucan-forming activity of phosphorylase was tested as described (Manchenko 2003). After electrophoresis gels were placed for 15 min at room temperature in 100 mM citrate-NaOH (pH 6.5), and then incubated for at least 4 hr at 37° in 100 mM citrate (pH 6.5), 20 mM glucose-1-phosphate, and 0.2% (w/v) soluble starch or glycogen. Glucans formed in the gel were stained with iodine (Lugol solution).

### Databases and software tools

Potato and tomato candidate gene and cDNA sequences were retrieved from NCBI (http://www.ncbi.nlm.nih.gov/) and SPUD DB Potato Genomics Resource (http://solanaceae.plantbiology.msu.edu/pgsc_download.shtml) by text searches. Annotated potato loci (PGSC0003DMG40*******) corresponding to the genes were obtained using the BLAST sequence alignments tool in (http://potato.plantbiology.msu.edu/integrated_searches.shtml). Gene number, genomic position (pseudomolecules v4.03), and expression profiles were retrieved from the potato genome browser *S. tuberosum* group Phureja DM1-3 (http://potato.plantbiology.msu.edu/cgi-bin/gbrowse/potato/). Exon-intron structure was deduced from aligning cDNA with genomic sequence using Spidey (http://www.ncbi.nlm.nih.gov/spidey/) or from RNAseq data available in the potato genome browser. cDNA sequences were assembled and analyzed using the software tools BioEdit (Hall 1999), ClustalW2 and ClustalX2 (Larkin *et al.* 2007), DNASTAR Lasergene 8 (DNASTAR inc, Madison WI), MEGA4 (Tamura *et al.* 2007), and NovoSNP (Weckx *et al.* 2005). Multiple polypeptide alignments were performed with MUSCLE (Edgar 2004). The structure of PHO1a was modeled with SWISS-MODEL (Arnold *et al.* 2006; Guex *et al.* 2009) using as template the crystal structure of yeast glycogen phosphorylase (PDB entry 1YGP). Effects of amino acid substitutions were predicted with the AUTO-MUTE algorithm (Masso and Vaisman 2010) based on the structure of rabbit glycogen phosphorylase B (PDB entry 6GBP).

### Statistical analyses

Association analysis of SNPs scored in the CHIPS-ALL population was performed as described previously (Li *et al.* 2008, 2013). A threshold of *P* < 0.01 for at least one of the five traits analyzed was adopted for reporting an association between a SNP marker and one or more tuber traits. Effects are reported for the minor frequency allele (MFA). SNPs with a MFA <1% (MFA was present in simplex in less than eight genotypes of the CHIPS-ALL population) were not considered. Markers associated with chip quality in the CHIPS-ALL population were tested by analysis of variance for reproducibility of the MFA effect in the SUGAR40 panel. Three measurements of tuber reducing sugar content (mg/100 mg dry weight) in the SUGAR40 panel (Fischer *et al.* 2013) were log10 transformed to obtain normal distributions. Markers with a minor allele frequency <2% (MFA was present in simplex in less than three genotypes) were not considered. SPSS 15.0 software (IBM) was used for all analyses. Linkage disequilibrium (LD) between 273 pairs of SNP markers was estimated with a χ^2^ test based on the SNP allele frequencies. *P*-values were corrected for multiple testing according to Storey and Tibshirani (2003). The analysis was performed with R (R Development Core Team 2013) using a custom made script.

## Results

### SNPs in candidate genes diagnostic for tuber quality traits in the CHIPS-ALL population

Gene specific primers suitable for amplicon sequencing were designed for eight genes with important functions in starch-sugar interconversion according to the literature (Table 1 and Figure 1). Position and nucleotide alleles of 176 SNPs and one indel scored in the eight amplicons are shown in Supporting Information, File S1. Associations with tuber quality traits CQA, CQS, TSC, TY, and TSY are reported in Table 2. LD was estimated between 273 pairs of SNPs, including the 176 SNPs described in this paper plus the SNPs scored previously in the invertase genes *Pain-1*, *InvGE*, and *InvCD141* (Draffehn *et al.* 2010), the Kunitz-type invertase inhibitor *KT-InvInh* and leucine aminopeptidase *LAP* (Fischer *et al.* 2013) (File S2).

**Table 2:** Associations of SNP alleles with CQA and CQS, TSC, TY, and/or TSY in the CHIPS-ALL population

SNP*^a^*	Minor Allele Frequency, %	CQA Percent Variance Explained*^b^*	CQS Percent Variance Explained*^b^*	TSC Percent Variance Explained*^b^*	TY Percent Variance Explained*^b^*	TSY Percent Variance Explained*^b^*
*GWD-A_3259_G* (*A_3072_T*, *C_3400_G*)	6.6 (*G*)	ns	ns	3.6* ↑*^c^*	ns	4.6** ↑
*GWD-A_3452_G*	2.6 (*G*)	ns	ns	5.9** ↑	2.6* ↓	4.4** ↑
*PWD-T_10547_C* (*C_10911_T*)	49.0 (*C*)	ns	ns	7.2** ↑	ns	4.4* ↑
*PWD-G_11140_A* (*G_11112_A*)	2.2 (*A*)	3.4** ↑	7.3*** ↑	ns	2.2* ↓	ns
*PWD-G_10758_A*	3.8 (*A*)	5.6*** ↓	ns	2.1* ↓	ns	ns
*PWD-T_11148_A*	1.0 (*A*)	ns	2.3* ↑	3.9** ↑	ns	2.4* ↑
*BMY1-G_2533_A*	30.1 (*A*)	ns	ns	4.6** ↓	ns	3.0* ↓
*BMY1-G_2565_A*	1.5 (*A*)	2.8** ↑	3.5** ↑	7.9*** ↑	ns	4.5** ↑
*BMY1-C_2649_A* (*A_2694_G*, *C_2811_T*)	6.9 (*A*)	4.5** ↓	6.5** ↓	7.2** ↓	ns	4.4* ↓
*BMY1-G_2657_A* (*G_2767_A*)	10.0 (*A*)	3.4* ↑	7.1** ↑	7.5** ↑	ns	6.7** ↑
*BMY1-G_2751_A*	5.1 (*A*)	ns	5.6** ↓	4.3** ↓	ns	4.2** ↓
*PHO1b-A_3931_G* (*C_4206_G*)	1.5 (*G*)	3.3** ↓	ns	ns	ns	ns
*PHO1b-A_3982_C*	27.6 (*C*)	3.1* ↑	9.9*** ↑	8.9*** ↑	ns	3.8* ↑
*PHO1b-C_3990_T* (*G_4049_A*)	8.8 (*T*)	2.8* ↑	7.0** ↑	ns	4.7* ↓	ns
*PHO1b-G_4181_A*	19.7 (*A*)	3.4* ↑	7.2** ↑	6.2* ↑	ns	ns
*PHO1b-G_4106_A* (*G_4109_C*)	23.1 (*A*)	3.4* ↑	5.6** ↑	ns	ns	ns
*PHO1b-A_4207_GT*	18.0 (*GT*)	ns	8.8** ↑	4.8* ↑	5.2* ↓	ns
*PHO1b-AT_4207_G*	16.0 (*G*)	ns	7.0** ↑	ns	5.4* ↓	ns
*PHO1b-T_4404_C* (*A_4365_G*)	11.8 (*C*)	ns	5.7** ↓	9.3*** ↓	ns	4.9* ↓
*SssI-A_5858_G*	26.1 (*G*)	ns	ns	5.9** ↑	ns	ns
*PGM-A_408_T*	13.7 (*T*)	ns	ns	6.8** ↑	ns	4.8* ↑
*PGM-T_441_AC*	8.0 (AC)	ns	ns	ns	8.2** ↓↑*^c^*	ns
*PGM-T_468_C*	38.0 (*C*)	ns	ns	10.5*** ↑	ns	6.3* ↑
*AGPaseS-T_1259_C* (*T_1612_C*)	8.5 (*C*)	7.4*** ↓	10.1*** ↓	19.9*** ↓	ns	10.3*** ↓
*AGPaseS-G_1592_C* (*A_1348_G*)	20.6 (*C*)	ns	5.4*↓	15.0*** ↓	ns	7.8**↓
*AGPaseS-T_1284_C* (*C_1411_T*, *G_1457_C*)	5.0 (*C*)	1.6* ↑	8.3*** ↑	6.9*** ↑	ns	2.3* ↑
*AGPaseS-A_1286_G*	31.6 (*G*)	5.1** ↓	5.8** ↓	7.9** ↓	5.5* ↑	ns
*AGPaseS-T_1392_C*	34.2 (*C*)	ns	ns	9.0** ↑	ns	5.1* ↑
*INV-8/2-T_2076_C* (*A_2065_G*, *T_2272_A*)	37.6 (*C*)	7.8*** ↓	7.7** ↓	9.2*** ↓	ns	8.0** ↓
*INV-8/2-T_2134_G* (*G_2116_A*)	41.7 (*G*)	5.3** ↑	5.8* ↑	4.4* ↑	4.8* ↓	5.3* ↑
*INV-8/2-T_2320_C* (*G_2255_A*)	13.0 (*C*)	2.1* ↑	3.0* ↑	7.2*** ↑	2.8* ↓	ns
*INV-8/2-G_2182_A*	16.2 (*A*)	4.6** ↑	4.3* ↑	9.2*** ↑	3.9* ↓	ns

SNP, single-nucleotide polymorphism; CQA, chip color after harvest in autumn; CQS, chip color after 3 months storage at 4°; TSC, tuber starch content; TY, tuber yield; TSY, tuber starch yield; ns, not significant, MFA, minor frequency allele.

a SNPs in parentheses were in strong LD, showed similar associations and the same direction of effect as the SNP, for which the data are shown.

b Significance is indicated by “ns” (*P* > 0.05), *0.05 > *P* ≥ 0.01, **0.01 > *P* ≥ 0.001 and ***P < 0.001.

c Arrows indicate the direction of the effect of the MFA on the trait, upwards for a positive (lighter chip color corresponding to lower sugar content, greater starch content, yield, starch yield) and downward for a negative effect (darker chip color corresponding to greater sugar content, lower starch content, yield, starch yield). ↑↓: direction of effect was inconsistent between genotypic groups.

1. Glucan water dikinase (*GWD*; Lorberth *et al.* 1998) is a single copy gene in the potato genome, 15.6 kbp long with 33 exons, which is expressed in all tissues. Four of 30 SNPs scored in an amplicon spanning exons seven to nine were associated with TSY and TSC, three of which were in near-complete LD (Table 2 and File S2). The representative minor frequency SNP allele *GWD-G_3259_* had a positive effect on TSY. The rare (frequency 2–3%) SNP allele *GWD-G_3452_* increased TSC and TSY (Table 2).
2. Phosphoglucan water dikinase *PWD* (Baunsgaard *et al.* 2005) has not been functionally characterized in potato. *PWD* is a singular, 11.9-kbp gene in the potato genome, has 19 exons, and is ubiquitously expressed. The amplicon generated from exon 19 identified 29 SNPs, six of which showed trait associations (Table 2). The most frequent haplotype *PWD-C_10547_T_10911_* was associated with increased TSC. Interestingly, the *PWD* allele diagnosed by these SNPs seemed to be recessive, as the positive effect was observed only in the genotypic class homozygous for *PWD-C_10547_T_10911_* (not shown). The rare haplotype *PWD-A_11112_A_11140_* was associated with lighter chip color, particularly after cold storage, whereas another low-frequency allele, *PWD-A_10758_*, showed the opposite effect.
3. The plastidic beta-amylase *BMY-8/2* is one of nine potato genes annotated as beta-amylase (Nielsen *et al.* 1997; Scheidig *et al.* 2002) and one of two closely related *BMY* paralogous genes on chromosome VIII (PGSC0003DMG400001855, PGSC0003DMG402020509). *BMY-8/2* is 3.6 kbp long, has four exons, and is highly expressed in most tissues. Eight of 14 SNPs scored in exon four showed trait associations (Table 2). The rare allele *BMY-8/2-A_2565_* and the minor frequency haplotype *BMY-8/2-A_2657_A_2767_* were both associated with lighter chip color and greater tuber starch content and starch yield. Both the low-frequency alleles *BMY-8/2-A_2649_* and *BMY-8/2-A _2751_* had inverse effects on the same traits. The most frequent allele *BMY-8/2-A_2533_* affected negatively TSC.
4. *PHO1b* [*StpL* in (Li *et al.* 2008. 2013)] is one of two related, plastidic L-type starch phosphorylase genes, *PHO1a* and *PHO1b*, in the potato genome (Albrecht *et al.* 2001; Sonnewald *et al.* 1995). It is 6.2 kbp long, has 14 exons, and is highly expressed in leaves, flowers, shoots, stems, and stolons but is undetectable in roots, tubers, and sprouts. The sequenced amplicon spanned exons seven to nine and was highly polymorphic. Eleven of 38 SNPs showed associations with chip color and TSC (Table 2). The most frequent SNP alleles all had positive effects on the traits. Allele *PHO1b-C_4404_* showed negative effects. The distributions of *PHO1b-C_4404_* and the SSCP marker *StpL-3c* (Li *et al.* 2008) in the CHIPS-ALL population were 89% identical (not shown), indicating that *PHO1b-C_4404_* and *StpL-3c* diagnose the same *PHO1b* allele. The 11% discrepancies are likely the result of scoring errors than can occur in SSCP analysis as well as in amplicon sequencing. Despite the large number of SNPs scored, SNPs corresponding to the diagnostic SSCP markers *StpL-3b* and *StpL-3e* (Li *et al.* 2008. 2013) were not found in this amplicon. Allele-specific assays for *StpL-3b* and *StpL-3e* , however, have been designed based on SNPs in the 1.3 kbp N-terminal region of the locus, which included exons one and two (Li *et al.* 2013) (Table 3). SSCP markers derived from *PHO1b* also were associated with tuber susceptibility to bruising and starch content in the independent BRUISE association mapping population (Urbany *et al.* 2011). To assess whether the diagnostic *PHO1b* alleles were the same in the CHIPS-ALL and BRUISE populations, the BRUISE85 variety panel was genotyped for *StpL-3b* and *StpL-3e* using the allele specific assays. *StpL-3b* corresponded to the SSCP marker *PHO1B-1b* associated with decreased bruise susceptibility and tuber starch content in the BRUISE population (Urbany *et al.* 2011), which is in accordance with the effect of *StpL-3b* on TSC in the CHIPS-ALL population (Li *et al.* 2008) (Table 3). No correspondence was found between *StpL-3e* and *PHO1b* SSCP markers positively associated with TSC in the BRUISE population.

**Table 3:** Correspondence between diagnostic SSCP markers, cDNA alleles, and SNP alleles or haplotypes

Locus	SSCP Marker (cDNA Allele)	Corresponding SNP Allele or Haplotype	Reference
PGSC0003DMG400013856	*Pain1-9a* (*Pain1-a*) *↑**^a^*	*Pain1-A_1544_**^b^*	(Draffehn *et al.* 2010)
PGSC0003DMG400013856	*Pain1-8c* (*Pain1-a*) *↑*	*Pain1-C_552_A_718_**^b^*	(Draffehn *et al.* 2010)
PGSC0003DMG400000735	*AGPsS-9a ↑*	*AGPaseS-C_1284_T _1411_C_1457_**^c^*	This paper
PGSC0003DMG400000735	*AGPsS-10a* ***↓***	*AGPaseS-C_1259_C_1612_**^c^*	This paper
Not annotated	*Stp23-8b*, *PHO1A-c* (*PHO1a-H_A_*) *↑*	*PHO1a-T_22_A _322_G_824_A_2776_**^b^*	This paper
PGSC0003DMG400028382	*StpL-3b*, *PHO1B-1b* ***↓***	*PHO1b-A_158_C_159_ A_1250_**^c^*	(Li *et al.* 2013)
PGSC0003DMG400028382	*StpL-3e ↑*	*PHO1b-G_1305_T_1311_**^c^*	(Li *et al.* 2013)
PGSC0003DMG400028382	*StpL-3c* ***↓***	*PHO1b-C_4404_**^c^*	This paper
PGSC0003DMG400008942	*InvGE-6f* (*InvGE-a*) *↑*	*InvGE-A_1103_**^b^*	(Draffehn *et al.* 2010)
PGSC0003DMG402028252	*pCD141-3c* (*InvCD141-Sa*) ***↓***	*InvCD141-A_280_T_288_T_339_T_543_A_630_C_1030_G_1031_T_1096_**^b^*	(Draffehn *et al.* 2010)

SSCP, single-strand conformation polymorphism; SNP, single-nucleotide polymorphism.

a The arrow indicates the direction of the allele effect on the traits, upwards for a positive (lower sugar content = greater chip quality, greater starch content) and downward for a negative effect (greater sugar content = lower chip quality, lower starch content).

b SNPs are numbered based on cDNA alleles, position 1 being A in the ATG start codon.

c SNPs are numbered based on the DM genomic sequence, position 1 being A in the ATG start codon.
5. Soluble starch synthase I (*SssI*) (Kossmann *et al.* 1999) is one of eight potato genes annotated as starch synthase, has a length of 9 kbp, 15 exons, and is ubiquitously expressed. Ten SNPs and one indel were scored in an amplicon spanning exons nine and ten. One minor frequency SNP allele had a positive effect on TSC (Table 2). None of the DNA variants in the amplicon corresponded to the SSCP marker *SssI-4b* that was associated with lighter chip color and greater TSC and TSY in the CHIPS-ALL population (Li *et al.* 2008).
6. Besides two genes annotated as plastidic phosphoglucomutase (Tauberger *et al.* 2000), the third plastidic *PGM-3* analyzed in this study (AJ240053) is not annotated in the current version (4.03) of the potato genome. Aligning the full-length cDNA with the genome sequence revealed that *PGM-3* is located in a 16.2-kbp region on chromosome III (Table S1). The gene has at least 21 exons and is expressed in all tissues. The sequencing of an amplicon spanning exons one to three revealed 15 SNPs including two triallelic SNPs. Three SNPs showed associations, in particular the common allele *PGM-3-C_468_*, which was associated with increased TSC. The triallelic SNP marker *PGM-3-T_441_ AC* was the only one in the whole set, which showed above threshold significant association with tuber yield. The direction of the effect, however, was inconsistent between genotypic groups (Table 2).
7. ADP-glucose pyrophosphorylase S (*AGPaseS*) is one of three expressed genes encoding the large (L) subunit of glucose-1-phosphate adenylyltransferase (Tiessen *et al.* 2002) in the potato genome (Table S1). The 5-kbp gene has 14 exons and is ubiquitously expressed. Twenty-five SNPs were scored in an amplicon located in exons three to six. Nine SNPs in five LD groups were associated with chip color, TSC, and TSY. The low-frequency SNP haplotypes *AGPaseS-C_1259_C_1612_* and *AGPaseS-C_1284_T_1411_C_1457_* corresponded to the diagnostic SSCP markers *AGPsS-10a* and *AGPsS-9a* (Li *et al.* 2013), respectively (Table 3). The three remaining, rather frequent SNP alleles showed negative (*AGPaseS-G_1286_* and *AGPaseS-G_1348_ C_1592_*) or positive (*AGPaseS-C_1392_*) trait associations (Table 2).
8. Twenty genes are annotated either as ‘invertase’ or ‘beta-fructofuranosidase’ in the potato genome (Table S1), five of which have been previously cloned and characterized, the soluble acid invertase *Pain-1* (Zhou *et al.* 1994; Zrenner *et al.* 1996) and two pairs of tandem duplicated apoplastic or cell wall invertases *InvGE* and *InvGF* (Maddison *et al.* 1999), and *InvCD111* and *InvCD141* (Draffehn *et al.* 2010; Fridman and Zamir 2003; Hedley *et al.* 1994). Associations of SSCPs and corresponding SNPs with tuber quality traits have been identified in all three loci (Draffehn *et al.* 2010; Li *et al.* 2005, 2008) (Table 3). The closest homolog of *Pain-1* with the same exon-intron structure is the locus PGSC0003DMG400004790 (*INV-8/2*) on chromosome VIII. This putative invertase gene, which is orthologous to the equally uncharacterized tomato gene *LIN9* (http://www.ncbi.nlm.nih.gov/gene/778304), has not been functionally characterized so far. It has a length of 4.1 kbp, seven exons, and is expressed mainly in flowers. Expression in tubers is very low (Schreiber 2012). Eight of 15 SNPs in an amplicon spanning exons three to five were associated with chip color, TSC, and/or TSY. The frequent SNP allele *INV-8/2-C_2076_* had negative effects, and SNP alleles *INV-8/2-G_2134_*, *INV-8/2-C_2320_*, and *INV-8/2-A_2182_* had positive effects (Table 2).

LD between all SNPs scored in the CHIPS-ALL population was strongest between SNPs within the same locus. Strong LD blocks included SNPs with physical distances up to approximately 700 bp. Weaker LD was observed between physically linked loci such as *Pain-1*, *PGM-3*, *SssI*, and *KT-InvInh* on chromosome III but with similar intensity compared to LD between unlinked loci (File S2). With one exception (*PGM-3-snp408* and *KT-InvInh-snp395/396* on chromosome III), SNPs associated with tuber quality traits showed very low or no LD among each other. This indicated that most of the observed associations were independent from each other.

### Diagnostic SNPs in full-length cDNA alleles of starch phosphorylase *PHO1a*

The gene *PHO1a* (*Stp23* in (Li *et al.* 2008, 2013)) on potato chromosome III (Chen *et al.* 2001), is annotated as four different loci in the current version of the potato genome sequence (exon 1: PGSC0003DMG400033858, exons 2−5: PGSC0003DMG400003495, exons 6−13: PGSC0003DMG400007782, exons 13−15: PGSC0003DMG400002479) (Table S1). The characterized cDNA sequence (D00520) matches to four small scaffolds containing different parts of the gene (PGSC0003DMO000067817, PGSC0003DMO000068066, PGSC0003DMO000068164, PGSC0003DMO000068105), which are not anchored to the current physical map. The gene comprises 15 kbp, has 15 exons, and is highly expressed in all tissues. In total 161 full-length cDNAs (2901 bp) were cloned from leaf and tuber tissue of cultivars Diana, Theresa, Saturna, and Satina. Satina served as negative control because this cultivar lacked the SSCP markers *Stp23-8b* and *Stp23-8a* associated with increased tuber starch content and better chip quality in the CHIPS-ALL population (Li *et al.* 2008). Multiple sequence comparisons identified 15 consensus SNPs, eight of which caused amino acid changes. Based on the 15 SNPs, nine cDNA variants of *PHO1a* were distinguished (Table 4 and Figure S1). The sequence of cDNA 1 was obtained from all four cultivars and was identical to GenBank accessions D00520 and X52385 (Table 1). We refer to this sequence as the reference allele *PHO1a-H_R_*. The reference allele was the only one retrieved from cv Satina, suggesting that Satina was homozygous for *PHO1a-H_R_*. The remaining eight cDNA sequences fell into two groups, cDNAs 2, 3, 4, 5, 6, and 7, 8, 9 (Table 4).

**Table 4:** SNPs and amino acid changes in PHO1a full-length cDNA alleles 1–9 isolated from cv Diana, Theresa, Saturna, and Satina

SNP*^a^*	Amino Acid Change	1 H_R_*^b^*	2	3	4	5 H_A_*^b^*	6	7	8	9
C_22_T	His8Tyr	C	T	T	T	T	T	C	C	C
G_322_A	Ala108Thr	G	A	A	A	A	A	G	G	G
C_504_T	−	C	C	C	C	C	C	T	T	T
A_534_G	−	A	G	G	G	G	G	G	G	G
A_720_C	−	A	C	C	C	C	C	A	A	A
A_824_G	Lys275Arg	A	A	A	G	G	G	A	A	A
G_1923_T	−	G	G	G	G	T	T	G	G	G
G_1939_T	Ala647Ser	G	G	G	G	T	T	G	G	G
G_2578_A	Gly860Arg	G	G	G	G	G	G	A	A	A
G_2616_A	−	G	G	G	G	G	G	G	G	A
G_2754_A	−	G	G	G	G	G	G	A	A	G
A_2765_T	Glu922Val	A	A	A	A	A	A	A	A	T
G_2776_A	Asp926Asn	G	G	A	G	A	G	G	G	G
C_2795_A	Thr923Lys	C	A	A	C	A	A	A	A	A
G_2823_C	−	G	C	C	G	C	C	C	G	G

SNP, single-nucleotide polymorphism.

a SNPs are numbered based on the cDNA, position 1 being A in the ATG start codon.

b cDNA sequence 1 corresponded to the reference allele H_R_ (GenBank accessions D00520, X52385), whereas cDNA sequence 5 corresponded to the allele H_A_ associated with increased tuber starch content.

To identify cDNAs corresponding to SSCP markers *Stp23-8b* and *Stp23-8a*, which are both diagnostic for increased chip quality, TSC, and TSY (Li *et al.* 2008), the 34 standard varieties of the CHIPS-ALL population were scored for five SNPs, C_22_T, and G_322_A by amplicon sequencing, G_824_, A_2578_, and A_2776_ by nucleotide specific amplification (Table 1). The A_2578_-specific assay diagnostic for cDNAs 7, 8, and 9 (Table 4) neither corresponded to *Stp23-8b* nor *Stp23-8a* (not shown). In contrast, the distributions of the haplotype *PHO1a-T_22_A_322_G_824_A_2776_* and the *Stp23-8b* marker in the 34 varieties were, with one exception, identical, indicating that cDNA 5, subsequently referred to as *PHO1a-H_A_*, corresponded to *Stp23-8b* (Table S2). Eleven standard varieties were simplex for the *PHO1a-H_A_* allele. Only cv Saturna contained *PHO1a-H_A_* in duplex dosage. None of the cDNAs corresponded to SSCP marker *Stp23-8a*. Genotyping the BRUISE85 panel with the allele specific marker *Stp23-8b* (Li *et al.* 2013) revealed correspondence with the SSCP marker *PHO1A-c* (Table 3), which was associated in the BRUISE population with increased tuber starch content, similarly as in the CHIPS-ALL population, but also with increased susceptibility to tuber bruising (Urbany *et al.* 2011).

### Functional characterization of *PHO1a-H_A_*

Allele-specific expression analysis in tubers during cold storage did not reveal clear differences in PHO1a transcript levels that could be attributed to the presence of the *PHO1a-H_A_* allele (Nader Nieto 2011). The presence of the protein isoform encoded by the *PHO1a-H_A_* allele was confirmed by two-dimensional gel electrophoresis and peptide mass spectrometry of total tuber protein of cultivar Saturna containing the allele in duplex dosage (Nader Nieto 2011). Alignment of the deduced PHO1a-H_A_ protein sequence with starch phosphorylase proteins from other plant species (dicots and monocots) (Figure S2) showed that the amino acid substitutions at positions 8, 275, 647 and 932 were not specific for PHO1a-H_A_, while Thr108 and Asn926 were unique for PHO1a-H_A_. Sequence alignments of more than 200 alpha-glucan phosphorylases from plants, animals, fungi and bacteria (not shown) confirmed that the nonconservative substitution of alanine108 by threonine was exclusively present in PHO1a-H_A_. Crystal structures of plant PHO1a proteins are not available. *In silico* simulation of PHO1a structure based on the crystal structure of yeast glycogen phosphorylase indicated that the Ala108Thr substitution is located in the α2 helix, a conserved region involved in the interaction between the dimers of the bacterial ortholog maltodextrin phosphorylase MALP (Watson *et al.* 1997).

**Figure 2:**
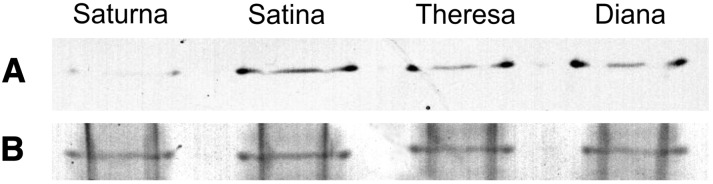
In gel PHO1a activity in tetraploid potato varieties with zero (Satina), one (Theresa, Diana), and two (Saturna) dosages of the diagnostic *PHO1a-H_A_* allele. (A) activity staining; (B) protein loading control.

The possible effect of the Ala108Thr substitution was assessed *in silico* based on the structure of rabbit glycogen phosphorylase B (Acharya and Johnson 1990). The substitution of Ala56 corresponding to Ala108 in potato PHO1a-H_A_ with threonine resulted with 84% confidence in a decrease of protein stability. PHO1a enzyme activity was analyzed in tubers of cultivars Saturna, Satina, Theresa, and Diana before cold storage. These cultivars have different allele dosages of *PHO1a-H_A_*. Saturna contained the *PHO1a-H_A_* allele in duplex, Theresa and Diana in simplex dosage, and Satina lacked the allele. Total soluble protein extracted from tubers was analyzed for glycogen forming activity on native PAGE gels (Figure 2). Very low enzyme activity was detected in tubers of cv Saturna compared with cv Satina, whereas cvs Theresa and Diana showed intermediate activity.

### Marker validation in the SUGAR40 panel

**Figure 3:**
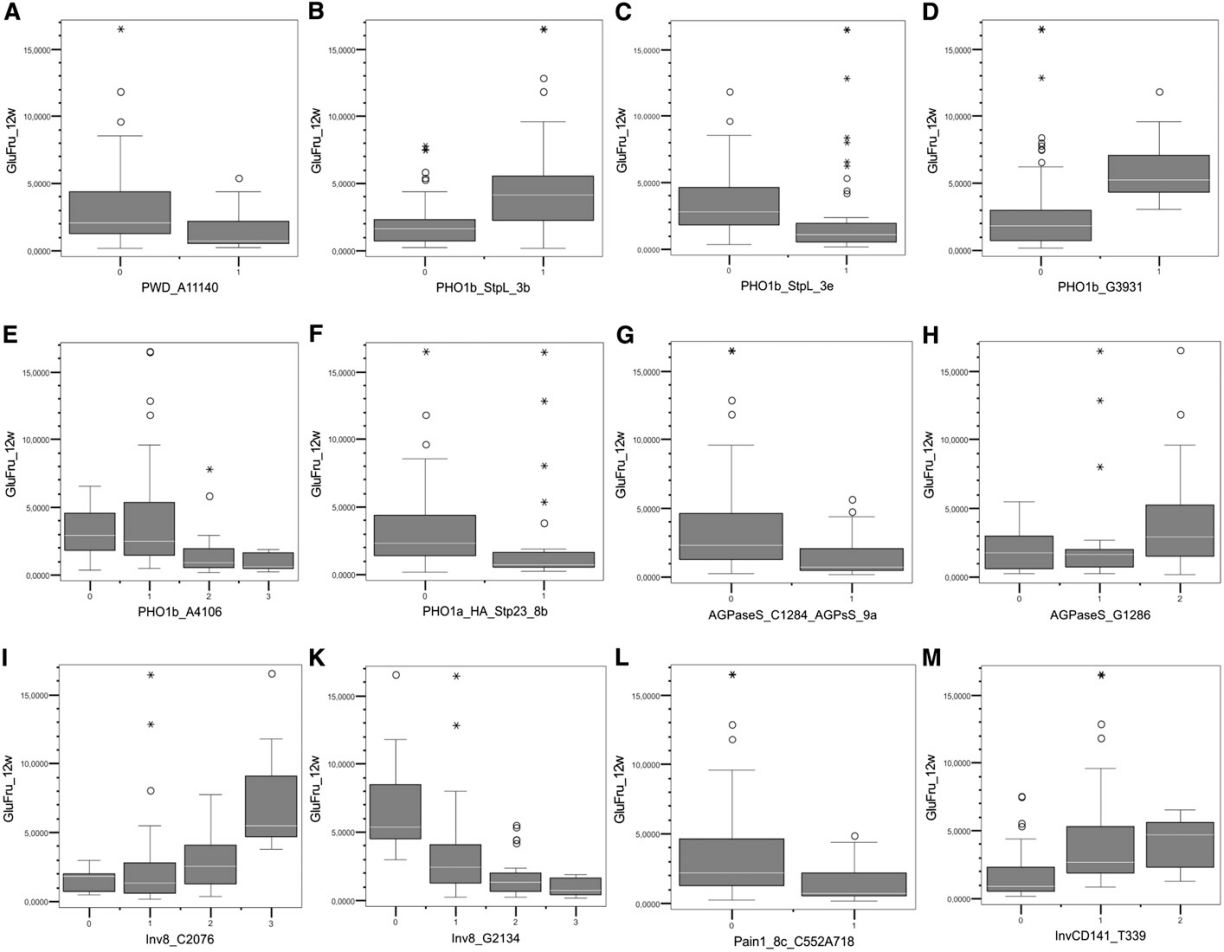
Box plots showing the effects of the marker genotypic classes of 12 candidate gene alleles on tuber reducing sugar content after 12 wk of cold storage in the SUGAR40 panel (Table 5). Y-axis: Values for reducing sugar content (glucose plus fructose in mg/100 g dry weight). X-axis: Presence (1) or absence (0) of the allele indicated (B, C, F, L) or allele dosage from 0 to 3 (A, D, E, G, H, I, K, M). Genotype classes represented by one cultivar only are not shown.

Markers derived from genes functional in starch-sugar interconversion, which were associated with chip quality in the CHIPS-ALL population in the present study (Table 2) as well as in previous studies (Draffehn *et al.* 2010; Li *et al.* 2005; 2008; 2013), were genotyped in the SUGAR40 panel, either by allele-specific assays (*Stp23-8b = PHO1a-H_A_*, *StpL*(*PHO1b*)*-3b*, *StpL*(*PHO1b*)*-3e*, *Pain1-8c*, *InvGE-6f*) or by amplicon sequencing (*PWD*, *PHO1b*, *AGPaseS*, *BMY-3*, *Inv-8/2*, *InvCD141*). Significant effects on reducing sugar content (*P* ≤ 0.05) were observed for 23 marker alleles in all genes tested. Except for *BMY-3-snp2533*, the direction of the MFA effect was the same as in the CHIPS-ALL population (Table 5). Boxplots of the 12 most effective marker alleles show the effects of the allele’s presence/absence or increasing dosage on the tuber reducing sugar content after 12 weeks’ cold storage (Figure 3).

**Table 5:** Effects of markers associated with chip quality in the CHIPS-ALL population on reducing sugar content in the SUGAR40 panel after 0 (T0), 1 (T1), 2 (T2), 4 (T4), and 12 wk (T12) storage at 4° (log10 transformation, ANOVA)

Marker*^a^*	Minor Allele Frequency, %	T0 Percent Variance Explained*^b^*	T1 Percent Variance Explained*^b^*	T2 Percent Variance Explained*^b^*	T4 Percent Variance Explained*^b^*	T12 Percent Variance Explained*^b^*
***PWD-G_11140_A***	7.5 (*A*) ↑*^c^*	ns	9.6**	11.3**	19.2***	19.0***
*BMY1-G_2533_A*	29.4 (*A*) ↑	6.1*	7.0*	ns	5.2*	ns
*BMY1-G_2657_A*	7.0 (*A*) ↑	8.1**	5.4*	4.6*	4.7*	ns
*BMY1-G_2751_A*	6.4 (*A*) ↓	3.7*	5.3*	4.2*	3.7*	3.6*
***StpL(PHO1b)-3e_G_1305_T_1311_****^d^*	13.1 (1) ↑	18.0***	19.5***	16.9***	13.3***	15.6***
***StpL(PHO1b)-3b_A_158_C_159_ A_1250_****^d^*	9.4 (1) ↓	16.3***	25.4***	18.5***	21.1***	17.1***
***PHO1b-A_3931_G***	2.6 (*G*) ↓	12.6***	13.9***	13.4***	11.7***	13.0***
*PHO1b-A_3982_C*	25.6 (*C*) ↑↓	9.1*	9.8**	9.3*	10.3**	7.9*
*PHO1b-C_3990_T*	7.0 (*T*) ↑	6.2*	6.5*	11.2**	5.7*	ns
*PHO1b-G_4181_A*	16.7 (*A*) ↑↓	15.1***	14.2***	12.4**	11.6**	8.7**
***PHO1b-G_4106_A***	30.1 (*A*) ↑	18.9***	23.0***	25.7***	32.0***	25.9***
*PHO1b-A_4207_G*	16.0 (*G*) ↑	11.3*	14.6**	13.5**	ns	10.0*
*PHO1b-T_4404_C*	8.1 (*C*) ↑	8.9**	14.1***	16.3***	15.6***	8.8**
***Stp23-8b_PHO1a-H_A_****^d^*	6.2 (1) ↑	4.1*	5.8**	11.0***	9.3**	10.9***
***AGPaseS-T_1284_C***	5.6 (*C*) ↑	6.3**	4.1*	3.4*	7.2**	10.8***
***AGPaseS-A_1286_G***	35.2 (*G*) **↓**	16.8***	18.7***	16.0**	16.2**	16.6**
***INV-8/2-T_2076_C***	36.2 (*C*) **↓**	37.1***	42.6***	34.5***	34.0***	28.7***
***INV-8/2-T_2134_G***	35.0 (*G*) ↑	37.3***	40.0***	31.1***	33.8***	34.6***
*INV-8/2-T_2320_C*	11.2 (*C*) ↑**↓**	11.6**	8.4**	8.2**	7.4*	10.6**
*INV-8/2-G_2182_A*	16.2 (*A*) ↑	7.5*	5.6*	ns	ns	ns
***Pain1-8c_C_552_ A_718_****^d^*	5.0 (1) ↑	5.0*	3.9*	7.5**	10.9***	11.4***
*InvGE-6f_A_1103_**^d^*	6.2 (1) ↑	9.6**	7.8**	7.6**	12.1***	9.3**
***InvCD141-C_339_T* (*G_280_A*, *C_288_T*, *C_543_T*, *G_630_A****^e^*	13.7 (*T*) ↓	30.0***	30.6***	32.4***	37.1***	32.3***

ANOVA, analysis of variance; ns, not significant; SNP, single-nucleotide polymorphism; LD, linkage disequilibrium.

a Markers, for which box plots are shown in Figure 3 are in bold letters.

b Significance is indicated by “ns” not significant (*P* > 0.05), *0.05 > *P* ≥ 0.01, **0.01 > *P* ≥ 0.001 and ***P < 0.001.

c Arrows indicate the direction of the effect of the MFA, upwards for a positive (lower sugar content) and downward for a negative effect (higher sugar content); ↑↓: direction of effect was inconsistent between genotypic groups.

d Marker was scored by allele specific PCR assay, presence and absence of the MFA was scored as 1 and 0, respectively, without considering allele dosage.

e SNPs in parentheses were in strong LD, showed similar associations and the same direction of effect as the SNP, for which the data are shown.

### Genomic organization of genes functional in starch-sugar interconversion

**Figure 4:**
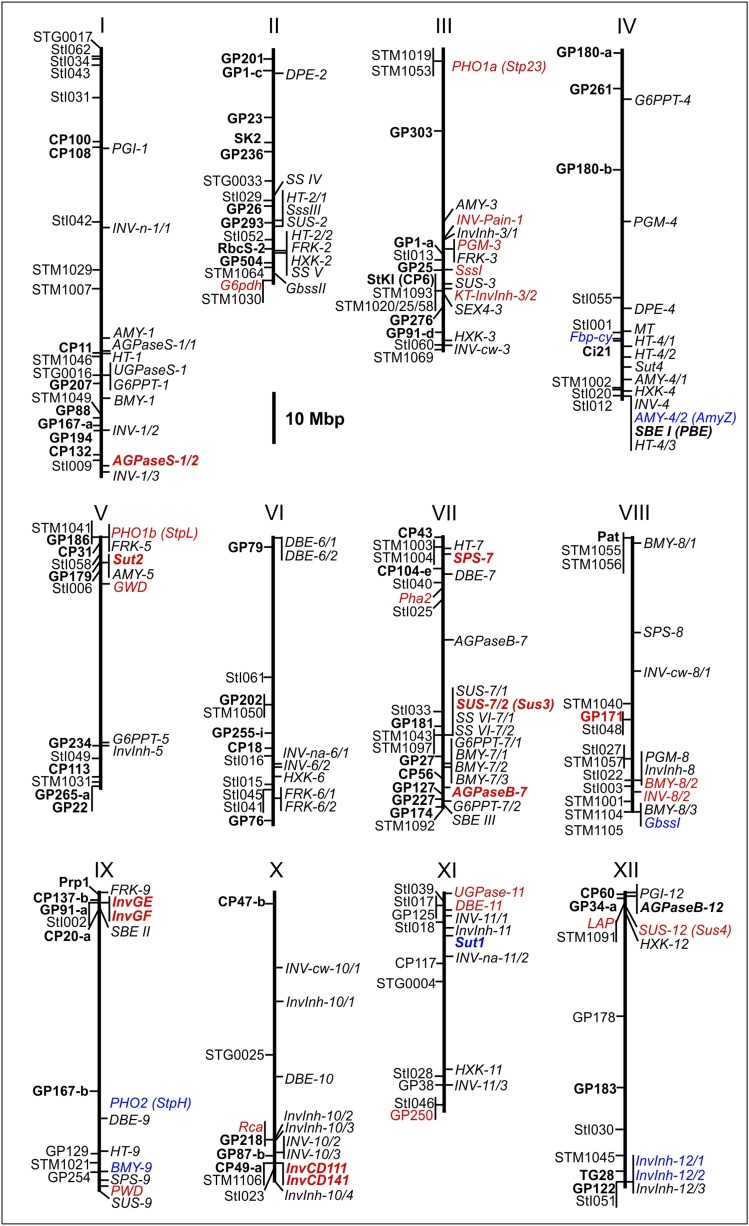
Physical map of candidate genes functional in starch-sugar interconversion. The 12 potato pseudomolecules (v4.03) (Sharma *et al.* 2013) are shown as solid vertical lines. The positions of 123 candidate loci specified in Table S1 are indicated to the right of the pseudomolecules (for acronyms, see also legend of Figure 1). Restriction fragment length polymorphism (GP***, CP***) (Menendez *et al.* 2002; Schäfer-Pregl *et al.* 1998) and microsatellite markers (STM****, StI***, STG****) (Feingold *et al.* 2005; Ghislain *et al.* 2009; Milbourne *et al.* 1998) anchoring potato genetic maps to the pseudomolecules are shown to the left. Candidate genes that have been tested for association (Fischer *et al.* 2013; Li *et al.* 2008) but are functional in pathways other than starch–sugar interconversion (Figure 1) also are shown to the left: G6PDH, glucose-6-phosphate dehydrogenase (EC 1.1.1.49); Fbp-cy, fructose-1,6-bisphosphatase (EC 3.1.3.11), cytosolic; Pha2, plasma membrane H^+^-ATPase 2 (EC 3.6.3.6); Rca, Ribulose bisphosphate carboxylase activase (EC 4.1.1.39); and LAP, leucine aminopeptidase (EC 3.4.11.1). Candidate genes and markers that were linked to QTL for tuber yield, starch, and/or reducing sugar content (Menendez *et al.* 2002; Schäfer-Pregl *et al.* 1998) are indicated in bold letters. Candidate genes and markers for which associations with tuber quality traits have been identified are shown in red letters, whereas candidate genes tested negatively for trait associations are shown in blue letters (Baldwin *et al.* 2011; Draffehn *et al.* 2010; Fischer *et al.* 2013; Kawchuk *et al.* 2008; Li *et al.* 2005, 2008, 2013; Urbany *et al.* 2011).

The annotated potato genome sequence (Potato Genome Sequencing Consortium *et al.* 2011) and improved physical maps of the 12 potato chromosomes (Sharma *et al.* 2013) allowed to estimate number and genomic positions of the genes, which function in starch-sugar interconversion according to the model shown in Figure 1. One hundred twenty-three expressed genes on all chromosomes were identified (Table S1 and Figure 4). A particularly high density of these genes was observed in distal regions of the long arms of chromosomes I, II, III, IV, and VII, where 50 genes (40%) were located. Except *GWD*, *PWD*, and *SEX4*, all enzymes and transporters are encoded by at least two genes, the largest family being putative invertases with 20 genes (Table S1). Approximately half of these genes have been cloned and characterized before in potato and/or tomato (Table S1). Seven of the previously characterized genes (5.7%) were not annotated in the current potato genome draft sequence. Including the results of this study, 25 loci functional in starch-sugar interconversion have been analyzed for association of DNA polymorphisms with tuber quality traits (Table S1) (Baldwin *et al.* 2011; Draffehn *et al.* 2010; Kawchuk *et al.* 2008; Li *et al.* 2005, 2008, 2013; Urbany *et al.* 2011), the majority of which showed associations of DNA polymorphisms with one or more tuber quality traits (Figure 4).

## Discussion

### Direct or indirect SNP-trait associations?

Except for the novel, functionally uncharacterized invertase *INV-8/2*, the candidate genes probed for association in this study were selected according to their known function in plastidial starch biosynthesis and degradation (Figure 1). Single SNPs or SNP haplotypes in all genes except *SssI* tagged two (*GWD*) to nine alleles (*PHO1b*) per locus, which were associated with either increased or decreased tuber starch content, starch yield, and/or chip quality in the CHIPS-ALL population. Effects on tuber yield were negligible. The high number of SNP-trait associations (49 of 176 SNPs scored in eight loci, significance threshold *P* < 0.01), particularly associations with TSC suggest that allelic DNA variation in at least some of the tested starch metabolizing genes contributes directly to the natural variation of tuber starch content, starch yield and, due to the inverse correlation between tuber starch and sugar content, chip quality. We cannot exclude, however, that the observed SNP-trait associations are indirect, resulting from LD with DNA variants in physically linked genes that are causal for the observed trait variation. At present, there is no genome-wide LD physical map available in potato, which would allow an estimate of the physical distances flanking the analyzed functional candidate loci, which should be considered for containing the causal gene(s). The recombination frequency in distal chromosomal regions is greater compared with pericentromeric regions (Sharma *et al.* 2013). LD blocks are therefore expected to be smaller in distal regions, where most candidate genes are located (Figure 4). The estimate of LD between the SNP markers scored in the CHIPS-ALL population revealed strong LD blocks only within but not between loci. The sizes of the detected LD blocks were limited by the size of the amplicon and could extend over distances larger than approximately 700 base pairs. Estimates for genome-wide average LD decay to r^2^ < 0.1 range from 275 base pairs (Stich *et al.* 2013) to 5 cM (D’hoop *et al.* 2010) corresponding to approximately 4 Mbp. For an individual genomic region, it has been shown that LD can extend across several hundred kilobase pairs (Achenbach *et al.* 2009). Expression patterns provide an auxiliary criterion for assessing the possibility of a causal role of a candidate gene. For example, *PHO1b* on the distal end of chromosome V is hardly expressed in tubers (Albrecht *et al.* 2001; Nader Nieto 2011), which makes it difficult to explain a direct effect of *PHO1b* allelic variation on tuber starch content and chip quality during tuber cold storage. The same argument speaks against a causal role of *INV-8/2*, which seems mostly expressed in flowers. The available expression data, however, do not exclude up-regulation of these genes in tubers in response to cold temperature (Claassen *et al.* 1993). Interestingly, 340 kbp proximal to *PHO1b* on the same superscaffold (PGSC0003DMB000000103) are two further candidate genes annotated, which are both expressed in tubers, a fructokinase (*FRK-5*; Figure 1 and Figure 4) and a 6-phosphogluconolactonase (6PGL, PGSC0003DMG400028363), the second enzyme in the pentose phosphate pathway (Kruger and von Schaewen 2003), which has been suggested to play a role in sugar metabolism during cold sweetening (Malone *et al.* 2006). Linkage mapping identified QTL for tuber starch content, yield, and reducing sugar content (Menendez *et al.* 2002; Schäfer-Pregl *et al.* 1998) in the distal, about 10 Mbp genomic region on the North arm of chromosome V, which includes besides *PHO1b*, *FRK-5*, and *6PGL* additional candidate genes (Figure 4). This finding suggests that more than one gene underlies these QTL. The same applies to other parts of the genome, where clusters of candidate genes overlap with previously mapped QTL such as the distal 10−20 Mbp regions of the South arms of chromosomes II, III, IV, VII, and X (Figure 4). High-resolution QTL linkage or association mapping is required to separate and eventually narrow down the genomic segments harboring the gene(s), which cause the observed effects on tuber quality traits.

### A molecular model for the association of L-type starch phosphorylase allele *PHO1a-H_A_* with increased tuber starch content

For the starch phosphorylase *PHO1a* we obtained evidence from functional characterization that this might indeed be one of the genes that cause natural variation of tuber starch and sugar content. The *PHO1a-H_A_* allele corresponding to the SSCP marker Stp23-8b was reproducibly associated with increased tuber starch content (Li *et al.* 2008, 2013). The sequence of the full-length PHO1a-H_A_ cDNA translated into a protein with threonine instead of a highly conserved alanine at position 108. This amino acid change is unique among all known starch and glycogen phosphorylases from plants, animals, and microorganisms. Protein modeling suggested that this mutation affects a conserved region important for dimerization (Watson *et al.* 1997) and decreases protein stability. It might also introduce an ectopic phosphorylation site (Rathore *et al.* 2009; Young *et al.* 2006). The functional PHO1 enzyme forms homodimers (*PHO1a-PHO1a*) as well as heterodimers (*PHO1a-PHO1b*) (Albrecht *et al.* 1998; Rathore *et al.* 2009). Complex formation and stability might be negatively affected by the Ala108Thr mutation, thereby reducing enzymatic activity. With increasing dosage of the *PHO1a-H_A_* allele in a tetraploid potato genotype, the proportion of PHO1 protein complexes containing PHO1a-H_A_ subunits would increase and the overall enzyme activity could therefore decrease. In fact, this was observed when comparing the enzyme activities in total tuber protein extracts from genotypes with none, one and two dosages of the *PHO1a-H_A_* allele (Figure 2), which supports the hypothesis that *PHO1a-H_A_* is an allele with additive effect. The consequence of reduced PHO1 activity could be impaired starch degradation and therefore a shift in the starch-sugar balance toward higher starch and lower sugar contents.

### Perspectives for breeding applications

Irrespective of whether or not a marker-trait association is direct or indirect, its diagnostic value increases with the reproducibility of the phenotypic effect in different genetic materials and environments. The effects on tuber starch content in the CHIPS-ALL population of two *PHO1* alleles (*PHO1a-H_A_* and *StpL(PHO1b)-3b*; Table 3) (Li *et al.* 2008) were independently also detected in the BRUISE population (Urbany *et al.* 2011). This was concluded from the correspondence between *PHO1* markers evaluated in both populations. However, due to the correlation between tuber starch content and susceptibility to bruising, for a positive allele effect on tuber starch content there is a penalty by higher bruising susceptibility and vice versa. As this correlation is not absolute and additional candidate gene markers for starch corrected resistance to bruising have been identified (Urbany *et al.* 2011), marker-assisted selection of genotypes combining optimal starch with low sugar content and acceptable susceptibility to bruising should be feasible. The effects of most candidate gene alleles on chip quality in the CHIPS-ALL population were reproducible, despite its small size, in the SUGAR40 panel, which has been phenotyped for reducing sugar content (Fischer *et al.* 2013) (Table 5 and Figure 3). Most interesting for optimizing processing quality are low frequency SNP alleles or haplotypes that are diagnostic for better processing quality, for example *PWD-A_11140_*, *AGPaseS-C_1284_*, *PHO1b-G_1305_T_1311_*, *Pain1-C_552_A_718_*, *PHO1a-T_22_A_322_G_824_A_2776_* (*PHO1a-H_A_*) and *InvGE-A_1103_* (Table 5). Increasing the frequency of these alleles in breeding populations is expected to improve the average processing quality. A positive effect may also be achieved by selecting against frequent alleles which reduce processing quality, for example *Inv-8/2-G_2065_C_2076_A_2272_A _2116_* and *InvCD141-A_280_T_288_T_339_T_543_A_630_C_1030_G_1031_T_1096_* (Table 5). Marker-assisted breeding for complex tuber quality traits might also have to consider intra and inter locus interactions (Li *et al.* 2010, 2013). Comprehensive approaches such as genomic selection take into account genome wide markers simultaneously (Jonas and de Koning 2013; Nakaya and Isobe 2012). The limited number of SNP markers diagnostic for tuber quality traits described in this and previous papers (Draffehn *et al.* 2010; Fischer *et al.* 2013; Li *et al.* 2013) can serve as a marker toolkit of low complexity for exploring the feasibility of genomic selection in the polyploid, noninbred potato crop (Nakaya and Isobe 2012).

### Genetic complexity of natural variation of tuber quality

Searching the potato genome for genes functional in starch-sugar interconversion (Figure 1) based on *in silico* annotation on the one hand, and on the other hand searching the NCBI database for genuine genes of the same type, which have been cloned and characterized in potato and/or tomato, resulted in 123 genes, of which about half have been characterized previously (Table S1). Even if some of these 123 genes are annotation artifacts, pseudogenes or genes with a different *in vivo* function than suggested by *in silico* annotation, this number is much larger than the number of loci that have been actually evaluated for DNA polymorphisms and their associations with tuber quality traits. Furthermore, the metabolic scheme in Figure 1 excludes deliberately other processes and pathways, which also play a role in starch accumulation in maturing and starch-sugar equilibrium in dormant tubers, such as glycolysis, the oxidative pentose phosphate pathway, mitochondrial respiration and ATP supply, efficiency of photosynthesis, CO_2_ fixation and carbon partitioning between source and sink tissues (Frommer and Sonnewald 1995; Kruger and von Schaewen 2003; Malone *et al.* 2006; Sowokinos 2001). Except inhibition of invertase by proteinaceous inhibitors, regulatory and signaling processes such as protein phosphorylation, dephosphorylation, redox regulation, and sugar sensing (Geigenberger *et al.* 2005; Halford and Paul 2003; Tetlow *et al.* 2004; Tiessen *et al.* 2002) also have not been considered. Including all genes known to affect, or potentially affecting directly or indirectly the starch-sugar balance in potato tubers will inflate the number of candidates to several hundred, which renders it impractical to test one by one based on the candidate gene association mapping approach. To capture comprehensively the DNA variation underlying these complex tuber quality traits, genotyping populations by next-generation sequencing provides new possibilities (Elshire *et al.* 2011; Sonah *et al.* 2013; Spindel *et al.* 2013).

Genetic dissection of tuber quality traits by genome-wide QTL linkage and association mapping based on restriction fragment length polymorphism and amplified fragment length polymorphism markers identified in the order of 15 to 25 QTL for tuber starch content and processing quality (D’hoop *et al.* 2014; Menendez *et al.* 2002; Schäfer-Pregl *et al.* 1998). Due to lack of genetic resolution, lack of common markers in the different mapping studies and lack of anchors between the genetic map in the association study of D’hoop *et al.* (2014) and the potato genome sequence, it is neither possible to estimate more precisely the numbers of QTL nor to correlate most of the QTL identified in (D’hoop *et al.* 2014) with the physical map of QTL and candidate genes in this study (Figure 4). Nevertheless, the number of so far known QTL for tuber quality traits related to starch-sugar interconversion appears at least 10-fold less than the anticipated number of functional candidate genes. Even if more than one locus is responsible for each of these QTL, this suggests that only a subset of all genes functional in starch-sugar interconversion exert substantial control on the natural variation of tuber starch and sugar content. Rather than the total number of loci involved, multiple alleles with different phenotypic effects, as observed for most of the associated candidate loci, might be responsible for the genetic complexity of tuber starch and sugar content.

## Supplementary Material

Supporting Information
