# Supplementary material for: SNPs in Genes Functional in Starch-Sugar Interconversion Associate with Natural Variation of Tuber Starch and Sugar Content of Potato (Solanum tuberosum L.)
Source: G3 (Bethesda). 2014 Jul 31;4(10):1797–811. doi: 10.1534/g3.114.012377 (PMC4199688; doi:10.1534/g3.114.012377)
Supplement: Supporting Information [file supp_4_10_1797__index.html]

SNPs in Genes Functional in Starch-Sugar Interconversion Associate with Natural Variation of Tuber Starch and Sugar Content of Potato (Solanum tuberosum L.) — Supporting Information 

# SNPs in Genes Functional in Starch-Sugar Interconversion Associate with Natural Variation of Tuber Starch and Sugar Content of Potato (*Solanum tuberosum* L.)

## Supporting Information for Schreiber *et al.*, 2014

**Files in this Data Supplement:**

- Supporting Information - Figures S1-S2, Files S1-S2, and Tables S1-S2 (PDF, 727 KB)
- Figure S1 - Sequence alignment of nine Pho1a cDNA alleles. (PDF, 462 KB)
- Figure S2 - Alignment of plant PHO1a deduced protein sequences. (PDF, 349 KB)
- File S1 - Amplified potato genomic fragments including SNP positions, nucleotide alleles and identifications. (PDF, 165 KB)
- Table S1 - Genes functional in plant starch-sugar interconversion as annotated in the potato genome and GeneBank accessions of corresponding cloned and characterized genes from potato and/or tomato. (PDF, 137 KB)
- Table S2 - Correspondence between presence (1) or absence (0) of the Stp23-8b SSCP marker and PHO1a cDNA SNPs in 34 standard varieties of the CHIPS-ALL population (Li *et al.* 2008). (PDF, 164 KB)
- File S2 - Pair wise linkage disequilibrium (LD) between SNP markers scored in the CHIPS-ALL population. (.xlsx, 685 KB)
