# Supplementary material for: SNPs in Genes Functional in Starch-Sugar Interconversion Associate with Natural Variation of Tuber Starch and Sugar Content of Potato (Solanum tuberosum L.)
Source: G3 (Bethesda). 2014 Jul 31;4(10):1797–811. doi: 10.1534/g3.114.012377 (PMC4199688; doi:10.1534/g3.114.012377)
Supplement: Supporting Information [file supp_g3.114.012377_012377SI.pdf]

G3· Genes | Genomes | Genetics

SNPs in genes functional in starch-sugar interconversion associate with natural variation of tuber starch and sugar content of potato (*Solanum tuberosum* L).

L. Schreiber *, A. C. Nader-Nieto *, E. M. Schönhals, B. Walkemeier, C. Gebhardt
Max Planck Institute for Plant Breeding Research, Department of Plant Breeding and Genetics, 50829 Cologne, Germany
*The two first authors equally contributed to this work

Corresponding author:
Christiane Gebhardt Max Planck Institute for Plant Breeding Research
Carl-von-Linné-Weg 10, 50829 Cologne, Germany
Phone: +49 (0)221 5062 430
Email: (gebhardt@mpipz.mpg.de)

DOI: 10.1534/g3.114.012377

```
                     *        20         *        40         *        60         *        80         *       100
Pho1a-HR : ATGGCGACTGCAAATGGAGCACACTTGTTCAACCATTACAGCTCCAATTCCAGATTCATCCATTTCACTTCTAGAAACACAAGCTCCAAATTGTTCCTTA :  100
Pho1a(2) : ATGGCGACTGCAAATGGAGCATACTTGTTCAACCATTACAGCTCCAATTCCAGATTCATCCATTTCACTTCTAGAAACACAAGCTCCAAATTGTTCCTTA :  100
Pho1a(3) : ATGGCGACTGCAAATGGAGCATACTTGTTCAACCATTACAGCTCCAATTCCAGATTCATCCATTTCACTTCTAGAAACACAAGCTCCAAATTGTTCCTTA :  100
Pho1a(4) : ATGGCGACTGCAAATGGAGCATACTTGTTCAACCATTACAGCTCCAATTCCAGATTCATCCATTTCACTTCTAGAAACACAAGCTCCAAATTGTTCCTTA :  100
Pho1a-HA : ATGGCGACTGCAAATGGAGCATACTTGTTCAACCATTACAGCTCCAATTCCAGATTCATCCATTTCACTTCTAGAAACACAAGCTCCAAATTGTTCCTTA :  100
Pho1a(6) : ATGGCGACTGCAAATGGAGCATACTTGTTCAACCATTACAGCTCCAATTCCAGATTCATCCATTTCACTTCTAGAAACACAAGCTCCAAATTGTTCCTTA :  100
Pho1a(7) : ATGGCGACTGCAAATGGAGCACACTTGTTCAACCATTACAGCTCCAATTCCAGATTCATCCATTTCACTTCTAGAAACACAAGCTCCAAATTGTTCCTTA :  100
Pho1a(8) : ATGGCGACTGCAAATGGAGCACACTTGTTCAACCATTACAGCTCCAATTCCAGATTCATCCATTTCACTTCTAGAAACACAAGCTCCAAATTGTTCCTTA :  100
Pho1a(9) : ATGGCGACTGCAAATGGAGCACACTTGTTCAACCATTACAGCTCCAATTCCAGATTCATCCATTTCACTTCTAGAAACACAAGCTCCAAATTGTTCCTTA :  100

                     *       120         *       140         *       160         *       180         *       200
Pho1a-HR : CCAAAACCTCCCATTTTCGGAGACCCAAACGCTGTTTCCATGTCAACAATACCTTGAGTGAGAAAATTCACCATCCCATTACTGAACAAGGTGGTGAGAG :  200
Pho1a(2) : CCAAAACCTCCCATTTTCGGAGACCCAAACGCTGTTTCCATGTCAACAATACCTTGAGTGAGAAAATTCACCATCCCATTACTGAACAAGGTGGTGAGAG :  200
Pho1a(3) : CCAAAACCTCCCATTTTCGGAGACCCAAACGCTGTTTCCATGTCAACAATACCTTGAGTGAGAAAATTCACCATCCCATTACTGAACAAGGTGGTGAGAG :  200
Pho1a(4) : CCAAAACCTCCCATTTTCGGAGACCCAAACGCTGTTTCCATGTCAACAATACCTTGAGTGAGAAAATTCACCATCCCATTACTGAACAAGGTGGTGAGAG :  200
Pho1a-HA : CCAAAACCTCCCATTTTCGGAGACCCAAACGCTGTTTCCATGTCAACAATACCTTGAGTGAGAAAATTCACCATCCCATTACTGAACAAGGTGGTGAGAG :  200
Pho1a(6) : CCAAAACCTCCCATTTTCGGAGACCCAAACGCTGTTTCCATGTCAACAATACCTTGAGTGAGAAAATTCACCATCCCATTACTGAACAAGGTGGTGAGAG :  200
Pho1a(7) : CCAAAACCTCCCATTTTCGGAGACCCAAACGCTGTTTCCATGTCAACAATACCTTGAGTGAGAAAATTCACCATCCCATTACTGAACAAGGTGGTGAGAG :  200
Pho1a(8) : CCAAAACCTCCCATTTTCGGAGACCCAAACGCTGTTTCCATGTCAACAATACCTTGAGTGAGAAAATTCACCATCCCATTACTGAACAAGGTGGTGAGAG :  200
Pho1a(9) : CCAAAACCTCCCATTTTCGGAGACCCAAACGCTGTTTCCATGTCAACAATACCTTGAGTGAGAAAATTCACCATCCCATTACTGAACAAGGTGGTGAGAG :  200

                     *       220         *       240         *       260         *       280         *       300
Pho1a-HR : CGACCTGAGTTCTTTTGCTCCTGATGCCGCATCTATTACCTCAAGTATCAAATACCATGCAGAATTCACACCTGTATTCTCTCCTGAAAGGTTTGAGCTC :  300
Pho1a(2) : CGACCTGAGTTCTTTTGCTCCTGATGCCGCATCTATTACCTCAAGTATCAAATACCATGCAGAATTCACACCTGTATTCTCTCCTGAAAGGTTTGAGCTC :  300
Pho1a(3) : CGACCTGAGTTCTTTTGCTCCTGATGCCGCATCTATTACCTCAAGTATCAAATACCATGCAGAATTCACACCTGTATTCTCTCCTGAAAGGTTTGAGCTC :  300
Pho1a(4) : CGACCTGAGTTCTTTTGCTCCTGATGCCGCATCTATTACCTCAAGTATCAAATACCATGCAGAATTCACACCTGTATTCTCTCCTGAAAGGTTTGAGCTC :  300
Pho1a-HA : CGACCTGAGTTCTTTTGCTCCTGATGCCGCATCTATTACCTCAAGTATCAAATACCATGCAGAATTCACACCTGTATTCTCTCCTGAAAGGTTTGAGCTC :  300
Pho1a(6) : CGACCTGAGTTCTTTTGCTCCTGATGCCGCATCTATTACCTCAAGTATCAAATACCATGCAGAATTCACACCTGTATTCTCTCCTGAAAGGTTTGAGCTC :  300
Pho1a(7) : CGACCTGAGTTCTTTTGCTCCTGATGCCGCATCTATTACCTCAAGTATCAAATACCATGCAGAATTCACACCTGTATTCTCTCCTGAAAGGTTTGAGCTC :  300
Pho1a(8) : CGACCTGAGTTCTTTTGCTCCTGATGCCGCATCTATTACCTCAAGTATCAAATACCATGCAGAATTCACACCTGTATTCTCTCCTGAAAGGTTTGAGCTC :  300
Pho1a(9) : CGACCTGAGTTCTTTTGCTCCTGATGCCGCATCTATTACCTCAAGTATCAAATACCATGCAGAATTCACACCTGTATTCTCTCCTGAAAGGTTTGAGCTC :  300

                     *       320         *       340         *       360         *       380         *       400
Pho1a-HR : CCTAAGGCATTCTTTGCAACAGCTCAAAGTGTTCGTGATTCGCTCCTTATTAATTGGAATGCTACGTATGATATTTATGAAAAGCTGAACATGAAGCAAG :  400
Pho1a(2) : CCTAAGGCATTCTTTGCAACAACTCAAAGTGTTCGTGATTCGCTCCTTATTAATTGGAATGCTACGTATGATATTTATGAAAAGCTGAACATGAAGCAAG :  400
Pho1a(3) : CCTAAGGCATTCTTTGCAACAACTCAAAGTGTTCGTGATTCGCTCCTTATTAATTGGAATGCTACGTATGATATTTATGAAAAGCTGAACATGAAGCAAG :  400
Pho1a(4) : CCTAAGGCATTCTTTGCAACAACTCAAAGTGTTCGTGATTCGCTCCTTATTAATTGGAATGCTACGTATGATATTTATGAAAAGCTGAACATGAAGCAAG :  400
Pho1a-HA : CCTAAGGCATTCTTTGCAACAACTCAAAGTGTTCGTGATTCGCTCCTTATTAATTGGAATGCTACGTATGATATTTATGAAAAGCTGAACATGAAGCAAG :  400
Pho1a(6) : CCTAAGGCATTCTTTGCAACAACTCAAAGTGTTCGTGATTCGCTCCTTATTAATTGGAATGCTACGTATGATATTTATGAAAAGCTGAACATGAAGCAAG :  400
Pho1a(7) : CCTAAGGCATTCTTTGCAACAGCTCAAAGTGTTCGTGATTCGCTCCTTATTAATTGGAATGCTACGTATGATATTTATGAAAAGCTGAACATGAAGCAAG :  400
Pho1a(8) : CCTAAGGCATTCTTTGCAACAGCTCAAAGTGTTCGTGATTCGCTCCTTATTAATTGGAATGCTACGTATGATATTTATGAAAAGCTGAACATGAAGCAAG :  400
Pho1a(9) : CCTAAGGCATTCTTTGCAACAGCTCAAAGTGTTCGTGATTCGCTCCTTATTAATTGGAATGCTACGTATGATATTTATGAAAAGCTGAACATGAAGCAAG :  400

                     *       420         *       440         *       460         *       480         *       500
Pho1a-HR : CGTACTATCTATCCATGGAATTTCTGCAGGGTAGAGCATTGTTAAATGCAATTGGTAATCTGGAGCTTACTGGTGCATTTGCGGAAGCTTTGAAAAACCT :  500
Pho1a(2) : CGTACTATCTATCCATGGAATTTCTGCAGGGTAGAGCATTGTTAAATGCAATTGGTAATCTGGAGCTTACTGGTGCATTTGCGGAAGCTTTGAAAAACCT :  500
Pho1a(3) : CGTACTATCTATCCATGGAATTTCTGCAGGGTAGAGCATTGTTAAATGCAATTGGTAATCTGGAGCTTACTGGTGCATTTGCGGAAGCTTTGAAAAACCT :  500
Pho1a(4) : CGTACTATCTATCCATGGAATTTCTGCAGGGTAGAGCATTGTTAAATGCAATTGGTAATCTGGAGCTTACTGGTGCATTTGCGGAAGCTTTGAAAAACCT :  500
Pho1a-HA : CGTACTATCTATCCATGGAATTTCTGCAGGGTAGAGCATTGTTAAATGCAATTGGTAATCTGGAGCTTACTGGTGCATTTGCGGAAGCTTTGAAAAACCT :  500
Pho1a(6) : CGTACTATCTATCCATGGAATTTCTGCAGGGTAGAGCATTGTTAAATGCAATTGGTAATCTGGAGCTTACTGGTGCATTTGCGGAAGCTTTGAAAAACCT :  500
Pho1a(7) : CGTACTATCTATCCATGGAATTTCTGCAGGGTAGAGCATTGTTAAATGCAATTGGTAATCTGGAGCTTACTGGTGCATTTGCGGAAGCTTTGAAAAACCT :  500
Pho1a(8) : CGTACTATCTATCCATGGAATTTCTGCAGGGTAGAGCATTGTTAAATGCAATTGGTAATCTGGAGCTTACTGGTGCATTTGCGGAAGCTTTGAAAAACCT :  500
Pho1a(9) : CGTACTATCTATCCATGGAATTTCTGCAGGGTAGAGCATTGTTAAATGCAATTGGTAATCTGGAGCTTACTGGTGCATTTGCGGAAGCTTTGAAAAACCT :  500

                     *       520         *       540         *       560         *       580         *       600
Pho1a-HR : TGGCCACAATCTAGAAAATGTGGCTTCTCAGGAACCAGATGCTGCTCTTGGAAATGGGGGTTTGGGACGGCTTGCTTCCTGTTTTCTGGACTCTTTGGCA :  600
Pho1a(2) : TGGCCACAATCTAGAAAATGTGGCTTCTCAGGAGCCAGATGCTGCTCTTGGAAATGGGGGTTTGGGACGGCTTGCTTCCTGTTTTCTGGACTCTTTGGCA :  600
Pho1a(3) : TGGCCACAATCTAGAAAATGTGGCTTCTCAGGAGCCAGATGCTGCTCTTGGAAATGGGGGTTTGGGACGGCTTGCTTCCTGTTTTCTGGACTCTTTGGCA :  600
Pho1a(4) : TGGCCACAATCTAGAAAATGTGGCTTCTCAGGAGCCAGATGCTGCTCTTGGAAATGGGGGTTTGGGACGGCTTGCTTCCTGTTTTCTGGACTCTTTGGCA :  600
Pho1a-HA : TGGCCACAATCTAGAAAATGTGGCTTCTCAGGAGCCAGATGCTGCTCTTGGAAATGGGGGTTTGGGACGGCTTGCTTCCTGTTTTCTGGACTCTTTGGCA :  600
Pho1a(6) : TGGCCACAATCTAGAAAATGTGGCTTCTCAGGAGCCAGATGCTGCTCTTGGAAATGGGGGTTTGGGACGGCTTGCTTCCTGTTTTCTGGACTCTTTGGCA :  600
Pho1a(7) : TGGTCACAATCTAGAAAATGTGGCTTCTCAGGAGCCAGATGCTGCTCTTGGAAATGGGGGTTTGGGACGGCTTGCTTCCTGTTTTCTGGACTCTTTGGCA :  600
Pho1a(8) : TGGTCACAATCTAGAAAATGTGGCTTCTCAGGAGCCAGATGCTGCTCTTGGAAATGGGGGTTTGGGACGGCTTGCTTCCTGTTTTCTGGACTCTTTGGCA :  600
Pho1a(9) : TGGTCACAATCTAGAAAATGTGGCTTCTCAGGAGCCAGATGCTGCTCTTGGAAATGGGGGTTTGGGACGGCTTGCTTCCTGTTTTCTGGACTCTTTGGCA :  600

                     *       620         *       640         *       660         *       680         *       700
Pho1a-HR : ACACTAAACTACCCAGCATGGGGCTATGGACTTAGGTACAAGTATGGTTTATTTAAGCAACGGATTACAAAAGATGGTCAGGAGGAGGTGGCTGAAGATT :  700
Pho1a(2) : ACACTAAACTACCCAGCATGGGGCTATGGACTTAGGTACAAGTATGGTTTATTTAAGCAACGGATTACAAAAGATGGTCAGGAGGAGGTGGCTGAAGATT :  700
Pho1a(3) : ACACTAAACTACCCAGCATGGGGCTATGGACTTAGGTACAAGTATGGTTTATTTAAGCAACGGATTACAAAAGATGGTCAGGAGGAGGTGGCTGAAGATT :  700
Pho1a(4) : ACACTAAACTACCCAGCATGGGGCTATGGACTTAGGTACAAGTATGGTTTATTTAAGCAACGGATTACAAAAGATGGTCAGGAGGAGGTGGCTGAAGATT :  700
Pho1a-HA : ACACTAAACTACCCAGCATGGGGCTATGGACTTAGGTACAAGTATGGTTTATTTAAGCAACGGATTACAAAAGATGGTCAGGAGGAGGTGGCTGAAGATT :  700
Pho1a(6) : ACACTAAACTACCCAGCATGGGGCTATGGACTTAGGTACAAGTATGGTTTATTTAAGCAACGGATTACAAAAGATGGTCAGGAGGAGGTGGCTGAAGATT :  700
Pho1a(7) : ACACTAAACTACCCAGCATGGGGCTATGGACTTAGGTACAAGTATGGTTTATTTAAGCAACGGATTACAAAAGATGGTCAGGAGGAGGTGGCTGAAGATT :  700
Pho1a(8) : ACACTAAACTACCCAGCATGGGGCTATGGACTTAGGTACAAGTATGGTTTATTTAAGCAACGGATTACAAAAGATGGTCAGGAGGAGGTGGCTGAAGATT :  700
Pho1a(9) : ACACTAAACTACCCAGCATGGGGCTATGGACTTAGGTACAAGTATGGTTTATTTAAGCAACGGATTACAAAAGATGGTCAGGAGGAGGTGGCTGAAGATT :  700

                     *       720         *       740         *       760         *       780         *       800
Pho1a-HR : GGCTTGAAATTGGCAGTCCATGGGAAGTTGTGAGGAATGATGTTTCATATCCTATCAAATTCTATGGAAAAGTCTCTACAGGATCAGATGGAAAGAGGTA :  800
Pho1a(2) : GGCTTGAAATTGGCAGTCCCTGGGAAGTTGTGAGGAATGATGTTTCATATCCTATCAAATTCTATGGAAAAGTCTCTACAGGATCAGATGGAAAGAGGTA :  800
Pho1a(3) : GGCTTGAAATTGGCAGTCCCTGGGAAGTTGTGAGGAATGATGTTTCATATCCTATCAAATTCTATGGAAAAGTCTCTACAGGATCAGATGGAAAGAGGTA :  800
Pho1a(4) : GGCTTGAAATTGGCAGTCCCTGGGAAGTTGTGAGGAATGATGTTTCATATCCTATCAAATTCTATGGAAAAGTCTCTACAGGATCAGATGGAAAGAGGTA :  800
Pho1a-HA : GGCTTGAAATTGGCAGTCCCTGGGAAGTTGTGAGGAATGATGTTTCATATCCTATCAAATTCTATGGAAAAGTCTCTACAGGATCAGATGGAAAGAGGTA :  800
Pho1a(6) : GGCTTGAAATTGGCAGTCCCTGGGAAGTTGTGAGGAATGATGTTTCATATCCTATCAAATTCTATGGAAAAGTCTCTACAGGATCAGATGGAAAGAGGTA :  800
Pho1a(7) : GGCTTGAAATTGGCAGTCCATGGGAAGTTGTGAGGAATGATGTTTCATATCCTATCAAATTCTATGGAAAAGTCTCTACAGGATCAGATGGAAAGAGGTA :  800
Pho1a(8) : GGCTTGAAATTGGCAGTCCATGGGAAGTTGTGAGGAATGATGTTTCATATCCTATCAAATTCTATGGAAAAGTCTCTACAGGATCAGATGGAAAGAGGTA :  800
Pho1a(9) : GGCTTGAAATTGGCAGTCCATGGGAAGTTGTGAGGAATGATGTTTCATATCCTATCAAATTCTATGGAAAAGTCTCTACAGGATCAGATGGAAAGAGGTA :  800
```

```
                        *        820          *        840          *        860          *        880          *        900
Pho1a-HR : TTGGATTGGTGGAGAGGATATAAAGGCAGTTGCGTATGATGTTCCCATACCAGGGTATAAGACCAGAACCACAATCAGCCTTCGACTGTGGTCTACACAG :  900
Pho1a(2) : TTGGATTGGTGGAGAGGATATAAAGGCAGTTGCGTATGATGTTCCCATACCAGGGTATAAGACCAGAACCACAATCAGCCTTCGACTGTGGTCTACACAG :  900
Pho1a(3) : TTGGATTGGTGGAGAGGATATAAAGGCAGTTGCGTATGATGTTCCCATACCAGGGTATAAGACCAGAACCACAATCAGCCTTCGACTGTGGTCTACACAG :  900
Pho1a(4) : TTGGATTGGTGGAGAGGATATAAGGGCAGTTGCGTATGATGTTCCCATACCAGGGTATAAGACCAGAACCACAATCAGCCTTCGACTGTGGTCTACACAG :  900
Pho1a-HA : TTGGATTGGTGGAGAGGATATAAGGGCAGTTGCGTATGATGTTCCCATACCAGGGTATAAGACCAGAACCACAATCAGCCTTCGACTGTGGTCTACACAG :  900
Pho1a(6) : TTGGATTGGTGGAGAGGATATAAGGGCAGTTGCGTATGATGTTCCCATACCAGGGTATAAGACCAGAACCACAATCAGCCTTCGACTGTGGTCTACACAG :  900
Pho1a(7) : TTGGATTGGTGGAGAGGATATAAAGGCAGTTGCGTATGATGTTCCCATACCAGGGTATAAGACCAGAACCACAATCAGCCTTCGACTGTGGTCTACACAG :  900
Pho1a(8) : TTGGATTGGTGGAGAGGATATAAAGGCAGTTGCGTATGATGTTCCCATACCAGGGTATAAGACCAGAACCACAATCAGCCTTCGACTGTGGTCTACACAG :  900
Pho1a(9) : TTGGATTGGTGGAGAGGATATAAAGGCAGTTGCGTATGATGTTCCCATACCAGGGTATAAGACCAGAACCACAATCAGCCTTCGACTGTGGTCTACACAG :  900

                        *        920          *        940          *        960          *        980          *       1000
Pho1a-HR : GTTCCATCAGCGGATTTTGATTTATCTGCTTTCAATGCTGGAGAGCACACCAAAGCATGTGAAGCCCAAGCAAACGCTGAGAAGATATGTTACATACTCT : 1000
Pho1a(2) : GTTCCATCAGCGGATTTTGATTTATCTGCTTTCAATGCTGGAGAGCACACCAAAGCATGTGAAGCCCAAGCAAACGCTGAGAAGATATGTTACATACTCT : 1000
Pho1a(3) : GTTCCATCAGCGGATTTTGATTTATCTGCTTTCAATGCTGGAGAGCACACCAAAGCATGTGAAGCCCAAGCAAACGCTGAGAAGATATGTTACATACTCT : 1000
Pho1a(4) : GTTCCATCAGCGGATTTTGATTTATCTGCTTTCAATGCTGGAGAGCACACCAAAGCATGTGAAGCCCAAGCAAACGCTGAGAAGATATGTTACATACTCT : 1000
Pho1a-HA : GTTCCATCAGCGGATTTTGATTTATCTGCTTTCAATGCTGGAGAGCACACCAAAGCATGTGAAGCCCAAGCAAACGCTGAGAAGATATGTTACATACTCT : 1000
Pho1a(6) : GTTCCATCAGCGGATTTTGATTTATCTGCTTTCAATGCTGGAGAGCACACCAAAGCATGTGAAGCCCAAGCAAACGCTGAGAAGATATGTTACATACTCT : 1000
Pho1a(7) : GTTCCATCAGCGGATTTTGATTTATCTGCTTTCAATGCTGGAGAGCACACCAAAGCATGTGAAGCCCAAGCAAACGCTGAGAAGATATGTTACATACTCT : 1000
Pho1a(8) : GTTCCATCAGCGGATTTTGATTTATCTGCTTTCAATGCTGGAGAGCACACCAAAGCATGTGAAGCCCAAGCAAACGCTGAGAAGATATGTTACATACTCT : 1000
Pho1a(9) : GTTCCATCAGCGGATTTTGATTTATCTGCTTTCAATGCTGGAGAGCACACCAAAGCATGTGAAGCCCAAGCAAACGCTGAGAAGATATGTTACATACTCT : 1000

                        *       1020          *       1040          *       1060          *       1080          *       1100
Pho1a-HR : ACCCTGGGGATGAATCAGAGGAGGGAAAGATCCTTCGGTTGAAGCAACAATATACCTTATGCTCGGCTTCTCTCCAAGATATTATTTCTCGATTTGAGAG : 1100
Pho1a(2) : ACCCTGGGGATGAATCAGAGGAGGGAAAGATCCTTCGGTTGAAGCAACAATATACCTTATGCTCGGCTTCTCTCCAAGATATTATTTCTCGATTTGAGAG : 1100
Pho1a(3) : ACCCTGGGGATGAATCAGAGGAGGGAAAGATCCTTCGGTTGAAGCAACAATATACCTTATGCTCGGCTTCTCTCCAAGATATTATTTCTCGATTTGAGAG : 1100
Pho1a(4) : ACCCTGGGGATGAATCAGAGGAGGGAAAGATCCTTCGGTTGAAGCAACAATATACCTTATGCTCGGCTTCTCTCCAAGATATTATTTCTCGATTTGAGAG : 1100
Pho1a-HA : ACCCTGGGGATGAATCAGAGGAGGGAAAGATCCTTCGGTTGAAGCAACAATATACCTTATGCTCGGCTTCTCTCCAAGATATTATTTCTCGATTTGAGAG : 1100
Pho1a(6) : ACCCTGGGGATGAATCAGAGGAGGGAAAGATCCTTCGGTTGAAGCAACAATATACCTTATGCTCGGCTTCTCTCCAAGATATTATTTCTCGATTTGAGAG : 1100
Pho1a(7) : ACCCTGGGGATGAATCAGAGGAGGGAAAGATCCTTCGGTTGAAGCAACAATATACCTTATGCTCGGCTTCTCTCCAAGATATTATTTCTCGATTTGAGAG : 1100
Pho1a(8) : ACCCTGGGGATGAATCAGAGGAGGGAAAGATCCTTCGGTTGAAGCAACAATATACCTTATGCTCGGCTTCTCTCCAAGATATTATTTCTCGATTTGAGAG : 1100
Pho1a(9) : ACCCTGGGGATGAATCAGAGGAGGGAAAGATCCTTCGGTTGAAGCAACAATATACCTTATGCTCGGCTTCTCTCCAAGATATTATTTCTCGATTTGAGAG : 1100

                        *       1120          *       1140          *       1160          *       1180          *       1200
Pho1a-HR : GAGATCAGGTGATCGTATTAAGTGGGAAGAGTTTCCTGAAAAAGTTGCTGTGCAGATGAATGACACTCACCCTACACTTTGTATCCCTGAGCTGATGAGA : 1200
Pho1a(2) : GAGATCAGGTGATCGTATTAAGTGGGAAGAGTTTCCTGAAAAAGTTGCTGTGCAGATGAATGACACTCACCCTACACTTTGTATCCCTGAGCTGATGAGA : 1200
Pho1a(3) : GAGATCAGGTGATCGTATTAAGTGGGAAGAGTTTCCTGAAAAAGTTGCTGTGCAGATGAATGACACTCACCCTACACTTTGTATCCCTGAGCTGATGAGA : 1200
Pho1a(4) : GAGATCAGGTGATCGTATTAAGTGGGAAGAGTTTCCTGAAAAAGTTGCTGTGCAGATGAATGACACTCACCCTACACTTTGTATCCCTGAGCTGATGAGA : 1200
Pho1a-HA : GAGATCAGGTGATCGTATTAAGTGGGAAGAGTTTCCTGAAAAAGTTGCTGTGCAGATGAATGACACTCACCCTACACTTTGTATCCCTGAGCTGATGAGA : 1200
Pho1a(6) : GAGATCAGGTGATCGTATTAAGTGGGAAGAGTTTCCTGAAAAAGTTGCTGTGCAGATGAATGACACTCACCCTACACTTTGTATCCCTGAGCTGATGAGA : 1200
Pho1a(7) : GAGATCAGGTGATCGTATTAAGTGGGAAGAGTTTCCTGAAAAAGTTGCTGTGCAGATGAATGACACTCACCCTACACTTTGTATCCCTGAGCTGATGAGA : 1200
Pho1a(8) : GAGATCAGGTGATCGTATTAAGTGGGAAGAGTTTCCTGAAAAAGTTGCTGTGCAGATGAATGACACTCACCCTACACTTTGTATCCCTGAGCTGATGAGA : 1200
Pho1a(9) : GAGATCAGGTGATCGTATTAAGTGGGAAGAGTTTCCTGAAAAAGTTGCTGTGCAGATGAATGACACTCACCCTACACTTTGTATCCCTGAGCTGATGAGA : 1200

                        *       1220          *       1240          *       1260          *       1280          *       1300
Pho1a-HR : ATATTGATAGATCTGAAGGGCTTGAATTGGAATGAAGCTTGGAATATTACTCAAAGAACTGTGGCCTACACAAACCATACTGTTTTGCCTGAGGCACTGG : 1300
Pho1a(2) : ATATTGATAGATCTGAAGGGCTTGAATTGGAATGAAGCTTGGAATATTACTCAAAGAACTGTGGCCTACACAAACCATACTGTTTTGCCTGAGGCACTGG : 1300
Pho1a(3) : ATATTGATAGATCTGAAGGGCTTGAATTGGAATGAAGCTTGGAATATTACTCAAAGAACTGTGGCCTACACAAACCATACTGTTTTGCCTGAGGCACTGG : 1300
Pho1a(4) : ATATTGATAGATCTGAAGGGCTTGAATTGGAATGAAGCTTGGAATATTACTCAAAGAACTGTGGCCTACACAAACCATACTGTTTTGCCTGAGGCACTGG : 1300
Pho1a-HA : ATATTGATAGATCTGAAGGGCTTGAATTGGAATGAAGCTTGGAATATTACTCAAAGAACTGTGGCCTACACAAACCATACTGTTTTGCCTGAGGCACTGG : 1300
Pho1a(6) : ATATTGATAGATCTGAAGGGCTTGAATTGGAATGAAGCTTGGAATATTACTCAAAGAACTGTGGCCTACACAAACCATACTGTTTTGCCTGAGGCACTGG : 1300
Pho1a(7) : ATATTGATAGATCTGAAGGGCTTGAATTGGAATGAAGCTTGGAATATTACTCAAAGAACTGTGGCCTACACAAACCATACTGTTTTGCCTGAGGCACTGG : 1300
Pho1a(8) : ATATTGATAGATCTGAAGGGCTTGAATTGGAATGAAGCTTGGAATATTACTCAAAGAACTGTGGCCTACACAAACCATACTGTTTTGCCTGAGGCACTGG : 1300
Pho1a(9) : ATATTGATAGATCTGAAGGGCTTGAATTGGAATGAAGCTTGGAATATTACTCAAAGAACTGTGGCCTACACAAACCATACTGTTTTGCCTGAGGCACTGG : 1300

                        *       1320          *       1340          *       1360          *       1380          *       1400
Pho1a-HR : AGAAATGGAGTTATGAATTGATGCAGAAACTCCTTCCCAGACATGTCGAAATCATTGAGGCGATTGACGAGGAGCTGGTACATGAAATTGTATTAAAATA : 1400
Pho1a(2) : AGAAATGGAGTTATGAATTGATGCAGAAACTCCTTCCCAGACATGTCGAAATCATTGAGGCGATTGACGAGGAGCTGGTACATGAAATTGTATTAAAATA : 1400
Pho1a(3) : AGAAATGGAGTTATGAATTGATGCAGAAACTCCTTCCCAGACATGTCGAAATCATTGAGGCGATTGACGAGGAGCTGGTACATGAAATTGTATTAAAATA : 1400
Pho1a(4) : AGAAATGGAGTTATGAATTGATGCAGAAACTCCTTCCCAGACATGTCGAAATCATTGAGGCGATTGACGAGGAGCTGGTACATGAAATTGTATTAAAATA : 1400
Pho1a-HA : AGAAATGGAGTTATGAATTGATGCAGAAACTCCTTCCCAGACATGTCGAAATCATTGAGGCGATTGACGAGGAGCTGGTACATGAAATTGTATTAAAATA : 1400
Pho1a(6) : AGAAATGGAGTTATGAATTGATGCAGAAACTCCTTCCCAGACATGTCGAAATCATTGAGGCGATTGACGAGGAGCTGGTACATGAAATTGTATTAAAATA : 1400
Pho1a(7) : AGAAATGGAGTTATGAATTGATGCAGAAACTCCTTCCCAGACATGTCGAAATCATTGAGGCGATTGACGAGGAGCTGGTACATGAAATTGTATTAAAATA : 1400
Pho1a(8) : AGAAATGGAGTTATGAATTGATGCAGAAACTCCTTCCCAGACATGTCGAAATCATTGAGGCGATTGACGAGGAGCTGGTACATGAAATTGTATTAAAATA : 1400
Pho1a(9) : AGAAATGGAGTTATGAATTGATGCAGAAACTCCTTCCCAGACATGTCGAAATCATTGAGGCGATTGACGAGGAGCTGGTACATGAAATTGTATTAAAATA : 1400

                        *       1420          *       1440          *       1460          *       1480          *       1500
Pho1a-HR : TGGTTCAATGGATCTGAACAAATTGGAGGAAAAGTTGACTACAATGAGAATCTTAGAAAATTTTGATCTTCCCAGTTCTGTTGCTGAATTATTTATTAAG : 1500
Pho1a(2) : TGGTTCAATGGATCTGAACAAATTGGAGGAAAAGTTGACTACAATGAGAATCTTAGAAAATTTTGATCTTCCCAGTTCTGTTGCTGAATTATTTATTAAG : 1500
Pho1a(3) : TGGTTCAATGGATCTGAACAAATTGGAGGAAAAGTTGACTACAATGAGAATCTTAGAAAATTTTGATCTTCCCAGTTCTGTTGCTGAATTATTTATTAAG : 1500
Pho1a(4) : TGGTTCAATGGATCTGAACAAATTGGAGGAAAAGTTGACTACAATGAGAATCTTAGAAAATTTTGATCTTCCCAGTTCTGTTGCTGAATTATTTATTAAG : 1500
Pho1a-HA : TGGTTCAATGGATCTGAACAAATTGGAGGAAAAGTTGACTACAATGAGAATCTTAGAAAATTTTGATCTTCCCAGTTCTGTTGCTGAATTATTTATTAAG : 1500
Pho1a(6) : TGGTTCAATGGATCTGAACAAATTGGAGGAAAAGTTGACTACAATGAGAATCTTAGAAAATTTTGATCTTCCCAGTTCTGTTGCTGAATTATTTATTAAG : 1500
Pho1a(7) : TGGTTCAATGGATCTGAACAAATTGGAGGAAAAGTTGACTACAATGAGAATCTTAGAAAATTTTGATCTTCCCAGTTCTGTTGCTGAATTATTTATTAAG : 1500
Pho1a(8) : TGGTTCAATGGATCTGAACAAATTGGAGGAAAAGTTGACTACAATGAGAATCTTAGAAAATTTTGATCTTCCCAGTTCTGTTGCTGAATTATTTATTAAG : 1500
Pho1a(9) : TGGTTCAATGGATCTGAACAAATTGGAGGAAAAGTTGACTACAATGAGAATCTTAGAAAATTTTGATCTTCCCAGTTCTGTTGCTGAATTATTTATTAAG : 1500

                        *       1520          *       1540          *       1560          *       1580          *       1600
Pho1a-HR : CCTGAAATCTCAGTTGATGATGATACTGAAACAGTAGAAGTCCATGACAAAGTTGAAGCTTCCGATAAAGTTGTGACTAATGATGAAGATGACACTGGTA : 1600
Pho1a(2) : CCTGAAATCTCAGTTGATGATGATACTGAAACAGTAGAAGTCCATGACAAAGTTGAAGCTTCCGATAAAGTTGTGACTAATGATGAAGATGACACTGGTA : 1600
Pho1a(3) : CCTGAAATCTCAGTTGATGATGATACTGAAACAGTAGAAGTCCATGACAAAGTTGAAGCTTCCGATAAAGTTGTGACTAATGATGAAGATGACACTGGTA : 1600
Pho1a(4) : CCTGAAATCTCAGTTGATGATGATACTGAAACAGTAGAAGTCCATGACAAAGTTGAAGCTTCCGATAAAGTTGTGACTAATGATGAAGATGACACTGGTA : 1600
Pho1a-HA : CCTGAAATCTCAGTTGATGATGATACTGAAACAGTAGAAGTCCATGACAAAGTTGAAGCTTCCGATAAAGTTGTGACTAATGATGAAGATGACACTGGTA : 1600
Pho1a(6) : CCTGAAATCTCAGTTGATGATGATACTGAAACAGTAGAAGTCCATGACAAAGTTGAAGCTTCCGATAAAGTTGTGACTAATGATGAAGATGACACTGGTA : 1600
Pho1a(7) : CCTGAAATCTCAGTTGATGATGATACTGAAACAGTAGAAGTCCATGACAAAGTTGAAGCTTCCGATAAAGTTGTGACTAATGATGAAGATGACACTGGTA : 1600
Pho1a(8) : CCTGAAATCTCAGTTGATGATGATACTGAAACAGTAGAAGTCCATGACAAAGTTGAAGCTTCCGATAAAGTTGTGACTAATGATGAAGATGACACTGGTA : 1600
Pho1a(9) : CCTGAAATCTCAGTTGATGATGATACTGAAACAGTAGAAGTCCATGACAAAGTTGAAGCTTCCGATAAAGTTGTGACTAATGATGAAGATGACACTGGTA : 1600

                        *       1620          *       1640          *       1660          *       1680          *       1700
Pho1a-HR : AGAAAACTAGTGTGAAGATAGAAGCAGCTGCAGAAAAAGACATTGACAAGAAAACTCCCGTGAGTCCGGAACCAGCTGTTATACCACCTAAGAAGGTACG : 1700
Pho1a(2) : AGAAAACTAGTGTGAAGATAGAAGCAGCTGCAGAAAAAGACATTGACAAGAAAACTCCCGTGAGTCCGGAACCAGCTGTTATACCACCTAAGAAGGTACG : 1700
Pho1a(3) : AGAAAACTAGTGTGAAGATAGAAGCAGCTGCAGAAAAAGACATTGACAAGAAAACTCCCGTGAGTCCGGAACCAGCTGTTATACCACCTAAGAAGGTACG : 1700
Pho1a(4) : AGAAAACTAGTGTGAAGATAGAAGCAGCTGCAGAAAAAGACATTGACAAGAAAACTCCCGTGAGTCCGGAACCAGCTGTTATACCACCTAAGAAGGTACG : 1700
Pho1a-HA : AGAAAACTAGTGTGAAGATAGAAGCAGCTGCAGAAAAAGACATTGACAAGAAAACTCCCGTGAGTCCGGAACCAGCTGTTATACCACCTAAGAAGGTACG : 1700
Pho1a(6) : AGAAAACTAGTGTGAAGATAGAAGCAGCTGCAGAAAAAGACATTGACAAGAAAACTCCCGTGAGTCCGGAACCAGCTGTTATACCACCTAAGAAGGTACG : 1700
Pho1a(7) : AGAAAACTAGTGTGAAGATAGAAGCAGCTGCAGAAAAAGACATTGACAAGAAAACTCCCGTGAGTCCGGAACCAGCTGTTATACCACCTAAGAAGGTACG : 1700
Pho1a(8) : AGAAAACTAGTGTGAAGATAGAAGCAGCTGCAGAAAAAGACATTGACAAGAAAACTCCCGTGAGTCCGGAACCAGCTGTTATACCACCTAAGAAGGTACG : 1700
Pho1a(9) : AGAAAACTAGTGTGAAGATAGAAGCAGCTGCAGAAAAAGACATTGACAAGAAAACTCCCGTGAGTCCGGAACCAGCTGTTATACCACCTAAGAAGGTACG : 1700
```

```
                    *      1720         *      1740         *      1760         *      1780         *      1800
Pho1a-HR : CATGGCCAACTTGTGTGTTGTGGGCGGCCATGCTGTTAATGGAGTTGCTGAGATCCATAGTGAAATTGTGAAGGAGGAGGTTTTCAATGACTTCTATGAG : 1800
Pho1a(2) : CATGGCCAACTTGTGTGTTGTGGGCGGCCATGCTGTTAATGGAGTTGCTGAGATCCATAGTGAAATTGTGAAGGAGGAGGTTTTCAATGACTTCTATGAG : 1800
Pho1a(3) : CATGGCCAACTTGTGTGTTGTGGGCGGCCATGCTGTTAATGGAGTTGCTGAGATCCATAGTGAAATTGTGAAGGAGGAGGTTTTCAATGACTTCTATGAG : 1800
Pho1a(4) : CATGGCCAACTTGTGTGTTGTGGGCGGCCATGCTGTTAATGGAGTTGCTGAGATCCATAGTGAAATTGTGAAGGAGGAGGTTTTCAATGACTTCTATGAG : 1800
Pho1a-HA : CATGGCCAACTTGTGTGTTGTGGGCGGCCATGCTGTTAATGGAGTTGCTGAGATCCATAGTGAAATTGTGAAGGAGGAGGTTTTCAATGACTTCTATGAG : 1800
Pho1a(6) : CATGGCCAACTTGTGTGTTGTGGGCGGCCATGCTGTTAATGGAGTTGCTGAGATCCATAGTGAAATTGTGAAGGAGGAGGTTTTCAATGACTTCTATGAG : 1800
Pho1a(7) : CATGGCCAACTTGTGTGTTGTGGGCGGCCATGCTGTTAATGGAGTTGCTGAGATCCATAGTGAAATTGTGAAGGAGGAGGTTTTCAATGACTTCTATGAG : 1800
Pho1a(8) : CATGGCCAACTTGTGTGTTGTGGGCGGCCATGCTGTTAATGGAGTTGCTGAGATCCATAGTGAAATTGTGAAGGAGGAGGTTTTCAATGACTTCTATGAG : 1800
Pho1a(9) : CATGGCCAACTTGTGTGTTGTGGGCGGCCATGCTGTTAATGGAGTTGCTGAGATCCATAGTGAAATTGTGAAGGAGGAGGTTTTCAATGACTTCTATGAG : 1800

                    *      1820         *      1840         *      1860         *      1880         *      1900
Pho1a-HR : CTCTGGCCGGAAAAGTTCCAAAACAAAACAAATGGAGTGACTCCAAGAAGATGGATTCGTTTCTGCAATCCTCCTCTTAGTGCCATCATAACTAAGTGGA : 1900
Pho1a(2) : CTCTGGCCGGAAAAGTTCCAAAACAAAACAAATGGAGTGACTCCAAGAAGATGGATTCGTTTCTGCAATCCTCCTCTTAGTGCCATCATAACTAAGTGGA : 1900
Pho1a(3) : CTCTGGCCGGAAAAGTTCCAAAACAAAACAAATGGAGTGACTCCAAGAAGATGGATTCGTTTCTGCAATCCTCCTCTTAGTGCCATCATAACTAAGTGGA : 1900
Pho1a(4) : CTCTGGCCGGAAAAGTTCCAAAACAAAACAAATGGAGTGACTCCAAGAAGATGGATTCGTTTCTGCAATCCTCCTCTTAGTGCCATCATAACTAAGTGGA : 1900
Pho1a-HA : CTCTGGCCGGAAAAGTTCCAAAACAAAACAAATGGAGTGACTCCAAGAAGATGGATTCGTTTCTGCAATCCTCCTCTTAGTGCCATCATAACTAAGTGGA : 1900
Pho1a(6) : CTCTGGCCGGAAAAGTTCCAAAACAAAACAAATGGAGTGACTCCAAGAAGATGGATTCGTTTCTGCAATCCTCCTCTTAGTGCCATCATAACTAAGTGGA : 1900
Pho1a(7) : CTCTGGCCGGAAAAGTTCCAAAACAAAACAAATGGAGTGACTCCAAGAAGATGGATTCGTTTCTGCAATCCTCCTCTTAGTGCCATCATAACTAAGTGGA : 1900
Pho1a(8) : CTCTGGCCGGAAAAGTTCCAAAACAAAACAAATGGAGTGACTCCAAGAAGATGGATTCGTTTCTGCAATCCTCCTCTTAGTGCCATCATAACTAAGTGGA : 1900
Pho1a(9) : CTCTGGCCGGAAAAGTTCCAAAACAAAACAAATGGAGTGACTCCAAGAAGATGGATTCGTTTCTGCAATCCTCCTCTTAGTGCCATCATAACTAAGTGGA : 1900

                    *      1920         *      1940         *      1960         *      1980         *      2000
Pho1a-HR : CTGGTACAGAGGATTGGGTCCTGAAAACTGAAAAGTTGGCAGAATTGCAGAAGTTTGCTGATAATGAAGATCTTCAAAATGAGTGGAGGGAAGCAAAAAG : 2000
Pho1a(2) : CTGGTACAGAGGATTGGGTCCTGAAAACTGAAAAGTTGGCAGAATTGCAGAAGTTTGCTGATAATGAAGATCTTCAAAATGAGTGGAGGGAAGCAAAAAG : 2000
Pho1a(3) : CTGGTACAGAGGATTGGGTCCTGAAAACTGAAAAGTTGGCAGAATTGCAGAAGTTTGCTGATAATGAAGATCTTCAAAATGAGTGGAGGGAAGCAAAAAG : 2000
Pho1a(4) : CTGGTACAGAGGATTGGGTCCTGAAAACTGAAAAGTTGGCAGAATTGCAGAAGTTTGCTGATAATGAAGATCTTCAAAATGAGTGGAGGGAAGCAAAAAG : 2000
Pho1a-HA : CTGGTACAGAGGATTGGGTCCTTAAAACTGAAAAGTTGTCAGAATTGCAGAAGTTTGCTGATAATGAAGATCTTCAAAATGAGTGGAGGGAAGCAAAAAG : 2000
Pho1a(6) : CTGGTACAGAGGATTGGGTCCTTAAAACTGAAAAGTTGTCAGAATTGCAGAAGTTTGCTGATAATGAAGATCTTCAAAATGAGTGGAGGGAAGCAAAAAG : 2000
Pho1a(7) : CTGGTACAGAGGATTGGGTCCTGAAAACTGAAAAGTTGGCAGAATTGCAGAAGTTTGCTGATAATGAAGATCTTCAAAATGAGTGGAGGGAAGCAAAAAG : 2000
Pho1a(8) : CTGGTACAGAGGATTGGGTCCTGAAAACTGAAAAGTTGGCAGAATTGCAGAAGTTTGCTGATAATGAAGATCTTCAAAATGAGTGGAGGGAAGCAAAAAG : 2000
Pho1a(9) : CTGGTACAGAGGATTGGGTCCTGAAAACTGAAAAGTTGGCAGAATTGCAGAAGTTTGCTGATAATGAAGATCTTCAAAATGAGTGGAGGGAAGCAAAAAG : 2000

                    *      2020         *      2040         *      2060         *      2080         *      2100
Pho1a-HR : GAGCAACAAGATTAAAGTTGTCTCCTTTCTCAAAGAAAAGACAGGGTATTCTGTTGTCCCAGATGCAATGTTTGATATTCAGGTAAAACGCATTCATGAG : 2100
Pho1a(2) : GAGCAACAAGATTAAAGTTGTCTCCTTTCTCAAAGAAAAGACAGGGTATTCTGTTGTCCCAGATGCAATGTTTGATATTCAGGTAAAACGCATTCATGAG : 2100
Pho1a(3) : GAGCAACAAGATTAAAGTTGTCTCCTTTCTCAAAGAAAAGACAGGGTATTCTGTTGTCCCAGATGCAATGTTTGATATTCAGGTAAAACGCATTCATGAG : 2100
Pho1a(4) : GAGCAACAAGATTAAAGTTGTCTCCTTTCTCAAAGAAAAGACAGGGTATTCTGTTGTCCCAGATGCAATGTTTGATATTCAGGTAAAACGCATTCATGAG : 2100
Pho1a-HA : GAGCAACAAGATTAAAGTTGTCTCCTTTCTCAAAGAAAAGACAGGGTATTCTGTTGTCCCAGATGCAATGTTTGATATTCAGGTAAAACGCATTCATGAG : 2100
Pho1a(6) : GAGCAACAAGATTAAAGTTGTCTCCTTTCTCAAAGAAAAGACAGGGTATTCTGTTGTCCCAGATGCAATGTTTGATATTCAGGTAAAACGCATTCATGAG : 2100
Pho1a(7) : GAGCAACAAGATTAAAGTTGTCTCCTTTCTCAAAGAAAAGACAGGGTATTCTGTTGTCCCAGATGCAATGTTTGATATTCAGGTAAAACGCATTCATGAG : 2100
Pho1a(8) : GAGCAACAAGATTAAAGTTGTCTCCTTTCTCAAAGAAAAGACAGGGTATTCTGTTGTCCCAGATGCAATGTTTGATATTCAGGTAAAACGCATTCATGAG : 2100
Pho1a(9) : GAGCAACAAGATTAAAGTTGTCTCCTTTCTCAAAGAAAAGACAGGGTATTCTGTTGTCCCAGATGCAATGTTTGATATTCAGGTAAAACGCATTCATGAG : 2100

                    *      2120         *      2140         *      2160         *      2180         *      2200
Pho1a-HR : TACAAGCGACAACTGTTAAATATCTTCGGCATCGTTTATCGGTATAAGAAGATGAAAGAAATGACAGCTGCAGAAAGAAAGACTAACTTCGTTCCTCGAG : 2200
Pho1a(2) : TACAAGCGACAACTGTTAAATATCTTCGGCATCGTTTATCGGTATAAGAAGATGAAAGAAATGACAGCTGCAGAAAGAAAGACTAACTTCGTTCCTCGAG : 2200
Pho1a(3) : TACAAGCGACAACTGTTAAATATCTTCGGCATCGTTTATCGGTATAAGAAGATGAAAGAAATGACAGCTGCAGAAAGAAAGACTAACTTCGTTCCTCGAG : 2200
Pho1a(4) : TACAAGCGACAACTGTTAAATATCTTCGGCATCGTTTATCGGTATAAGAAGATGAAAGAAATGACAGCTGCAGAAAGAAAGACTAACTTCGTTCCTCGAG : 2200
Pho1a-HA : TACAAGCGACAACTGTTAAATATCTTCGGCATCGTTTATCGGTATAAGAAGATGAAAGAAATGACAGCTGCAGAAAGAAAGACTAACTTCGTTCCTCGAG : 2200
Pho1a(6) : TACAAGCGACAACTGTTAAATATCTTCGGCATCGTTTATCGGTATAAGAAGATGAAAGAAATGACAGCTGCAGAAAGAAAGACTAACTTCGTTCCTCGAG : 2200
Pho1a(7) : TACAAGCGACAACTGTTAAATATCTTCGGCATCGTTTATCGGTATAAGAAGATGAAAGAAATGACAGCTGCAGAAAGAAAGACTAACTTCGTTCCTCGAG : 2200
Pho1a(8) : TACAAGCGACAACTGTTAAATATCTTCGGCATCGTTTATCGGTATAAGAAGATGAAAGAAATGACAGCTGCAGAAAGAAAGACTAACTTCGTTCCTCGAG : 2200
Pho1a(9) : TACAAGCGACAACTGTTAAATATCTTCGGCATCGTTTATCGGTATAAGAAGATGAAAGAAATGACAGCTGCAGAAAGAAAGACTAACTTCGTTCCTCGAG : 2200

                    *      2220         *      2240         *      2260         *      2280         *      2300
Pho1a-HR : TATGCATATTTGGGGGAAAAGCTTTTGCCACATATGTGCAAGCCAAGAGGATTGTAAAATTTATCACAGATGTTGGTGCTACTATAAATCATGATCCAGA : 2300
Pho1a(2) : TATGCATATTTGGGGGAAAAGCTTTTGCCACATATGTGCAAGCCAAGAGGATTGTAAAATTTATCACAGATGTTGGTGCTACTATAAATCATGATCCAGA : 2300
Pho1a(3) : TATGCATATTTGGGGGAAAAGCTTTTGCCACATATGTGCAAGCCAAGAGGATTGTAAAATTTATCACAGATGTTGGTGCTACTATAAATCATGATCCAGA : 2300
Pho1a(4) : TATGCATATTTGGGGGAAAAGCTTTTGCCACATATGTGCAAGCCAAGAGGATTGTAAAATTTATCACAGATGTTGGTGCTACTATAAATCATGATCCAGA : 2300
Pho1a-HA : TATGCATATTTGGGGGAAAAGCTTTTGCCACATATGTGCAAGCCAAGAGGATTGTAAAATTTATCACAGATGTTGGTGCTACTATAAATCATGATCCAGA : 2300
Pho1a(6) : TATGCATATTTGGGGGAAAAGCTTTTGCCACATATGTGCAAGCCAAGAGGATTGTAAAATTTATCACAGATGTTGGTGCTACTATAAATCATGATCCAGA : 2300
Pho1a(7) : TATGCATATTTGGGGGAAAAGCTTTTGCCACATATGTGCAAGCCAAGAGGATTGTAAAATTTATCACAGATGTTGGTGCTACTATAAATCATGATCCAGA : 2300
Pho1a(8) : TATGCATATTTGGGGGAAAAGCTTTTGCCACATATGTGCAAGCCAAGAGGATTGTAAAATTTATCACAGATGTTGGTGCTACTATAAATCATGATCCAGA : 2300
Pho1a(9) : TATGCATATTTGGGGGAAAAGCTTTTGCCACATATGTGCAAGCCAAGAGGATTGTAAAATTTATCACAGATGTTGGTGCTACTATAAATCATGATCCAGA : 2300

                    *      2320         *      2340         *      2360         *      2380         *      2400
Pho1a-HR : AATCGGTGATCTGTTGAAGGTAGTCTTTGTGCCAGATTACAATGTCAGTGTTGCTGAATTGCTAATTCCTGCTAGCGATCTATCAGAACATATCAGTACG : 2400
Pho1a(2) : AATCGGTGATCTGTTGAAGGTAGTCTTTGTGCCAGATTACAATGTCAGTGTTGCTGAATTGCTAATTCCTGCTAGCGATCTATCAGAACATATCAGTACG : 2400
Pho1a(3) : AATCGGTGATCTGTTGAAGGTAGTCTTTGTGCCAGATTACAATGTCAGTGTTGCTGAATTGCTAATTCCTGCTAGCGATCTATCAGAACATATCAGTACG : 2400
Pho1a(4) : AATCGGTGATCTGTTGAAGGTAGTCTTTGTGCCAGATTACAATGTCAGTGTTGCTGAATTGCTAATTCCTGCTAGCGATCTATCAGAACATATCAGTACG : 2400
Pho1a-HA : AATCGGTGATCTGTTGAAGGTAGTCTTTGTGCCAGATTACAATGTCAGTGTTGCTGAATTGCTAATTCCTGCTAGCGATCTATCAGAACATATCAGTACG : 2400
Pho1a(6) : AATCGGTGATCTGTTGAAGGTAGTCTTTGTGCCAGATTACAATGTCAGTGTTGCTGAATTGCTAATTCCTGCTAGCGATCTATCAGAACATATCAGTACG : 2400
Pho1a(7) : AATCGGTGATCTGTTGAAGGTAGTCTTTGTGCCAGATTACAATGTCAGTGTTGCTGAATTGCTAATTCCTGCTAGCGATCTATCAGAACATATCAGTACG : 2400
Pho1a(8) : AATCGGTGATCTGTTGAAGGTAGTCTTTGTGCCAGATTACAATGTCAGTGTTGCTGAATTGCTAATTCCTGCTAGCGATCTATCAGAACATATCAGTACG : 2400
Pho1a(9) : AATCGGTGATCTGTTGAAGGTAGTCTTTGTGCCAGATTACAATGTCAGTGTTGCTGAATTGCTAATTCCTGCTAGCGATCTATCAGAACATATCAGTACG : 2400

                    *      2420         *      2440         *      2460         *      2480         *      2500
Pho1a-HR : GCTGGAATGGAGGCCAGTGGAACCAGTAATATGAAGTTTGCAATGAATGGTTGTATCCAAATTGGTACATTGGATGGCGCTAATGTTGAAATAAGGGAAG : 2500
Pho1a(2) : GCTGGAATGGAGGCCAGTGGAACCAGTAATATGAAGTTTGCAATGAATGGTTGTATCCAAATTGGTACATTGGATGGCGCTAATGTTGAAATAAGGGAAG : 2500
Pho1a(3) : GCTGGAATGGAGGCCAGTGGAACCAGTAATATGAAGTTTGCAATGAATGGTTGTATCCAAATTGGTACATTGGATGGCGCTAATGTTGAAATAAGGGAAG : 2500
Pho1a(4) : GCTGGAATGGAGGCCAGTGGAACCAGTAATATGAAGTTTGCAATGAATGGTTGTATCCAAATTGGTACATTGGATGGCGCTAATGTTGAAATAAGGGAAG : 2500
Pho1a-HA : GCTGGAATGGAGGCCAGTGGAACCAGTAATATGAAGTTTGCAATGAATGGTTGTATCCAAATTGGTACATTGGATGGCGCTAATGTTGAAATAAGGGAAG : 2500
Pho1a(6) : GCTGGAATGGAGGCCAGTGGAACCAGTAATATGAAGTTTGCAATGAATGGTTGTATCCAAATTGGTACATTGGATGGCGCTAATGTTGAAATAAGGGAAG : 2500
Pho1a(7) : GCTGGAATGGAGGCCAGTGGAACCAGTAATATGAAGTTTGCAATGAATGGTTGTATCCAAATTGGTACATTGGATGGCGCTAATGTTGAAATAAGGGAAG : 2500
Pho1a(8) : GCTGGAATGGAGGCCAGTGGAACCAGTAATATGAAGTTTGCAATGAATGGTTGTATCCAAATTGGTACATTGGATGGCGCTAATGTTGAAATAAGGGAAG : 2500
Pho1a(9) : GCTGGAATGGAGGCCAGTGGAACCAGTAATATGAAGTTTGCAATGAATGGTTGTATCCAAATTGGTACATTGGATGGCGCTAATGTTGAAATAAGGGAAG : 2500

                    *      2520         *      2540         *      2560         *      2580         *      2600
Pho1a-HR : AGGTTGGAGAAGAAAACTTCTTTCTCTTTGGTGCTCAAGCTCATGAAATTGCAGGGCTTAGAAAAGAAAGAGCTGACGGAAAGTTTGTACCTGATGAACG : 2600
Pho1a(2) : AGGTTGGAGAAGAAAACTTCTTTCTCTTTGGTGCTCAAGCTCATGAAATTGCAGGGCTTAGAAAAGAAAGAGCTGACGGAAAGTTTGTACCTGATGAACG : 2600
Pho1a(3) : AGGTTGGAGAAGAAAACTTCTTTCTCTTTGGTGCTCAAGCTCATGAAATTGCAGGGCTTAGAAAAGAAAGAGCTGACGGAAAGTTTGTACCTGATGAACG : 2600
Pho1a(4) : AGGTTGGAGAAGAAAACTTCTTTCTCTTTGGTGCTCAAGCTCATGAAATTGCAGGGCTTAGAAAAGAAAGAGCTGACGGAAAGTTTGTACCTGATGAACG : 2600
Pho1a-HA : AGGTTGGAGAAGAAAACTTCTTTCTCTTTGGTGCTCAAGCTCATGAAATTGCAGGGCTTAGAAAAGAAAGAGCTGACGGAAAGTTTGTACCTGATGAACG : 2600
Pho1a(6) : AGGTTGGAGAAGAAAACTTCTTTCTCTTTGGTGCTCAAGCTCATGAAATTGCAGGGCTTAGAAAAGAAAGAGCTGACGGAAAGTTTGTACCTGATGAACG : 2600
Pho1a(7) : AGGTTGGAGAAGAAAACTTCTTTCTCTTTGGTGCTCAAGCTCATGAAATTGCAGGGCTTAGAAAAGAAAGAGCTGACAGAAAGTTTGTACCTGATGAACG : 2600
Pho1a(8) : AGGTTGGAGAAGAAAACTTCTTTCTCTTTGGTGCTCAAGCTCATGAAATTGCAGGGCTTAGAAAAGAAAGAGCTGACAGAAAGTTTGTACCTGATGAACG : 2600
Pho1a(9) : AGGTTGGAGAAGAAAACTTCTTTCTCTTTGGTGCTCAAGCTCATGAAATTGCAGGGCTTAGAAAAGAAAGAGCTGACAGAAAGTTTGTACCTGATGAACG : 2600
```

```
                    *      2620         *      2640         *      2660         *      2680         *      2700
Pho1a-HR : TTTTGAAGAGGTGAAGGAATTTGTTAGAAGCGGTGCTTTTGGCTCTTATAACTATGATGACCTAATTGGATCGTTGGAAGGAAATGAAGGTTTTGGCCGT : 2700
Pho1a(2) : TTTTGAAGAGGTGAAGGAATTTGTTAGAAGCGGTGCTTTTGGCTCTTATAACTATGATGACCTAATTGGATCGTTGGAAGGAAATGAAGGTTTTGGCCGT : 2700
Pho1a(3) : TTTTGAAGAGGTGAAGGAATTTGTTAGAAGCGGTGCTTTTGGCTCTTATAACTATGATGACCTAATTGGATCGTTGGAAGGAAATGAAGGTTTTGGCCGT : 2700
Pho1a(4) : TTTTGAAGAGGTGAAGGAATTTGTTAGAAGCGGTGCTTTTGGCTCTTATAACTATGATGACCTAATTGGATCGTTGGAAGGAAATGAAGGTTTTGGCCGT : 2700
Pho1a-HA : TTTTGAAGAGGTGAAGGAATTTGTTAGAAGCGGTGCTTTTGGCTCTTATAACTATGATGACCTAATTGGATCGTTGGAAGGAAATGAAGGTTTTGGCCGT : 2700
Pho1a(6) : TTTTGAAGAGGTGAAGGAATTTGTTAGAAGCGGTGCTTTTGGCTCTTATAACTATGATGACCTAATTGGATCGTTGGAAGGAAATGAAGGTTTTGGCCGT : 2700
Pho1a(7) : TTTTGAAGAGGTGAAGGAATTTGTTAGAAGCGGTGCTTTTGGCTCTTATAACTATGATGACCTAATTGGATCGTTGGAAGGAAATGAAGGTTTTGGCCGT : 2700
Pho1a(8) : TTTTGAAGAGGTGAAGGAATTTGTTAGAAGCGGTGCTTTTGGCTCTTATAACTATGATGACCTAATTGGATCGTTGGAAGGAAATGAAGGTTTTGGCCGT : 2700
Pho1a(9) : TTTTGAAGAGGTGAAAGAATTTGTTAGAAGCGGTGCTTTTGGCTCTTATAACTATGATGACCTAATTGGATCGTTGGAAGGAAATGAAGGTTTTGGCCGT : 2700

                    *      2720         *      2740         *      2760         *      2780         *      2800
Pho1a-HR : GCTGACTATTTCCTTGTGGGCAAGGACTTCCCCAGTTACATAGAATGCCAAGAGAAAGTTGATGAGGCATATCGCGACCAGAAAAGGTGGACAACGATGT : 2800
Pho1a(2) : GCTGACTATTTCCTTGTGGGCAAGGACTTCCCCAGTTACATAGAATGCCAAGAGAAAGTTGATGAGGCATATCGCGACCAGAAAAGGTGGACAAAGATGT : 2800
Pho1a(3) : GCTGACTATTTCCTTGTGGGCAAGGACTTCCCCAGTTACATAGAATGCCAAGAGAAAGTTGATGAGGCATATCGCAACCAGAAAAGGTGGACAAAGATGT : 2800
Pho1a(4) : GCTGACTATTTCCTTGTGGGCAAGGACTTCCCCAGTTACATAGAATGCCAAGAGAAAGTTGATGAGGCATATCGCGACCAGAAAAGGTGGACAACGATGT : 2800
Pho1a-HA : GCTGACTATTTCCTTGTGGGCAAGGACTTCCCCAGTTACATAGAATGCCAAGAGAAAGTTGATGAGGCATATCGCAACCAGAAAAGGTGGACAAAGATGT : 2800
Pho1a(6) : GCTGACTATTTCCTTGTGGGCAAGGACTTCCCCAGTTACATAGAATGCCAAGAGAAAGTTGATGAGGCATATCGCGACCAGAAAAGGTGGACAAAGATGT : 2800
Pho1a(7) : GCTGACTATTTCCTTGTGGGCAAGGACTTCCCCAGTTACATAGAATGCCAAGAAAAAGTTGATGAGGCATATCGCGACCAGAAAAGGTGGACAAAGATGT : 2800
Pho1a(8) : GCTGACTATTTCCTTGTGGGCAAGGACTTCCCCAGTTACATAGAATGCCAAGAAAAAGTTGATGAGGCATATCGCGACCAGAAAAGGTGGACAAAGATGT : 2800
Pho1a(9) : GCTGACTATTTCCTTGTGGGCAAGGACTTCCCCAGTTACATAGAATGCCAAGAGAAAGTTGATGTGGCATATCGCGACCAGAAAAGGTGGACAAAGATGT : 2800

                    *      2820         *      2840         *      2860         *      2880         *      2900
Pho1a-HR : CAATCTTGAATACAGCGGGATCGTACAAGTTCAGCAGTGACAGAACAATCCATGAATATGCCAAAGACATTTGGAACATTGAAGCTGTGGAAATAGCATA : 2900
Pho1a(2) : CAATCTTGAATACAGCGGGATCCTACAAGTTCAGCAGTGACAGAACAATCCATGAATATGCCAAAGACATTTGGAACATTGAAGCTGTGGAAATAGCATA : 2900
Pho1a(3) : CAATCTTGAATACAGCGGGATCCTACAAGTTCAGCAGTGACAGAACAATCCATGAATATGCCAAAGACATTTGGAACATTGAAGCTGTGGAAATAGCATA : 2900
Pho1a(4) : CAATCTTGAATACAGCGGGATCGTACAAGTTCAGCAGTGACAGAACAATCCATGAATATGCCAAAGACATTTGGAACATTGAAGCTGTGGAAATAGCATA : 2900
Pho1a-HA : CAATCTTGAATACAGCGGGATCCTACAAGTTCAGCAGTGACAGAACAATCCATGAATATGCCAAAGACATTTGGAACATTGAAGCTGTGGAAATAGCATA : 2900
Pho1a(6) : CAATCTTGAATACAGCGGGATCCTACAAGTTCAGCAGTGACAGAACAATCCATGAATATGCCAAAGACATTTGGAACATTGAAGCTGTGGAAATAGCATA : 2900
Pho1a(7) : CAATCTTGAATACAGCGGGATCCTACAAGTTCAGCAGTGACAGAACAATCCATGAATATGCCAAAGACATTTGGAACATTGAAGCTGTGGAAATAGCATA : 2900
Pho1a(8) : CAATCTTGAATACAGCGGGATCGTACAAGTTCAGCAGTGACAGAACAATCCATGAATATGCCAAAGACATTTGGAACATTGAAGCTGTGGAAATAGCATA : 2900
Pho1a(9) : CAATCTTGAATACAGCGGGATCGTACAAGTTCAGCAGTGACAGAACAATCCATGAATATGCCAAAGACATTTGGAACATTGAAGCTGTGGAAATAGCATA : 2900


Pho1a-HR : A : 2901
Pho1a(2) : A : 2901
Pho1a(3) : A : 2901
Pho1a(4) : A : 2901
Pho1a-HA : A : 2901
Pho1a(6) : A : 2901
Pho1a(7) : A : 2901
Pho1a(8) : A : 2901
Pho1a(9) : A : 2901
```

Figure S1 Sequence alignment of nine Pho1a cDNA alleles.

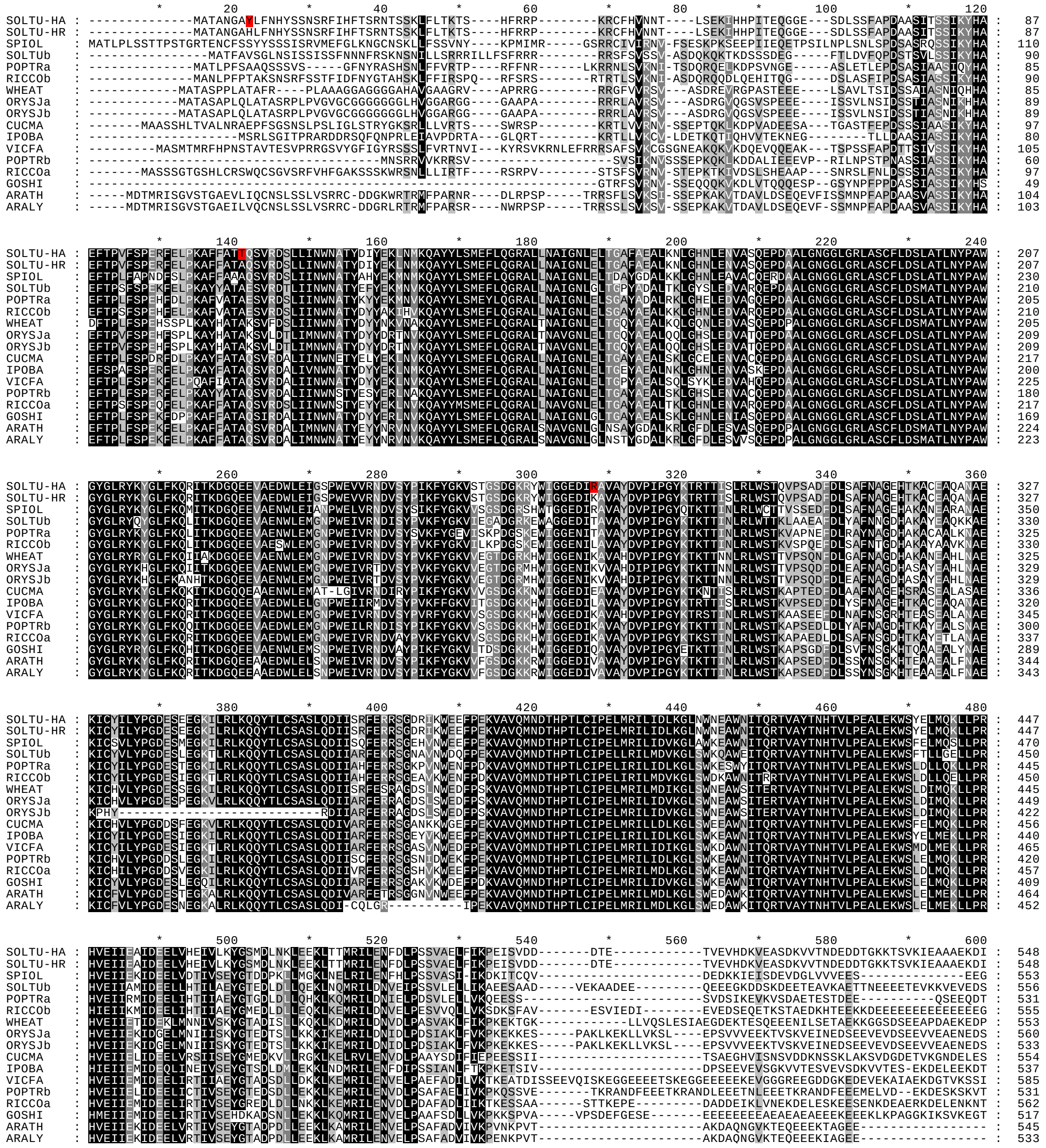

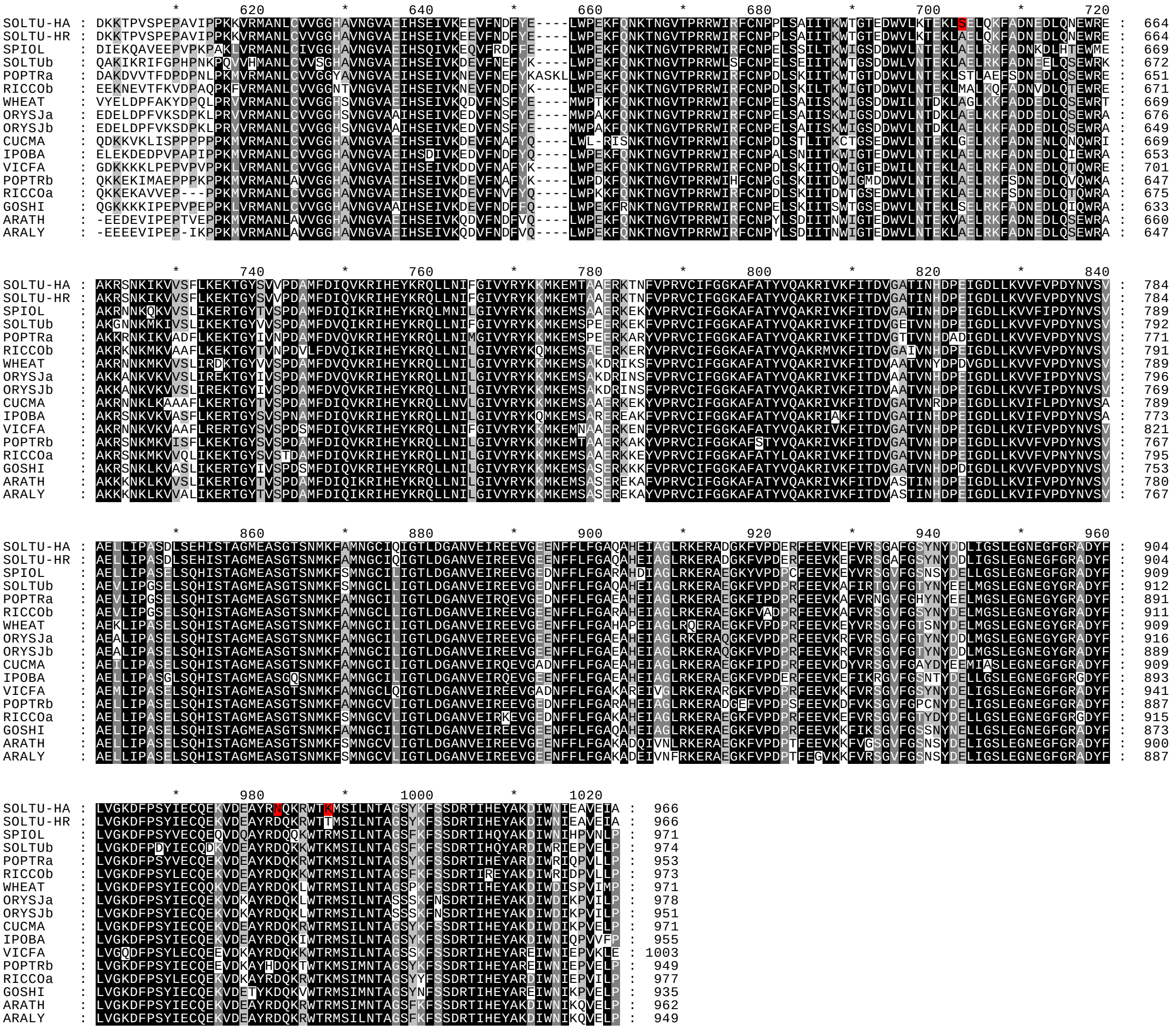

Figure S2 Alignment of plant PHO1a deduced protein sequences. *Solanum tuberosum*: SOLTU-HA (Pho1a-H_A, this paper), SOLTU-HR (P04045); *Spinacea oleracea*: SPIOL (O24363); *Populus trichocarpa:* POPTRa (B9HXL0), POPTRb (B9H0D3); *Ricinus communis*: RICCOa (B9SJB6); RICCOb (B9RCW0); *Triticum aestivum*: WHEAT (B2LXU4); *Oryza sativa* ssp. *japonica*: ORYSJa (B3IYE3), ORYSJb (Q9AUX8); *Cucurbita maxima*: CUCMA (B2DG13); *Ipomea batatas*: IPOBA (P27598); *Vicia faba*: VICFA (P53536); *Gossypium hirsutum*: GOSHI (D2D337); *Arabidopsis thaliana*: ARATH (Q9LIB2); *Arabidopsis lyrata*: ARALY (D7LNX4). The six amino acid changes in Pho1a-H_A versus Pho1a-H_R are highlighted red.

File S1

Amplified potato genomic fragments including SNP positions, nucleotide alleles and identifications.

Sequences were retrieved from pseudomolecules (v4.03) at http://potato.plantbiology.msu.edu/cgi-bin/gbrowse/potato/. Primer sequences are underlined. SNP numbering starts with A = 1 in the ATG initiation codon in the genomic locus PGSC0003DMG*********.

1. Glucan water dikinase, *GWD* (PGSC0003DMG400007677)

chr05:9827209..9827979

```
ATAAAAGTCAAAGCAAAGAAGAGCCTCTTCATGTAACAAAGAGTGAAATACCTGATGACCTTGCCCAAGCACAAGCTTACATT
AGGTGGGAGAAAGCAGGAAAGCCGAACTATCCTCCAGAAAA
                    3058              3072                3089
GCAAATTGTAAATGCTGAAC(T/C)TTTCTTACAGTTT(A/T)TGTAGTGTGTGTATAT(G/
    3092            3104         3113
C)TC(T/C)AGGCGCATAAA(T/G)CTGGGTAT(T/G)CTGTCTTCTATAGATGTGAGACTT
       3145
TGATCAA(G/A)CATTGTTTTATTAACAGGAAGAACTCGAAGAAGCAAGAAGAGAATTGCAA
                                                  3246
CTTGAGCTTGAGAAAGGCATTACCCTTGATGAGTTGCGGAAAAAGATTAC(A/C)AAAGGGG
     3259          3269           3280  3281           3291
AGATA(A/G)AAACTAAGG(C/T)GGAAAAGCAC(G/C)(T/A/C)GAAAAGAAG(C/T)TC
    3298                                         3339
TTTT(G/T)CCGTTGAAAGAATCCAAAGAAAGAAGAGAGACTTTGGGCA(G/T)CTTATTAA
       3355                3372                  3389
TAAGTAT(C/A)CTTCCAGTCCTGCAGT(A/G)CAAGTACAAAAGGTCT(/C)GGAAGAAC
  3400                   3419  3421
CA(C/G)CAGCCTTATCTAAAATTA(A/G)G(C/A)TGTATGCCAAGGAGAAGGAGGAGCAG
    3452             3465    3469                  3487
ATTG(A/G)TGATCCGATCCT(A/T)AAT(G/A)AAAAGATCTTTAAGGTC(G/A)ATGATG
          3504                                            3550
GGGAGCTACT(G/T)GTGAGTGCTAACTTATTCTTGACTGTAACCACTAATCTCAGAAGC(T
          3558                                          3600
/A)AGTTTGA(A/G)TGCATATTGAGAATCTTCCATTGTCTCCACATTTTAAGGTA(C/G)T
    3606
GGTA(G/T)CAAAGTCCTCTGGGAAGACAAAAGTACATCTAGCTACAGATCTGAATCAGCCAATTACTCTTCACTGGGCATTA
TCC
```

2. Phosphoglucan water dikinase, *PWD* (PGSC0003DMG400016613)

chr09:60571111..60571981

```
GGTCTGATGATCTATCTGATTGCAGTTTATAGTGACCAAGGTGCACCAGCATCATTTAACGT
      10503                                     10543  10547
TCCTGC[A/T]GGAGCAGTTATTCCATTTGGTTCCATGGAAACGGCATTG[G/A]AAA[T/C
                            10576
]GAACAAGTTAATGGAGACCTTCACATTG[C/T]TTGTCGAACAGATAGAAACAGCTGAAAT
                       10629                          10657
TGATGGCGGTGAACTTGATAAACA[T/C]TGTGAGGATCTCCAGAAGTTAATATCT[T/A]C
                             10689         10700 10701
TCTATTGCCTGGACAAGATGTCATTGAAAG[C/G]TTGGGAGAAG[T/C][A/G]TTTCCCG
                                    10746          10758
GTAATGCACGTTTAATAGTGCGTTCAAGTGCTAATGT[C/T]GAGGACTTGGC[G/A]GG[G
10761      10770  10773
/A]ATGTCAGC[T/A]GC[T/C]GGACTTTATGATTCAATTCCTAATGTTAGCCCTTCAGAT
  10815
CC[G/A]ATAAGGTTTGGACATGCTGTAGCCCGTGTTTGGGCCTCGTTGTATACTAGAAGAG
                                       10911   10916 10917
CAGTACTGAGCCGCAGAGCTGCTGGTGTGTCCCAGAAAGA[C/T]GCTA[C/A][A/G]ATG
  10923        10932                            10962
GC[C/T]GTGCTAGT[G/T]CAAGAAATGCTTTCACCAGATTTATCTTT[C/T]GTCCTCCA
                                                  11022
CACACTGAGCCCAACGGACAACAATCATAACTTCATTGAGGCTGAAATTGC[A/T]CCTGGA
                                  11064
CTCGGTGAAACACTCGCTTCAGGAACAAGGGGTAC[A/T]CCGTGGCGTCTATCTAGTGGTA
                        11112     11218
AATTTGACGACACAGTGCGCACACT[G/A]GCATT[C/T]GCCAACTTTAGTGAGGAGATG[
11140      11148 11151 11152
G/A]TTGTAGG[T/A]GG[C/T][A/C]ATTCCCCTGCTGACGGAGAAGTTATTCTCTTGACTGTCGATTATAGCAAGAAACC
TTTAACAATCGACCCCATTTTCAGACGTCAGCTTGGTCAGAGGCTTGGCGCTGTTGGTTTCTACCTAGAACGCAAGTTTGGTT
CTCCTCAAGATGTCG
```

3. Beta amylase 1, *BMY1* (PGSC0003DMG400001855)

Chr08:50594548..50595107

```
GCTACTGGAGCATGGTGACAGAATAGTAGCAGCAGGAGAAAGTATATACCAAGGAACTGGGGCTAAACTATCTGGAAAGGTAG
CTGGGATTCATTGGCATTACAATACTAGATCACATGCTGCA
                                     2533
GAGTTAACTTCAGGATATTATAATACAAGACACAGA(G/A)ATGGTTATCTACCTATAGCAC
           2565           2576       2583
GTATGTTAGC(G/A)AAACATGGTG(C/T)TGTACT(T/G)AACTTTACATGTATGGAAATG
         2613            2625                        2649
AGGGATGG(T/G)GAACAGCCCCA(G/A)AGTGCAAACTGTTCACCAGAAGG(C/A)TTAGT
   2657              2671                2686         2694
TC(A/G)ACAAGTTAAAACT(G/A)CAGCTAGAACTGCT(A/G)AAGTAGA(A/G)CTTGCT
                                                   2751
GGAGAAAATGCTCTAGAAAGGTATGATGGAGGAGCATTTTCTCAAGTTTT(G/A)GCAACAA
         2767                                            2811
GCATGTCA(A/G)ATTCTGGAAATGGATTGAGTGCATTTACATTCTTGCGAATGAA(C/T)AAACGGTTGTTTGAGCCAGAAA
ATTGGCGGAATCTAGTGCAATTTGTGAAGAGCATGTCTGAAGGAGGTCGAAATGCTAGCCTTCCAGAGTGTGACTCAAGCAGG
ACAGACCTCTATGTAA
```

4. Starch phosphorylase, chloroplastic/amyloplastic, *PHO1b* (PGSC0003DMG400028382)

Chr05:350160..351337

```
TGTTGCAAGAAAAGCTAAACCAAATGAGGATTCTGGATAATGTTGAAATACCAACTTCTGTTTTGGAGTTGCTTATAAAAGCC
GAAGAAAATGCTGCTGATGTCGAAAAAGCAGCAGATGAAGAACAAGAAGAAGAAGGTAAGGATGACAGTAAAGATGAGGAAAC
TGAGGCTGTAAAGGCAGAAACTACGAACGAAGAGGAGGAAACTGAGGTTAAGAAGGTTGAGGTGGAGGATAGTCAAGCAAAAA
TAAAACGTATATTTGGGCCACATCCAAATAAATCACAGGTGGTTCACATGGCAAATCTATGTGTAGTTAGCGGGCATGCAGTT
AACGGTGTTGCTGAGATTCATAGTGAAATAGTTAAAGATGAAGTTTTCAATGAATTTTACAAGGTACGATGATTATCACACGG
CTAGAAGTGGCAGATATCA

                                                        3882
TTTTCAGTTCTCCTCTTTCAATGTTGTTGTTTTGATTGATCCAAACTCTTTGTTC(A/C)TC
                                              3931   3934
GACAGTTATGGCCAGAGAAATTCCAAAACAAGACAAATGGTGTGAC(A/G)CC(A/T)AGAA
                    3958                        3982      3990
GATGGCTAAGTTTCTGTAA(T/C)CCAGAGTTGAGTGAAATTATAAC(A/C)AAGTGGA(C/
                              4018                    4038
T)AGGATCTGATGATTGGTTAGTAAACAC(T/A)GAAAAATTGGCAGAGCTTC(G/A)AAAG
       4049    4052                        4076           4088
GTAAAC(G/A)AG(T/C/G)TGTATCTGACTGTGATCTTATT(G/A)ATTCCTAAACC(G/A
                   4106   4109     4112          4122
)TCGTCAACAGCTTTCAT(A/G)AA(C/G/A)GG(A/G)TTATTTCCT(T/C)TAGTCCTTC
                                                 4181
TATGTGGAAAGAGATCTTTATAAACATCATAAGAACATGTGAAATGTAT(G/A)TACTAA(C
4188         4198        4206   4207
/T)TTCATGCAT(T/G)TAACTGT(T/C/G)(G/T/A)AACAGTTTGCTGATAACGAAGAAC
     4236            4248          4258
TCCA(G/A)TCTGAGTGGAG(G/A)AAGGCAAAA(G/A)GAAATAACAAAATGAAGATTGTC
                     4302                4319 4320
TCTCTCATTAAAGAAAAAAC(A/T)GGATACGTGGTCAGTC(C/G)(C/T)GATGCAATGTT
                   4350      4356         4365
TGATGTTCAGATCAAGCG(C/T)ATCCA(T/C)GAGTATAA(A/G)AGGCAGCTATTAAATA
  4383      4389               4404   4407      4413
T(A/T)TTTGG(A/T)ATCGTTTATCGCTA(T/C)AA(G/A)AAGAT(G/T)AAAGAAATGA
        4431                             4457                4437
GCCCTGA(A/T)GAACGAAAAGAAAAGTTTGTCCCTC(G/A)AGTTTGCATATTTGG(G/A)GGAAAAGCATTTGCTACATAT
GTTCAGGCCAAGAGAATTGTAAAATTTATCACTGATGTAGGGGCAACAGTCAACCATGATCCCGAGATTGGTGATC
```

5. Soluble starch synthase I, *SssI* (PGSC0003DMG402018552)

Chr03:45608638..45609659

```
GGATACTCATGGGAAATAACAACTCCTGAAGGGGGATATGGGCTACATGAGCTGTTGAGCAGTAGACAGTCTGTTCTTAATGG
TATGCGTGTTAGATGATTCCTTATGAGTGCTTTCTGATATTCTTCTTTTCCTCTTACTCGAATTGTAATTTGGTAGGATATAT
GTTGATTGACGAGGCTTATGTACCAGCATTAGCTTCATTTCTAGTCAAGTCCTAACTTTTGTTAAAACATGACCAGAGCCAAG
GCTAATTGTATTAATGACCTTCACCATGCATTTCTGTTGTCTCTGAACTTTAGAAATTACTAGTTAATCTATCTCTACTTTTA
TGCAGGAATTACTAATGGAATAGATGTTAATGATTGGAACCCGTCGACAGATGAGCATATTGCTTCGCATTACTCCATCAATG
ACCTCTCCGGAAAGGTTATTAAACTTCTCAGTTTCACAATTTTGCAAATTAAGTGATAAAGTGCAGCTAGAGAAAAGGACTAT
TAGCATATTTGGCCAATCTTCCATAAATTGCTTATTTTGGGAAGTGTTTTTTATTAGAAGTACTTTTGGAGAATAGTAGTTTG
TATTTGGCTAATCAATTTGAAAAAAAGCCTTTGTCAATATTAGAGCAGTAATTTGTGCTTGACAAAGGTTCCAATAGTGCTTC
GAGGGGAAAAGCTATTTT
                                         5803
TTTCTTCCAACTTCAGCTACTACTCAAAAGTCAAAAGCAC(indelA)GCTTGGCTGAACAG
       5824               5839             5850        5858
ACAATA(C/T)GTCTACAAAAGCTT(C/A/T)TTAACTGCTG(C/T)GAAATTC(A/G)ATC
          5871     5877   5880
CAAATGAGG(A/G)TTCCT(C/T)TA(G/A)AGGATTGTCCATAATCCATTAAAAGGGAACA
                                                    5964
GTCCAAAGGATATCATAGCATTTACTTACTTTTCATGTTTATTCTACATGCA(G/A)TTGTG
                                6001         6010
CACAAACTCTCTTTTTCTCTGTTCTCTACAT(A/G)CCACTGCT(C/T)AAATTGTCTCTGCTAACTTTCCATTTAATTATAC
AGGTTCAGTGCAAGACTGATCTGCAAAAGGAACTGGGCCTTCCAATTCGACCTGATTG
```

6. Phosphoglucomutase, *PGM-3* (not annotated)

Chr03:41510113..41509054

```
ATGGCTATGGAGAGTGCATTGACATCCACACGAGTTTCAATTCCATCGTTGTGTTCTGGGAT
                               93
CAGTTCATCTCATCATCACCATAGATCCTT[C/A/T]TCATTTCTCAATTTCCCCAAATTGT
       125
CTTCAT(T/G)CAAATACTCTTTTCGAACAATTTCACCTGTTCCTTTCGTTGTCAGTGCATCCTCTGTTTCTCCATCATCTCC
TTCTACATCTGTTGCCCAATCTCAGGATCTCAAGGTATCTA
   241    242             254
AT(T/A/C)(G/A)CTTCCAAATAT(C/T)ACTATATAACGTGTATATTTTTATGTGATTTA
                                  320                    339
ATGGAAAATTTGATTTACTTGAATGCCAGATTA(A/T)ATCAGTTCCTACCAAGCC(T/A)ATTGAAGGTCAAAAGACCGGTA
CCAGTGGTCTACGCAAAAAGGTGCCTTTTCTTTTGGTTTTA
      408  409  410  411   413                          440
TTAAA(A/T)(T/A)(T/A)(T/A)T(A/T)CACTTTGTTTGGATGATTGTTACCCG(C/T)
                           467  468
TGTATTGTATTGTTAGTTCAAATACG(A/G)(T/C)GTTTATTTTGATTGTTATATAAATTTGTTGTTTAGTATCATTTAAAT
TCATTTTCATTGTTAGCTACAAAAAGTCCCATTTTGTGTAAGGACCAATTTGGTGTGATTGCCTTGGTTACTTTTAATATTTT
CTTCTCATTTATCACATTTTAATGTTTTATCCTTTACCCTATTTTTTCGTAGTAGCTACATCCCGTGCCTTACTTTTCTCGTA
GGCTTATCAGACATATCGCAATGACGGAGAATGATACAATCTATCCAAATGTTGTATTTATTAAAATAATATTGTACGATACA
ATACAACACAATATGATACATAATGAAATAATACATAACAGCCCTCCAAGCAGAGTGAACTCCGCCTGCGTGAAGGTGTGCCA
AAATAAATAGGAAAACCTCTCTACCACTTAAGGTAGGGTTAAGGCTGCGTATATTTTACCTTCCTCAGACCTCACTTGTGCGA
TTACACTGGTTTGTTATTGTTGTTGGTTTATTAAATTTTTTATTGCTTACTGTTCATTTGGATGACTTCTGCTTCTTTTGTCT
ATAGGTTAAAGTGTTTATGCAGGACAATTACCTTGCCAATTGGATAC
```

7. ADP-glucose pyrophosphorylase S, *AGPaseS* (PGSC0003DMG400000735)

Chr01: 86095512..86094580

```
GGAGATGGATTTGTCGAGGTTTGAGACTATACTTTTGAAATTCTTGAAGGAAACTGGATTAGTCTTGCTAGATATCACAGCTG
AGCTAATTAATATGAGTAAATTTGTTGGTCCTCCAAGTGAT
                                                1259      1265
TTGTTCACACAAAATCTGTAACAATTTCGGATGATTTTTTTCATTGTT(T/C)TTCTG(A/C
   1268         1276        1284  1286
)TT(A/T)CTTGCTT(C/A)GTTTTAC(T/C)G(A/G)CTGAAAGGAATTCCGTCATTAAAC
                          1336            1348
AGGTACTAGCTGCAACTCAGACACC(C/T)GGGGAAGCAGG(A/G)AAAAAATGGTTTCAAG
                           1392                    1411
GAACAGCAGATGCTGTTAGAAAATTTA(T/C)ATGGGTTTTTGAGGTTTGT(C/T)TCTACA
        1426                              1456 1457 1459
ACTCTTTA(A/G)GACACACTAATCCACCACTGCTCTTCTGT(T/C)(G/)A(C/)CTGATA
                 1482                   1501       1509
TAATTTCTGAAAAATG(A/T)TTCATTATTTTTTCCTGA(T/A)TCAGTTA(G/)TATGTAC
   1520          1529                 1546 1547
AGA(T/C)ATATAGTA(C/T)TTTTGTGTTTACAAGG(T/C)(G/A)ACCAATTCTTGCAGG
  1564                            1592                1612
A(C/T)GCTAAGAACAAGAATATTGAAAATATC(G/C)TTGTACTATCTGGGGATCA(T/C)CTTTATAGGATGGATTATATG
GAGTTGGTGCAGGTATGTCCACTTTAACTACCTATGCACTC
               1689
CTCATTCTTTGTTT(A/G)CTTTTTTTTTTTTTTTTTTTAAACAACAAAGTTTCTGACTGCCTCCGATCTTCAAACAGAACCA
TATTGACAGGAATGCTGATATTACTCTTTCATGTGCACCAGCTGAGGACAGGTTTGTTTAAATTCCAATAAGAAACAACTAGT
AATGGTTAAGTGAATTGAGTGAGATCAGAAACAATATGGAGAAAGAGTGCTTCTCCTTTAATATTCGTATGCATTACTTCGAA
GTAAAAACATCACCACATTTCTTTATACCTTATTGTGATTATGTTAAGCTGAGATTTAAGCTTTACTTATCAGCCGAGCATCA
GATTTTGGGCTGGTCA
```

8. Invertase, *INV-8/2* (PGSC0003DMG400004790)

Chr08:52703547..52704642

```
GTTCTCATCCCACCACCCGGAATTGGGCTCAAGGACTTCCGTGACCCCACCACCGCGTGGACCACACCGGAAGGCAAATGGCG
TATTACTATTGGTTCAAAGATTAATAAAACCGGAATCTCAT
                                      2065         2075  2076
TGGTCTATGACACAATTGATTTTAAAAAATTTGAATT[G/A]TTGAAGGGT[G/A][C/T]G
                                       2116
TTACATGGTGTACCGGGTACGGGTATGTGGGAATGTGT[G/A]GATTTTTACCCGGTTTC[T
2134                                        2175       2182
/G]AAAATTGTTGAAAATGGGCTTGACACTTCAGAAAATGGGC[C/A]TGCAGT[G/A]AAA
           2197
CATGTTTTGAA[G/A]TCCAGTCTTGACGATGATCGAAATGACTATTATGCCCTTGGAACTT
            2255                    2272
ATGATGCAGGG[G/A]CAGGAAAATGGGTACC[T/A]GATAATCCAATAATTGATGTTGGTA
     2302                 2320
TTGG[G/A]TTAAGATATGATTATGG[T/C]AATTTTTATGCATCAAAAACATTTTATGATC
                  2369  2371
AAGAAAAAAAGAGAAGA[G/A]T[T/A]CTTTGGGCTTGGATTAAAGAAACTGATAGTGAAG
                   2425
CTGCTGATATTTGCAGGG[T/C]TTGGGCCTCACTTCAGGTATCTCTTTTTTAGCGTATCATTTAGATTTTGTCCTTATTTTT
AAAAGTCGTGCAAGTAGATACATAATTTGAAGTGTGACAAGTAATCAAAAATCAGACAATGACAGAACGGCCTATGATTAAGA
ACTAAAAACTAAAACTTTATACAACTTTTACAATTCATTGAACAAATTGGTAACGTGTGATTTTTTATTATTTTATTTCGAAA
ACAGTCAATTCCAAGAACTATAAAGTATGATAAGAAGACAGGAAGCAACATAATTACATGGCCAGTGGCTGAGGTAGAGAATT
TGAGATCAAACAACAATGAATTCAACAAGGTTGTAGTTAAACCAGGTTCAATTGTCCCACTAGAAGTTGGTTCTGCCACTCAG
GTTAGTACATTTTGTCGATTAAATTCAACTTATATACATTGACAATATATATACTTATATCGTTGAATTATTGTTACAGTTGG
ACATAATGGCTGAATTTGAAATAGACCAAAATGTGTTGAAGAAAGTAGATGGAAGTAATGCTACATATGATTGTATAAAGAGT
GGTGGATCTGGTGAACG
```

File S2

Pair wise linkage disequilibrium (LD) between SNP markers scored in the CHIPS-ALL population.

Available for download as an Excel file at http://www.g3journal.org/lookup/suppl/doi:10.1534/g3.114.012377/-/DC1

Table S1 Genes functional in plant starch-sugar interconversion as annotated in the potato genome and GeneBank accessions of corresponding cloned and characterized genes from potato and/or tomato. Loci highlighted green have been probed for association with tuber quality traits (this paper, BALDWIN *et al*. 2011, DRAFFEHN *et al*. 2010, KAWCHUK *et al*. 2008, LI *et al*. 2008, LI *et al*. 2005, LI *et al*. 2013, URBANY *et al*. 2011).

Enzyme/protein	Locus acronym [a]	Locus ID	GenBank accessions	Position (v4.03)
ADP-glucose pyrophosphorylase, large subunit	*AGPaseS-1/1*	PGSC0003DMG400009026	NM_001247048	chr01:62144770..62149140
ADP-glucose pyrophosphorylase, large subunit	*AGPaseS-1/2*	PGSC0003DMG400000735	X61187	chr01:86092270..86097270
ADP-glucose pyrophosphorylase, large subunit	*AGPaseS-7*	PGSC0003DMG400015952	NM_001247018	chr07:21365000..21370700
ADP-glucose pyrophosphorylase, small subunit	*AGPaseB-7*	PGSC0003DMG400031084	NM_001288195	chr07:51451500..51457400
ADP-glucose pyrophosphorylase, small subunit	*AGPaseB-12*	PGSC0003DMG400046891 [c]		chr12:1226600..1230220
1,4-alpha-glucan-maltohydrolase	*AMY-1*	PGSC0003DMG400010664	AJ849377	chr01:59361270..59364450
Alpha-amylase	*AMY-3*	PGSC0003DMG400020603	A21347	chr03:35999180..36002230
Alpha-amylase, AMY21	*AMY-4/1*	PGSC0003DMG400007974	M81682	chr04:68256940..68260200
Alpha-amylase, AmyZ	*AMY-4/2*	PGSC0003DMG400009891	M79328	chr04:71332570..71337470
Alpha-amylase	*AMY-5*	PGSC0003DMG401017626		chr05:5918690..5923240
Beta-amylase	*BMY-1*	PGSC0003DMG400000169		chr01:72104900..72114100
Beta-amylase	*BMY-7/1*	PGSC0003DMG400026199		chr07:47346060..47350380
Beta-amylase	*BMY-7/2*	PGSC0003DMG400026166		chr07:47402480..47403710
Beta-amylase	*BMY-7/3*	PGSC0003DMG400026198 [d]		chr07:47419377..47419678
Beta-amylase,PCT-BMYI	*BMY-8/1*	PGSC0003DMG402020509		chr08:1075900..1079370
Beta-amylase, plastidic, PCT-BMYI	*BMY-8/2*	PGSC0003DMG400001855	AF393847	chr08:50592100..50595690
Beta-amylase, BMYI	*BMY-8/3*	PGSC0003DMG400012129		chr08:56381800..56388400
Beta-amylase	*BMY-9*	PGSC0003DMG400001549	BD247483	chr09:57420530..57423450
Debranching enzyme, isoamylase isoform 3	*DBE-6/1*	PGSC0003DMG402007274	BD080224	chr06:368200..374700
Debranching enzyme, isoamylase isoform 3	*DBE-6/2*	PGSC0003DMG401007274	BD080224	chr06:375900..383000
Debranching enzyme, isoamylase isoform 1	*DBE-7*	PGSC0003DMG400020699	BD080223, A93395	chr07:7890210..7894280
Debranching enzyme, isoamylase isoform 2	*DBE-9*	PGSC0003DMG400000954	AY132997, BD080222	chr09:46684830..46688580

Debranching enzyme, isoamylase isoform 1	*DBE-10*	PGSC0003DMG400030253		chr10:38081140..38083210
Debranching enzyme	*DBE-11*	Not annotated	A52190	chr11:3945240..3949550
Disproportionating enzyme, 4-alpha-glucanotransferase	*DPE-2*	Not annotated	AY510449	chr02:5166700..5174600
Disproportionating enzyme, 4-alpha glucanotransferase, plastidic	*DPE-4*	PGSC0003DMG400016589	X68664	chr04:53993900..54001200
Fructokinase, StFrk3	*FRK-2*	PGSC0003DMG400030653	JX839755	chr02:41888630..41893510
Fructokinase, StFrk1	*FRK-3*	PGSC0003DMG400024246	U64817	chr03:41525810..41530530
Fructokinase	*FRK-5*	PGSC0003DMG400028311		chr05:687340..692010
Fructokinase	*FRK-6/1*	PGSC0003DMG400027017		chr06:53746100..53749420
Fructokinase, StFrk2	*FRK-6/2*	PGSC0003DMG400026916	JX576230, Z12823, U64818	chr06:53749700..53752940
Fructokinase	*FRK-9*	PGSC0003DMG400020361	NM_001288518	chr09:707190..710110
Alpha-glucan water dikinase	*GWD*	PGSC0003DMG400007677	Y09533	chr05:9823400..9839000
Glucose-6-phosphate/phosphate translocator, plastidic	*G6PPT-0*	PGSC0003DMG400005602		chr00:38861730..38863900 (DMB0000017769)
Glucose-6-phosphate/phosphate translocator, plastid	*G6PPT-1*	PGSC0003DMG400044320		chr01:67459560..67460820
Glucose-6-phosphate/phosphate translocator, plastid	*G6PPT-4*	PGSC0003DMG402012710		chr04:10972680..10976630
Glucose-6-phosphate/phosphate translocator, plastid	*G6PPT-5*	PGSC0003DMG400005269	AY163867, AF020816	chr05:42300210..42303690
Glucose-6-phosphate/phosphate translocator	*G6PPT-7/1*	PGSC0003DMG400025495		chr07:47074660..47077700
Glucose-6-phosphate/phosphate translocator, plastidic	*G6PPT-7/2*	PGSC0003DMG400001041		chr07:54548020..54553010
Hexokinase	*HXK-2*	PGSC0003DMG400030624		chr02:42162900..42168200
Hexokinase	*HXK-3*	PGSC0003DMG400002525	X94302	chr03:60521600..60527100
Hexokinase, plastidic	*HXK-4*	PGSC0003DMG400009861	NM_001288386, AF118134	chr04:70783660..70787820
Hexokinase	*HXK-6*	PGSC0003DMG400016521	NM_001287912	chr06:49126980..49131660
Hexokinase	*HXK-11*	PGSC0003DMG400013187		chr11:36684900..36690400
Hexokinase	*HXK-12*	PGSC0003DMG400000295	DQ056861	chr12:4039600..4049300
Hexose transporter	*HT-1*	PGSC0003DMG400045929		chr01:63002770..63005240
Hexose transporter	*HT-2/1*	PGSC0003DMG400022402	AJ132223, NM_001247920	chr02:34969850..34973440

Hexose transporter, plastidic	*HT-2/2*	PGSC0003DMG400026402	AF215853	chr02:41402000..41411600
Hexose transporter	*HT-4/1*	PGSC0003DMG400025054 [c]		chr04:61467750..61469290
Hexose transporter	*HT-4/2*	PGSC0003DMG402006401		chr04:63373150..63377240
Hexose transporter	*HT-4/3*	PGSC0003DMG400009994		chr04:71865900..71871700
Hexose transporter	*HT-7*	PGSC0003DMG402018758	AJ132225, NM_001247926	chr07:2681670..2686150
Hexose transporter	*HT-9*	PGSC0003DMG400031832	AJ132224, NM_001247856	chr09:53280800..53287100
Invertase, beta-fructofuranosidase, neutral	*INV-n-1/1*	PGSC0003DMG400013088		chr01:37067200..37072500
Invertase, beta-fructofuranosidase	*INV-1/2*	PGSC0003DMG400002756		chr01:78430100..78436100
Invertase, beta-fructofuranosidase	*INV-1/3*	PGSC0003DMG400001596		chr01:87160540..87164480
Invertase, beta-fructofuranosidase, soluble acid	*INV_Pain-1*	PGSC0003DMG400013856	X70368, NM_001288064	chr03:39255060..39259540
Invertase, beta-fructofuranosidase, cell wall	*INV-cw-3*	PGSC0003DMG400002583		chr03:61076900..61079820
Invertase, beta-fructofuranosidase	*INV-4*	PGSC0003DMG400009936	JX576233	chr04:70804760..70809220
Invertase, beta-fructofuranosidase, neutral /alkaline	*INV-na-6/1*	PGSC0003DMG400026107		chr06:46660300..46665800
Invertase, beta-fructofuranosidase, cell wall	*INV-6/2*	PGSC0003DMG400033142		chr06:47626000..47633600
Invertase, beta-fructofuranosidase, cell wall	*INV-cw-8/1*	PGSC0003DMG400027925		chr08:27921320..27925690
Invertase, beta-fructofuranosidase	*INV-8/2*	PGSC0003DMG400004790	NM_001247140	chr08:52701630..52705710
Invertase, beta-fructofuranosidase, cell wall	*InvGE*	PGSC0003DMG400008942	AJ133765, NM_001287894	chr09:2469540..2471550
Invertase, beta-fructofuranosidase, cell wall	*InvGF*	PGSC0003DMG400008943	AJ133765, NM_001288126	chr09:2474700..2477140
Invertase, beta-fructofuranosidase, cell wall	*INV-cw-10/1*	PGSC0003DMG400046915 [c]		chr10:15877770..15881930
Invertase, beta-fructofuranosidase	*INV-10/2*	PGSC0003DMG400008388		chr10:53235940..53238050
Invertase, beta-fructofuranosidase	*INV-10/3*	PGSC0003DMG400011037		chr10:53706680..53710620
Invertase, beta-fructofuranosidase, cell wall	*InvCD111*	PGSC0003DMG401028252	X95820, NM_001287886	chr10:55840810..55843010
Invertase, beta-fructofuranosidase, cell wall	*InvCD141*	PGSC0003DMG402028252	X95821, NM_001288398	chr10:55851950..55856800
Invertase, beta-fructofuranosidase	*INV-11/1*	PGSC0003DMG400019494		chr11:5046800..5053000
Invertase, beta-fructofuranosidase, neutral /alkaline	*INV-na-11/2*	PGSC0003DMG400009257		chr11:13676200..13683400
Invertase, beta-fructofuranosidase, neutral	*INV-n-11/3*	PGSC0003DMG400026530		chr11:39907600..39913800
Invertase/pectin methylesterase inhibitor family protein	*InvInh-3/1*	PGSC0003DMG400045232 [c]		chr03:39515876..39516269

Invertase inhibitor, Kunitz-type	*KT-InvInh-3/2*	PGSC0003DMG400010146	AF459077, AY594178, KC184127	chr03:49448370..49449150
Invertase inhibitor	*InvInh-5*	PGSC0003DMG400039425 [c]		chr05:42860389..42860866
Invertase inhibitor	*InvInh-8*	PGSC0003DMG400001844		chr08:50229740..50233130
Invertase/pectin methylesterase inhibitor family protein	*InvInh-10/1*	PGSC0003DMG400001276 [c]		chr10:22401030..22401750
Invertase inhibitor	*InvInh-10/2*	PGSC0003DMG400037656 [c]		chr10:50545200..50545720
Invertase inhibitor	*InvInh-10/3*	PGSC0003DMG400040471 [c]		chr10:51072890..51073410
Invertase inhibitor	*InvInh-10/4*	PGSC0003DMG400037039 [c]		chr10:59551460..59551980
Invertase inhibitor	*InvInh-11*	PGSC0003DMG400038811 [c]		chr11:7433850..7434380
Invertase inhibitor	*InvInh-12/1*	PGSC0003DMG400004616	KC007445, GU980595	chr12:59956850..59957600
Invertase inhibitor	*InvInh-12/2*	PGSC0003DMG400004617	NM_001247862, AY864820	chr12:59963540..59965500
Invertase inhibitor	*InvInh-12/3*	PGSC0003DMG400004618	GU980592	chr12:59967330..59968500
Maltose transporter	*MT*	PGSC0003DMG400024812		chr04:58261800..58269200
Glucose-6-phosphate isomerase	*PGI-0*	PGSC0003DMG400009848		chr00:32605770..32609090 (PGSC0003DMB000001156)
Glucose-6-phosphate isomerase	*PGI-1*	PGSC0003DMG400030128		chr01:20860570..20862590
Glucose-6-phosphate isomerase	*PGI-12*	PGSC0003DMG400015341 [d]	DQ451687	chr12:147900..160900
Phosphoglucomutase, plastidic	*PGM-0*	PGSC0003DMG400001912		chr00:12338800..12351200 (PGSC0003DMB000000431)
Phosphoglucomutase, plastidic, PGM1	*PGM-3*	Not annotated	AJ240053	chr03:41493531..41510113
Phosphoglucomutase, cytosolic	*PGM-4*	Not annotated	AJ240054	chr04:35685500..35706600
Phosphoglucomutase, plastidic	*PGM-8*	PGSC0003DMG400024224		chr08:50101900..50108200
Alpha-glucan phosphorylase, L-type, plastidic, Stp23	*PHO1a*	PGSC0003DMG400033858+ PGSC0003DMG400003495+ PGSC0003DMG400007782+ PGSC0003DMG400002479	D00520, X52385	chr03 (Chen et al. 2001), not on physical map
Alpha-glucan phosphorylase, L-type, plastidic, StpL	*PHO1b*	PGSC0003DMG400028382	X73684, BD056944	chr05:346700..352900

Alpha-glucan phosphorylase, H-type, cytosolic, StpH	*PHO2*	Not annotated	M69038, NM_001288189, L13771	chr09 (Chen et al. 2001), not on physical map (PGSC0003DMB000000644)
Phosphoglucan water dikinase	*PWD*	PGSC0003DMG400016613	AY747068	chr09:60560500..60572400
Starch branching enzyme I	*SBE I*	PGSC0003DMG400009981	X08786	chr04:71587800..71595800
Starch branching enzyme III	*SBE III*	PGSC0003DMG400022307		chr07:55034530..55039270
Starch branching enzyme II	*SBE II*	Not annotated	AJ000004	chr09:3737100..3755900
Phosphoglucan phosphatase (At3g52180)	*SEX4*	PGSC0003DMG400015246		chr03:50875700..50885600
Sucrose phosphate synthase	*SPS-7*	PGSC0003DMG400027936	X73477	chr07:3888900..3897800
Sucrose phosphate synthase	*SPS-8*	PGSC0003DMG400029892	JQ086765	chr08:20206590..20210220
Sucrose phosphate synthase isoform B	*SPS-9*	PGSC0003DMG400026428	JQ086766	chr09:59231200..59240200
Starch synthase IV	*SS IV*	PGSC0003DMG400008322	GQ221264	chr02:30142700..30152300
Starch synthase, soluble, plastidic	*Sss III*	PGSC0003DMG400016481	NM_001287873	chr02:36377150..36379320
Starch synthase V	*SS V*	PGSC0003DMG400030619	EU661369	chr02:42100900..42107600
Granule bound starch synthase II, plastidic, SSII	*GbssII*	PGSC0003DMG400001328	NM_001288048	chr02:46189600..46196700
Starch synthase, soluble	*SssI*	PGSC0003DMG402018552 [b]	NM_001288145	chr03:45887500..45896600
Starch synthase VI	*SS VI-7/1*	PGSC0003DMG401013540		chr07:40921600..40929800
Starch synthase VI	*SS VI-7/2*	PGSC0003DMG402013540		chr07:40932600..40939900
Granule bound starch synthase I, plastidic, waxy	*GbssI*	PGSC0003DMG400012111	X58453	chr08:56782190..56785790
Sucrose synthase	*SUS-2*	PGSC0003DMG400016730		chr02:36937430..36941850
Sucrose synthase	*SUS-3*	PGSC0003DMG400031046		chr03:48949000..48950010
Sucrose synthase	*SUS-7/1*	PGSC0003DMG400013547		chr07:40610340..40613980
Sucrose synthase, Sus3	*SUS-7/2*	PGSC0003DMG400013546	U24088, AY205084	chr07:40638500..40644900
Sucrose synthase	*SUS-9*	PGSC0003DMG400006672	AY205302	chr09:61464900..61472300
Sucrose synthase, Sus4	*SUS-12*	PGSC0003DMG400002895	U24087, DQ793135	chr12:3085100..3091500
Sucrose transporter	*Sut2*	PGSC0003DMG400025610	NM_001288509, AY291289	chr05:5692340..5694730
Sucrose transporter	*Sut1*	PGSC0003DMG400009213	X69165	chr11:9052430..9057350

Sucrose transporter	*Sut4*	Not annotated	NM_001288141, AF237780	chr04:65861800..65873100
UDP-glucose pyrophosphorylase	*UGPase-1*	PGSC0003DMG401031123		chr01:67210700..67220100
UDP-glucose pyrophosphorylase	*UGPase-11*	PGSC0003DMG401013333	D00667, NM_001288019	chr11:808300..814800
		Total 123	Total 66	

[a] Loci without established names from the literature are distinguished based on chromosomal location, loci tested for association are highlighted green.

[b] Not annotated in genome browser v4.03

[c] Very low or no expression, pseudogene?

[d] Only small gene fragment, incomplete annotation

Table S2 Correspondence between presence (1) or absence (0) of the Stp23-8b SSCP marker and PHO1a cDNA SNPs in 34 standard varieties of the CHIPS-ALL population (Li *et al*. 2008).

Variety	Stp23-8b	$C_{22}T$	$G_{322}A$	G_{824}	A_{2776}
Leyla	0	CCCC	GGGG	0	0
Marabel	0	CCCC	GGGG	0	0
Solara	0	CCCC	GGGG	0	0
Vitara	0	CCCC	GGGG	0	0
Milva	0	CCCC	GGGG	0	0
Ponto	1	TCCC	AGGG	1	1
Tomensa	1	TCCC	AGGG	1	1
Marlen	1	TCCC	AGGG	1	1
Eldena	1	TCCC	AGGG	1	1
Theresa	1	TCCC	AGGG	1	1
Goldika	1	TCCC	AGGG	1	1
Saturna	1	TTCC	AAGG	1	1
Karlena	0	CCCC	GGGG	0	0
Kolibri	0	CCCC	GGGG	0	0
Fasan	0	CCCC	GGGG	0	0
Terra	0	TCCC	AGGG	1	1
Solist	0	CCCC	GGGG	0	0
Melina	1	TCCC	AGGG	1	1
Molli	0	CCCC	GGGG	0	0
Likaria	0	CCCC	GGGG	0	0
Valisa	0	CCCC	GGGG	0	0
Apart	0	CCCC	GGGG	0	0
Artis	0	CCCC	GGGG	0	0
Aula	0	CCCC	GGGG	0	0
Christa	0	CCCC	GGGG	0	0
Diana	1	TCCC	AGGG	1	1
Novara	0	CCCC	GGGG	0	0
Ilona	0	CCCC	GGGG	0	0
Orlando	0	CCCC	GGGG	0	0
Panda	1	TCCC	AGGG	1	1
Satina	0	CCCC	GGGG	0	0
Sempra	0	CCCC	GGGG	0	0
Sirius	1	TCCC	AGGG	1	1
Velox	0	CCCC	GGGG	0	0
