# Supplementary material for: SNPs in Genes Functional in Starch-Sugar Interconversion Associate with Natural Variation of Tuber Starch and Sugar Content of Potato (Solanum tuberosum L.)
Source: G3 (Bethesda). 2014 Jul 31;4(10):1797–811. doi: 10.1534/g3.114.012377 (PMC4199688; doi:10.1534/g3.114.012377)
Supplement: Supporting Information [file supp_g3.114.012377_FigureS1.pdf]

```
                  *        20         *        40         *        60         *        80         *       100
Pho1a-HR : ATGGCGACTGCAAATGGAGCACACTTGTTCAACCATTACAGCTCCAATTCCAGATTCATCCATTTCACTTCTAGAAACACAAGCTCCAAATTGTTCCTTA :  100
Pho1a(2) : ATGGCGACTGCAAATGGAGCATACTTGTTCAACCATTACAGCTCCAATTCCAGATTCATCCATTTCACTTCTAGAAACACAAGCTCCAAATTGTTCCTTA :  100
Pho1a(3) : ATGGCGACTGCAAATGGAGCATACTTGTTCAACCATTACAGCTCCAATTCCAGATTCATCCATTTCACTTCTAGAAACACAAGCTCCAAATTGTTCCTTA :  100
Pho1a(4) : ATGGCGACTGCAAATGGAGCATACTTGTTCAACCATTACAGCTCCAATTCCAGATTCATCCATTTCACTTCTAGAAACACAAGCTCCAAATTGTTCCTTA :  100
Pho1a-HA : ATGGCGACTGCAAATGGAGCATACTTGTTCAACCATTACAGCTCCAATTCCAGATTCATCCATTTCACTTCTAGAAACACAAGCTCCAAATTGTTCCTTA :  100
Pho1a(6) : ATGGCGACTGCAAATGGAGCATACTTGTTCAACCATTACAGCTCCAATTCCAGATTCATCCATTTCACTTCTAGAAACACAAGCTCCAAATTGTTCCTTA :  100
Pho1a(7) : ATGGCGACTGCAAATGGAGCACACTTGTTCAACCATTACAGCTCCAATTCCAGATTCATCCATTTCACTTCTAGAAACACAAGCTCCAAATTGTTCCTTA :  100
Pho1a(8) : ATGGCGACTGCAAATGGAGCACACTTGTTCAACCATTACAGCTCCAATTCCAGATTCATCCATTTCACTTCTAGAAACACAAGCTCCAAATTGTTCCTTA :  100
Pho1a(9) : ATGGCGACTGCAAATGGAGCACACTTGTTCAACCATTACAGCTCCAATTCCAGATTCATCCATTTCACTTCTAGAAACACAAGCTCCAAATTGTTCCTTA :  100

                  *       120         *       140         *       160         *       180         *       200
Pho1a-HR : CCAAAACCTCCCATTTTCGGAGACCCAAACGCTGTTTCCATGTCAACAATACCTTGAGTGAGAAAATTCACCATCCCATTACTGAACAAGGTGGTGAGAG :  200
Pho1a(2) : CCAAAACCTCCCATTTTCGGAGACCCAAACGCTGTTTCCATGTCAACAATACCTTGAGTGAGAAAATTCACCATCCCATTACTGAACAAGGTGGTGAGAG :  200
Pho1a(3) : CCAAAACCTCCCATTTTCGGAGACCCAAACGCTGTTTCCATGTCAACAATACCTTGAGTGAGAAAATTCACCATCCCATTACTGAACAAGGTGGTGAGAG :  200
Pho1a(4) : CCAAAACCTCCCATTTTCGGAGACCCAAACGCTGTTTCCATGTCAACAATACCTTGAGTGAGAAAATTCACCATCCCATTACTGAACAAGGTGGTGAGAG :  200
Pho1a-HA : CCAAAACCTCCCATTTTCGGAGACCCAAACGCTGTTTCCATGTCAACAATACCTTGAGTGAGAAAATTCACCATCCCATTACTGAACAAGGTGGTGAGAG :  200
Pho1a(6) : CCAAAACCTCCCATTTTCGGAGACCCAAACGCTGTTTCCATGTCAACAATACCTTGAGTGAGAAAATTCACCATCCCATTACTGAACAAGGTGGTGAGAG :  200
Pho1a(7) : CCAAAACCTCCCATTTTCGGAGACCCAAACGCTGTTTCCATGTCAACAATACCTTGAGTGAGAAAATTCACCATCCCATTACTGAACAAGGTGGTGAGAG :  200
Pho1a(8) : CCAAAACCTCCCATTTTCGGAGACCCAAACGCTGTTTCCATGTCAACAATACCTTGAGTGAGAAAATTCACCATCCCATTACTGAACAAGGTGGTGAGAG :  200
Pho1a(9) : CCAAAACCTCCCATTTTCGGAGACCCAAACGCTGTTTCCATGTCAACAATACCTTGAGTGAGAAAATTCACCATCCCATTACTGAACAAGGTGGTGAGAG :  200

                  *       220         *       240         *       260         *       280         *       300
Pho1a-HR : CGACCTGAGTTCTTTTGCTCCTGATGCCGCATCTATTACCTCAAGTATCAAATACCATGCAGAATTCACACCTGTATTCTCTCCTGAAAGGTTTGAGCTC :  300
Pho1a(2) : CGACCTGAGTTCTTTTGCTCCTGATGCCGCATCTATTACCTCAAGTATCAAATACCATGCAGAATTCACACCTGTATTCTCTCCTGAAAGGTTTGAGCTC :  300
Pho1a(3) : CGACCTGAGTTCTTTTGCTCCTGATGCCGCATCTATTACCTCAAGTATCAAATACCATGCAGAATTCACACCTGTATTCTCTCCTGAAAGGTTTGAGCTC :  300
Pho1a(4) : CGACCTGAGTTCTTTTGCTCCTGATGCCGCATCTATTACCTCAAGTATCAAATACCATGCAGAATTCACACCTGTATTCTCTCCTGAAAGGTTTGAGCTC :  300
Pho1a-HA : CGACCTGAGTTCTTTTGCTCCTGATGCCGCATCTATTACCTCAAGTATCAAATACCATGCAGAATTCACACCTGTATTCTCTCCTGAAAGGTTTGAGCTC :  300
Pho1a(6) : CGACCTGAGTTCTTTTGCTCCTGATGCCGCATCTATTACCTCAAGTATCAAATACCATGCAGAATTCACACCTGTATTCTCTCCTGAAAGGTTTGAGCTC :  300
Pho1a(7) : CGACCTGAGTTCTTTTGCTCCTGATGCCGCATCTATTACCTCAAGTATCAAATACCATGCAGAATTCACACCTGTATTCTCTCCTGAAAGGTTTGAGCTC :  300
Pho1a(8) : CGACCTGAGTTCTTTTGCTCCTGATGCCGCATCTATTACCTCAAGTATCAAATACCATGCAGAATTCACACCTGTATTCTCTCCTGAAAGGTTTGAGCTC :  300
Pho1a(9) : CGACCTGAGTTCTTTTGCTCCTGATGCCGCATCTATTACCTCAAGTATCAAATACCATGCAGAATTCACACCTGTATTCTCTCCTGAAAGGTTTGAGCTC :  300

                  *       320         *       340         *       360         *       380         *       400
Pho1a-HR : CCTAAGGCATTCTTTGCAACAGCTCAAAGTGTTCGTGATTCGCTCCTTATTAATTGGAATGCTACGTATGATATTTATGAAAAGCTGAACATGAAGCAAG :  400
Pho1a(2) : CCTAAGGCATTCTTTGCAACAACTCAAAGTGTTCGTGATTCGCTCCTTATTAATTGGAATGCTACGTATGATATTTATGAAAAGCTGAACATGAAGCAAG :  400
Pho1a(3) : CCTAAGGCATTCTTTGCAACAACTCAAAGTGTTCGTGATTCGCTCCTTATTAATTGGAATGCTACGTATGATATTTATGAAAAGCTGAACATGAAGCAAG :  400
Pho1a(4) : CCTAAGGCATTCTTTGCAACAACTCAAAGTGTTCGTGATTCGCTCCTTATTAATTGGAATGCTACGTATGATATTTATGAAAAGCTGAACATGAAGCAAG :  400
Pho1a-HA : CCTAAGGCATTCTTTGCAACAACTCAAAGTGTTCGTGATTCGCTCCTTATTAATTGGAATGCTACGTATGATATTTATGAAAAGCTGAACATGAAGCAAG :  400
Pho1a(6) : CCTAAGGCATTCTTTGCAACAACTCAAAGTGTTCGTGATTCGCTCCTTATTAATTGGAATGCTACGTATGATATTTATGAAAAGCTGAACATGAAGCAAG :  400
Pho1a(7) : CCTAAGGCATTCTTTGCAACAGCTCAAAGTGTTCGTGATTCGCTCCTTATTAATTGGAATGCTACGTATGATATTTATGAAAAGCTGAACATGAAGCAAG :  400
Pho1a(8) : CCTAAGGCATTCTTTGCAACAGCTCAAAGTGTTCGTGATTCGCTCCTTATTAATTGGAATGCTACGTATGATATTTATGAAAAGCTGAACATGAAGCAAG :  400
Pho1a(9) : CCTAAGGCATTCTTTGCAACAGCTCAAAGTGTTCGTGATTCGCTCCTTATTAATTGGAATGCTACGTATGATATTTATGAAAAGCTGAACATGAAGCAAG :  400

                  *       420         *       440         *       460         *       480         *       500
Pho1a-HR : CGTACTATCTATCCATGGAATTTCTGCAGGGTAGAGCATTGTTAAATGCAATTGGTAATCTGGAGCTTACTGGTGCATTTGCGGAAGCTTTGAAAAACCT :  500
Pho1a(2) : CGTACTATCTATCCATGGAATTTCTGCAGGGTAGAGCATTGTTAAATGCAATTGGTAATCTGGAGCTTACTGGTGCATTTGCGGAAGCTTTGAAAAACCT :  500
Pho1a(3) : CGTACTATCTATCCATGGAATTTCTGCAGGGTAGAGCATTGTTAAATGCAATTGGTAATCTGGAGCTTACTGGTGCATTTGCGGAAGCTTTGAAAAACCT :  500
Pho1a(4) : CGTACTATCTATCCATGGAATTTCTGCAGGGTAGAGCATTGTTAAATGCAATTGGTAATCTGGAGCTTACTGGTGCATTTGCGGAAGCTTTGAAAAACCT :  500
Pho1a-HA : CGTACTATCTATCCATGGAATTTCTGCAGGGTAGAGCATTGTTAAATGCAATTGGTAATCTGGAGCTTACTGGTGCATTTGCGGAAGCTTTGAAAAACCT :  500
Pho1a(6) : CGTACTATCTATCCATGGAATTTCTGCAGGGTAGAGCATTGTTAAATGCAATTGGTAATCTGGAGCTTACTGGTGCATTTGCGGAAGCTTTGAAAAACCT :  500
Pho1a(7) : CGTACTATCTATCCATGGAATTTCTGCAGGGTAGAGCATTGTTAAATGCAATTGGTAATCTGGAGCTTACTGGTGCATTTGCGGAAGCTTTGAAAAACCT :  500
Pho1a(8) : CGTACTATCTATCCATGGAATTTCTGCAGGGTAGAGCATTGTTAAATGCAATTGGTAATCTGGAGCTTACTGGTGCATTTGCGGAAGCTTTGAAAAACCT :  500
Pho1a(9) : CGTACTATCTATCCATGGAATTTCTGCAGGGTAGAGCATTGTTAAATGCAATTGGTAATCTGGAGCTTACTGGTGCATTTGCGGAAGCTTTGAAAAACCT :  500

                  *       520         *       540         *       560         *       580         *       600
Pho1a-HR : TGGCCACAATCTAGAAAATGTGGCTTCTCAGGAACCAGATGCTGCTCTTGGAAATGGGGGTTTGGGACGGCTTGCTTCCTGTTTTCTGGACTCTTTGGCA :  600
Pho1a(2) : TGGCCACAATCTAGAAAATGTGGCTTCTCAGGAGCCAGATGCTGCTCTTGGAAATGGGGGTTTGGGACGGCTTGCTTCCTGTTTTCTGGACTCTTTGGCA :  600
Pho1a(3) : TGGCCACAATCTAGAAAATGTGGCTTCTCAGGAGCCAGATGCTGCTCTTGGAAATGGGGGTTTGGGACGGCTTGCTTCCTGTTTTCTGGACTCTTTGGCA :  600
Pho1a(4) : TGGCCACAATCTAGAAAATGTGGCTTCTCAGGAGCCAGATGCTGCTCTTGGAAATGGGGGTTTGGGACGGCTTGCTTCCTGTTTTCTGGACTCTTTGGCA :  600
Pho1a-HA : TGGCCACAATCTAGAAAATGTGGCTTCTCAGGAGCCAGATGCTGCTCTTGGAAATGGGGGTTTGGGACGGCTTGCTTCCTGTTTTCTGGACTCTTTGGCA :  600
Pho1a(6) : TGGCCACAATCTAGAAAATGTGGCTTCTCAGGAGCCAGATGCTGCTCTTGGAAATGGGGGTTTGGGACGGCTTGCTTCCTGTTTTCTGGACTCTTTGGCA :  600
Pho1a(7) : TGGTCACAATCTAGAAAATGTGGCTTCTCAGGAGCCAGATGCTGCTCTTGGAAATGGGGGTTTGGGACGGCTTGCTTCCTGTTTTCTGGACTCTTTGGCA :  600
Pho1a(8) : TGGTCACAATCTAGAAAATGTGGCTTCTCAGGAGCCAGATGCTGCTCTTGGAAATGGGGGTTTGGGACGGCTTGCTTCCTGTTTTCTGGACTCTTTGGCA :  600
Pho1a(9) : TGGTCACAATCTAGAAAATGTGGCTTCTCAGGAGCCAGATGCTGCTCTTGGAAATGGGGGTTTGGGACGGCTTGCTTCCTGTTTTCTGGACTCTTTGGCA :  600

                  *       620         *       640         *       660         *       680         *       700
Pho1a-HR : ACACTAAACTACCCAGCATGGGGCTATGGACTTAGGTACAAGTATGGTTTATTTAAGCAACGGATTACAAAAGATGGTCAGGAGGAGGTGGCTGAAGATT :  700
Pho1a(2) : ACACTAAACTACCCAGCATGGGGCTATGGACTTAGGTACAAGTATGGTTTATTTAAGCAACGGATTACAAAAGATGGTCAGGAGGAGGTGGCTGAAGATT :  700
Pho1a(3) : ACACTAAACTACCCAGCATGGGGCTATGGACTTAGGTACAAGTATGGTTTATTTAAGCAACGGATTACAAAAGATGGTCAGGAGGAGGTGGCTGAAGATT :  700
Pho1a(4) : ACACTAAACTACCCAGCATGGGGCTATGGACTTAGGTACAAGTATGGTTTATTTAAGCAACGGATTACAAAAGATGGTCAGGAGGAGGTGGCTGAAGATT :  700
Pho1a-HA : ACACTAAACTACCCAGCATGGGGCTATGGACTTAGGTACAAGTATGGTTTATTTAAGCAACGGATTACAAAAGATGGTCAGGAGGAGGTGGCTGAAGATT :  700
Pho1a(6) : ACACTAAACTACCCAGCATGGGGCTATGGACTTAGGTACAAGTATGGTTTATTTAAGCAACGGATTACAAAAGATGGTCAGGAGGAGGTGGCTGAAGATT :  700
Pho1a(7) : ACACTAAACTACCCAGCATGGGGCTATGGACTTAGGTACAAGTATGGTTTATTTAAGCAACGGATTACAAAAGATGGTCAGGAGGAGGTGGCTGAAGATT :  700
Pho1a(8) : ACACTAAACTACCCAGCATGGGGCTATGGACTTAGGTACAAGTATGGTTTATTTAAGCAACGGATTACAAAAGATGGTCAGGAGGAGGTGGCTGAAGATT :  700
Pho1a(9) : ACACTAAACTACCCAGCATGGGGCTATGGACTTAGGTACAAGTATGGTTTATTTAAGCAACGGATTACAAAAGATGGTCAGGAGGAGGTGGCTGAAGATT :  700

                  *       720         *       740         *       760         *       780         *       800
Pho1a-HR : GGCTTGAAATTGGCAGTCCATGGGAAGTTGTGAGGAATGATGTTTCATATCCTATCAAATTCTATGGAAAAGTCTCTACAGGATCAGATGGAAAGAGGTA :  800
Pho1a(2) : GGCTTGAAATTGGCAGTCCCTGGGAAGTTGTGAGGAATGATGTTTCATATCCTATCAAATTCTATGGAAAAGTCTCTACAGGATCAGATGGAAAGAGGTA :  800
Pho1a(3) : GGCTTGAAATTGGCAGTCCCTGGGAAGTTGTGAGGAATGATGTTTCATATCCTATCAAATTCTATGGAAAAGTCTCTACAGGATCAGATGGAAAGAGGTA :  800
Pho1a(4) : GGCTTGAAATTGGCAGTCCCTGGGAAGTTGTGAGGAATGATGTTTCATATCCTATCAAATTCTATGGAAAAGTCTCTACAGGATCAGATGGAAAGAGGTA :  800
Pho1a-HA : GGCTTGAAATTGGCAGTCCCTGGGAAGTTGTGAGGAATGATGTTTCATATCCTATCAAATTCTATGGAAAAGTCTCTACAGGATCAGATGGAAAGAGGTA :  800
Pho1a(6) : GGCTTGAAATTGGCAGTCCCTGGGAAGTTGTGAGGAATGATGTTTCATATCCTATCAAATTCTATGGAAAAGTCTCTACAGGATCAGATGGAAAGAGGTA :  800
Pho1a(7) : GGCTTGAAATTGGCAGTCCATGGGAAGTTGTGAGGAATGATGTTTCATATCCTATCAAATTCTATGGAAAAGTCTCTACAGGATCAGATGGAAAGAGGTA :  800
Pho1a(8) : GGCTTGAAATTGGCAGTCCATGGGAAGTTGTGAGGAATGATGTTTCATATCCTATCAAATTCTATGGAAAAGTCTCTACAGGATCAGATGGAAAGAGGTA :  800
Pho1a(9) : GGCTTGAAATTGGCAGTCCATGGGAAGTTGTGAGGAATGATGTTTCATATCCTATCAAATTCTATGGAAAAGTCTCTACAGGATCAGATGGAAAGAGGTA :  800
```

```
                  *        820          *        840          *        860          *        880          *        900
Pho1a-HR : TTGGATTGGTGGAGAGGATATAAAGGCAGTTGCGTATGATGTTCCCATACCAGGGTATAAGACCAGAACCACAATCAGCCTTCGACTGTGGTCTACACAG :  900
Pho1a(2) : TTGGATTGGTGGAGAGGATATAAAGGCAGTTGCGTATGATGTTCCCATACCAGGGTATAAGACCAGAACCACAATCAGCCTTCGACTGTGGTCTACACAG :  900
Pho1a(3) : TTGGATTGGTGGAGAGGATATAAAGGCAGTTGCGTATGATGTTCCCATACCAGGGTATAAGACCAGAACCACAATCAGCCTTCGACTGTGGTCTACACAG :  900
Pho1a(4) : TTGGATTGGTGGAGAGGATATAAGGGCAGTTGCGTATGATGTTCCCATACCAGGGTATAAGACCAGAACCACAATCAGCCTTCGACTGTGGTCTACACAG :  900
Pho1a-HA : TTGGATTGGTGGAGAGGATATAAGGGCAGTTGCGTATGATGTTCCCATACCAGGGTATAAGACCAGAACCACAATCAGCCTTCGACTGTGGTCTACACAG :  900
Pho1a(6) : TTGGATTGGTGGAGAGGATATAAGGGCAGTTGCGTATGATGTTCCCATACCAGGGTATAAGACCAGAACCACAATCAGCCTTCGACTGTGGTCTACACAG :  900
Pho1a(7) : TTGGATTGGTGGAGAGGATATAAAGGCAGTTGCGTATGATGTTCCCATACCAGGGTATAAGACCAGAACCACAATCAGCCTTCGACTGTGGTCTACACAG :  900
Pho1a(8) : TTGGATTGGTGGAGAGGATATAAAGGCAGTTGCGTATGATGTTCCCATACCAGGGTATAAGACCAGAACCACAATCAGCCTTCGACTGTGGTCTACACAG :  900
Pho1a(9) : TTGGATTGGTGGAGAGGATATAAAGGCAGTTGCGTATGATGTTCCCATACCAGGGTATAAGACCAGAACCACAATCAGCCTTCGACTGTGGTCTACACAG :  900

                  *        920          *        940          *        960          *        980          *       1000
Pho1a-HR : GTTCCATCAGCGGATTTTGATTTATCTGCTTTCAATGCTGGAGAGCACACCAAAGCATGTGAAGCCCAAGCAAACGCTGAGAAGATATGTTACATACTCT : 1000
Pho1a(2) : GTTCCATCAGCGGATTTTGATTTATCTGCTTTCAATGCTGGAGAGCACACCAAAGCATGTGAAGCCCAAGCAAACGCTGAGAAGATATGTTACATACTCT : 1000
Pho1a(3) : GTTCCATCAGCGGATTTTGATTTATCTGCTTTCAATGCTGGAGAGCACACCAAAGCATGTGAAGCCCAAGCAAACGCTGAGAAGATATGTTACATACTCT : 1000
Pho1a(4) : GTTCCATCAGCGGATTTTGATTTATCTGCTTTCAATGCTGGAGAGCACACCAAAGCATGTGAAGCCCAAGCAAACGCTGAGAAGATATGTTACATACTCT : 1000
Pho1a-HA : GTTCCATCAGCGGATTTTGATTTATCTGCTTTCAATGCTGGAGAGCACACCAAAGCATGTGAAGCCCAAGCAAACGCTGAGAAGATATGTTACATACTCT : 1000
Pho1a(6) : GTTCCATCAGCGGATTTTGATTTATCTGCTTTCAATGCTGGAGAGCACACCAAAGCATGTGAAGCCCAAGCAAACGCTGAGAAGATATGTTACATACTCT : 1000
Pho1a(7) : GTTCCATCAGCGGATTTTGATTTATCTGCTTTCAATGCTGGAGAGCACACCAAAGCATGTGAAGCCCAAGCAAACGCTGAGAAGATATGTTACATACTCT : 1000
Pho1a(8) : GTTCCATCAGCGGATTTTGATTTATCTGCTTTCAATGCTGGAGAGCACACCAAAGCATGTGAAGCCCAAGCAAACGCTGAGAAGATATGTTACATACTCT : 1000
Pho1a(9) : GTTCCATCAGCGGATTTTGATTTATCTGCTTTCAATGCTGGAGAGCACACCAAAGCATGTGAAGCCCAAGCAAACGCTGAGAAGATATGTTACATACTCT : 1000

                  *       1020          *       1040          *       1060          *       1080          *       1100
Pho1a-HR : ACCCTGGGGATGAATCAGAGGAGGGAAAGATCCTTCGGTTGAAGCAACAATATACCTTATGCTCGGCTTCTCTCCAAGATATTATTTCTCGATTTGAGAG : 1100
Pho1a(2) : ACCCTGGGGATGAATCAGAGGAGGGAAAGATCCTTCGGTTGAAGCAACAATATACCTTATGCTCGGCTTCTCTCCAAGATATTATTTCTCGATTTGAGAG : 1100
Pho1a(3) : ACCCTGGGGATGAATCAGAGGAGGGAAAGATCCTTCGGTTGAAGCAACAATATACCTTATGCTCGGCTTCTCTCCAAGATATTATTTCTCGATTTGAGAG : 1100
Pho1a(4) : ACCCTGGGGATGAATCAGAGGAGGGAAAGATCCTTCGGTTGAAGCAACAATATACCTTATGCTCGGCTTCTCTCCAAGATATTATTTCTCGATTTGAGAG : 1100
Pho1a-HA : ACCCTGGGGATGAATCAGAGGAGGGAAAGATCCTTCGGTTGAAGCAACAATATACCTTATGCTCGGCTTCTCTCCAAGATATTATTTCTCGATTTGAGAG : 1100
Pho1a(6) : ACCCTGGGGATGAATCAGAGGAGGGAAAGATCCTTCGGTTGAAGCAACAATATACCTTATGCTCGGCTTCTCTCCAAGATATTATTTCTCGATTTGAGAG : 1100
Pho1a(7) : ACCCTGGGGATGAATCAGAGGAGGGAAAGATCCTTCGGTTGAAGCAACAATATACCTTATGCTCGGCTTCTCTCCAAGATATTATTTCTCGATTTGAGAG : 1100
Pho1a(8) : ACCCTGGGGATGAATCAGAGGAGGGAAAGATCCTTCGGTTGAAGCAACAATATACCTTATGCTCGGCTTCTCTCCAAGATATTATTTCTCGATTTGAGAG : 1100
Pho1a(9) : ACCCTGGGGATGAATCAGAGGAGGGAAAGATCCTTCGGTTGAAGCAACAATATACCTTATGCTCGGCTTCTCTCCAAGATATTATTTCTCGATTTGAGAG : 1100

                  *       1120          *       1140          *       1160          *       1180          *       1200
Pho1a-HR : GAGATCAGGTGATCGTATTAAGTGGGAAGAGTTTCCTGAAAAAGTTGCTGTGCAGATGAATGACACTCACCCTACACTTTGTATCCCTGAGCTGATGAGA : 1200
Pho1a(2) : GAGATCAGGTGATCGTATTAAGTGGGAAGAGTTTCCTGAAAAAGTTGCTGTGCAGATGAATGACACTCACCCTACACTTTGTATCCCTGAGCTGATGAGA : 1200
Pho1a(3) : GAGATCAGGTGATCGTATTAAGTGGGAAGAGTTTCCTGAAAAAGTTGCTGTGCAGATGAATGACACTCACCCTACACTTTGTATCCCTGAGCTGATGAGA : 1200
Pho1a(4) : GAGATCAGGTGATCGTATTAAGTGGGAAGAGTTTCCTGAAAAAGTTGCTGTGCAGATGAATGACACTCACCCTACACTTTGTATCCCTGAGCTGATGAGA : 1200
Pho1a-HA : GAGATCAGGTGATCGTATTAAGTGGGAAGAGTTTCCTGAAAAAGTTGCTGTGCAGATGAATGACACTCACCCTACACTTTGTATCCCTGAGCTGATGAGA : 1200
Pho1a(6) : GAGATCAGGTGATCGTATTAAGTGGGAAGAGTTTCCTGAAAAAGTTGCTGTGCAGATGAATGACACTCACCCTACACTTTGTATCCCTGAGCTGATGAGA : 1200
Pho1a(7) : GAGATCAGGTGATCGTATTAAGTGGGAAGAGTTTCCTGAAAAAGTTGCTGTGCAGATGAATGACACTCACCCTACACTTTGTATCCCTGAGCTGATGAGA : 1200
Pho1a(8) : GAGATCAGGTGATCGTATTAAGTGGGAAGAGTTTCCTGAAAAAGTTGCTGTGCAGATGAATGACACTCACCCTACACTTTGTATCCCTGAGCTGATGAGA : 1200
Pho1a(9) : GAGATCAGGTGATCGTATTAAGTGGGAAGAGTTTCCTGAAAAAGTTGCTGTGCAGATGAATGACACTCACCCTACACTTTGTATCCCTGAGCTGATGAGA : 1200

                  *       1220          *       1240          *       1260          *       1280          *       1300
Pho1a-HR : ATATTGATAGATCTGAAGGGCTTGAATTGGAATGAAGCTTGGAATATTACTCAAAGAACTGTGGCCTACACAAACCATACTGTTTTGCCTGAGGCACTGG : 1300
Pho1a(2) : ATATTGATAGATCTGAAGGGCTTGAATTGGAATGAAGCTTGGAATATTACTCAAAGAACTGTGGCCTACACAAACCATACTGTTTTGCCTGAGGCACTGG : 1300
Pho1a(3) : ATATTGATAGATCTGAAGGGCTTGAATTGGAATGAAGCTTGGAATATTACTCAAAGAACTGTGGCCTACACAAACCATACTGTTTTGCCTGAGGCACTGG : 1300
Pho1a(4) : ATATTGATAGATCTGAAGGGCTTGAATTGGAATGAAGCTTGGAATATTACTCAAAGAACTGTGGCCTACACAAACCATACTGTTTTGCCTGAGGCACTGG : 1300
Pho1a-HA : ATATTGATAGATCTGAAGGGCTTGAATTGGAATGAAGCTTGGAATATTACTCAAAGAACTGTGGCCTACACAAACCATACTGTTTTGCCTGAGGCACTGG : 1300
Pho1a(6) : ATATTGATAGATCTGAAGGGCTTGAATTGGAATGAAGCTTGGAATATTACTCAAAGAACTGTGGCCTACACAAACCATACTGTTTTGCCTGAGGCACTGG : 1300
Pho1a(7) : ATATTGATAGATCTGAAGGGCTTGAATTGGAATGAAGCTTGGAATATTACTCAAAGAACTGTGGCCTACACAAACCATACTGTTTTGCCTGAGGCACTGG : 1300
Pho1a(8) : ATATTGATAGATCTGAAGGGCTTGAATTGGAATGAAGCTTGGAATATTACTCAAAGAACTGTGGCCTACACAAACCATACTGTTTTGCCTGAGGCACTGG : 1300
Pho1a(9) : ATATTGATAGATCTGAAGGGCTTGAATTGGAATGAAGCTTGGAATATTACTCAAAGAACTGTGGCCTACACAAACCATACTGTTTTGCCTGAGGCACTGG : 1300

                  *       1320          *       1340          *       1360          *       1380          *       1400
Pho1a-HR : AGAAATGGAGTTATGAATTGATGCAGAAACTCCTTCCCAGACATGTCGAAATCATTGAGGCGATTGACGAGGAGCTGGTACATGAAATTGTATTAAAATA : 1400
Pho1a(2) : AGAAATGGAGTTATGAATTGATGCAGAAACTCCTTCCCAGACATGTCGAAATCATTGAGGCGATTGACGAGGAGCTGGTACATGAAATTGTATTAAAATA : 1400
Pho1a(3) : AGAAATGGAGTTATGAATTGATGCAGAAACTCCTTCCCAGACATGTCGAAATCATTGAGGCGATTGACGAGGAGCTGGTACATGAAATTGTATTAAAATA : 1400
Pho1a(4) : AGAAATGGAGTTATGAATTGATGCAGAAACTCCTTCCCAGACATGTCGAAATCATTGAGGCGATTGACGAGGAGCTGGTACATGAAATTGTATTAAAATA : 1400
Pho1a-HA : AGAAATGGAGTTATGAATTGATGCAGAAACTCCTTCCCAGACATGTCGAAATCATTGAGGCGATTGACGAGGAGCTGGTACATGAAATTGTATTAAAATA : 1400
Pho1a(6) : AGAAATGGAGTTATGAATTGATGCAGAAACTCCTTCCCAGACATGTCGAAATCATTGAGGCGATTGACGAGGAGCTGGTACATGAAATTGTATTAAAATA : 1400
Pho1a(7) : AGAAATGGAGTTATGAATTGATGCAGAAACTCCTTCCCAGACATGTCGAAATCATTGAGGCGATTGACGAGGAGCTGGTACATGAAATTGTATTAAAATA : 1400
Pho1a(8) : AGAAATGGAGTTATGAATTGATGCAGAAACTCCTTCCCAGACATGTCGAAATCATTGAGGCGATTGACGAGGAGCTGGTACATGAAATTGTATTAAAATA : 1400
Pho1a(9) : AGAAATGGAGTTATGAATTGATGCAGAAACTCCTTCCCAGACATGTCGAAATCATTGAGGCGATTGACGAGGAGCTGGTACATGAAATTGTATTAAAATA : 1400

                  *       1420          *       1440          *       1460          *       1480          *       1500
Pho1a-HR : TGGTTCAATGGATCTGAACAAATTGGAGGAAAAGTTGACTACAATGAGAATCTTAGAAAATTTTGATCTTCCCAGTTCTGTTGCTGAATTATTTATTAAG : 1500
Pho1a(2) : TGGTTCAATGGATCTGAACAAATTGGAGGAAAAGTTGACTACAATGAGAATCTTAGAAAATTTTGATCTTCCCAGTTCTGTTGCTGAATTATTTATTAAG : 1500
Pho1a(3) : TGGTTCAATGGATCTGAACAAATTGGAGGAAAAGTTGACTACAATGAGAATCTTAGAAAATTTTGATCTTCCCAGTTCTGTTGCTGAATTATTTATTAAG : 1500
Pho1a(4) : TGGTTCAATGGATCTGAACAAATTGGAGGAAAAGTTGACTACAATGAGAATCTTAGAAAATTTTGATCTTCCCAGTTCTGTTGCTGAATTATTTATTAAG : 1500
Pho1a-HA : TGGTTCAATGGATCTGAACAAATTGGAGGAAAAGTTGACTACAATGAGAATCTTAGAAAATTTTGATCTTCCCAGTTCTGTTGCTGAATTATTTATTAAG : 1500
Pho1a(6) : TGGTTCAATGGATCTGAACAAATTGGAGGAAAAGTTGACTACAATGAGAATCTTAGAAAATTTTGATCTTCCCAGTTCTGTTGCTGAATTATTTATTAAG : 1500
Pho1a(7) : TGGTTCAATGGATCTGAACAAATTGGAGGAAAAGTTGACTACAATGAGAATCTTAGAAAATTTTGATCTTCCCAGTTCTGTTGCTGAATTATTTATTAAG : 1500
Pho1a(8) : TGGTTCAATGGATCTGAACAAATTGGAGGAAAAGTTGACTACAATGAGAATCTTAGAAAATTTTGATCTTCCCAGTTCTGTTGCTGAATTATTTATTAAG : 1500
Pho1a(9) : TGGTTCAATGGATCTGAACAAATTGGAGGAAAAGTTGACTACAATGAGAATCTTAGAAAATTTTGATCTTCCCAGTTCTGTTGCTGAATTATTTATTAAG : 1500

                  *       1520          *       1540          *       1560          *       1580          *       1600
Pho1a-HR : CCTGAAATCTCAGTTGATGATGATACTGAAACAGTAGAAGTCCATGACAAAGTTGAAGCTTCCGATAAAGTTGTGACTAATGATGAAGATGACACTGGTA : 1600
Pho1a(2) : CCTGAAATCTCAGTTGATGATGATACTGAAACAGTAGAAGTCCATGACAAAGTTGAAGCTTCCGATAAAGTTGTGACTAATGATGAAGATGACACTGGTA : 1600
Pho1a(3) : CCTGAAATCTCAGTTGATGATGATACTGAAACAGTAGAAGTCCATGACAAAGTTGAAGCTTCCGATAAAGTTGTGACTAATGATGAAGATGACACTGGTA : 1600
Pho1a(4) : CCTGAAATCTCAGTTGATGATGATACTGAAACAGTAGAAGTCCATGACAAAGTTGAAGCTTCCGATAAAGTTGTGACTAATGATGAAGATGACACTGGTA : 1600
Pho1a-HA : CCTGAAATCTCAGTTGATGATGATACTGAAACAGTAGAAGTCCATGACAAAGTTGAAGCTTCCGATAAAGTTGTGACTAATGATGAAGATGACACTGGTA : 1600
Pho1a(6) : CCTGAAATCTCAGTTGATGATGATACTGAAACAGTAGAAGTCCATGACAAAGTTGAAGCTTCCGATAAAGTTGTGACTAATGATGAAGATGACACTGGTA : 1600
Pho1a(7) : CCTGAAATCTCAGTTGATGATGATACTGAAACAGTAGAAGTCCATGACAAAGTTGAAGCTTCCGATAAAGTTGTGACTAATGATGAAGATGACACTGGTA : 1600
Pho1a(8) : CCTGAAATCTCAGTTGATGATGATACTGAAACAGTAGAAGTCCATGACAAAGTTGAAGCTTCCGATAAAGTTGTGACTAATGATGAAGATGACACTGGTA : 1600
Pho1a(9) : CCTGAAATCTCAGTTGATGATGATACTGAAACAGTAGAAGTCCATGACAAAGTTGAAGCTTCCGATAAAGTTGTGACTAATGATGAAGATGACACTGGTA : 1600

                  *       1620          *       1640          *       1660          *       1680          *       1700
Pho1a-HR : AGAAAACTAGTGTGAAGATAGAAGCAGCTGCAGAAAAAGACATTGACAAGAAAACTCCCGTGAGTCCGGAACCAGCTGTTATACCACCTAAGAAGGTACG : 1700
Pho1a(2) : AGAAAACTAGTGTGAAGATAGAAGCAGCTGCAGAAAAAGACATTGACAAGAAAACTCCCGTGAGTCCGGAACCAGCTGTTATACCACCTAAGAAGGTACG : 1700
Pho1a(3) : AGAAAACTAGTGTGAAGATAGAAGCAGCTGCAGAAAAAGACATTGACAAGAAAACTCCCGTGAGTCCGGAACCAGCTGTTATACCACCTAAGAAGGTACG : 1700
Pho1a(4) : AGAAAACTAGTGTGAAGATAGAAGCAGCTGCAGAAAAAGACATTGACAAGAAAACTCCCGTGAGTCCGGAACCAGCTGTTATACCACCTAAGAAGGTACG : 1700
Pho1a-HA : AGAAAACTAGTGTGAAGATAGAAGCAGCTGCAGAAAAAGACATTGACAAGAAAACTCCCGTGAGTCCGGAACCAGCTGTTATACCACCTAAGAAGGTACG : 1700
Pho1a(6) : AGAAAACTAGTGTGAAGATAGAAGCAGCTGCAGAAAAAGACATTGACAAGAAAACTCCCGTGAGTCCGGAACCAGCTGTTATACCACCTAAGAAGGTACG : 1700
Pho1a(7) : AGAAAACTAGTGTGAAGATAGAAGCAGCTGCAGAAAAAGACATTGACAAGAAAACTCCCGTGAGTCCGGAACCAGCTGTTATACCACCTAAGAAGGTACG : 1700
Pho1a(8) : AGAAAACTAGTGTGAAGATAGAAGCAGCTGCAGAAAAAGACATTGACAAGAAAACTCCCGTGAGTCCGGAACCAGCTGTTATACCACCTAAGAAGGTACG : 1700
Pho1a(9) : AGAAAACTAGTGTGAAGATAGAAGCAGCTGCAGAAAAAGACATTGACAAGAAAACTCCCGTGAGTCCGGAACCAGCTGTTATACCACCTAAGAAGGTACG : 1700
```

```
                    *     1720          *     1740          *     1760          *     1780          *     1800
Pho1a-HR : CATGGCCAACTTGTGTGTTGTGGGCGGCCATGCTGTTAATGGAGTTGCTGAGATCCATAGTGAAATTGTGAAGGAGGAGGTTTTCAATGACTTCTATGAG : 1800
Pho1a(2) : CATGGCCAACTTGTGTGTTGTGGGCGGCCATGCTGTTAATGGAGTTGCTGAGATCCATAGTGAAATTGTGAAGGAGGAGGTTTTCAATGACTTCTATGAG : 1800
Pho1a(3) : CATGGCCAACTTGTGTGTTGTGGGCGGCCATGCTGTTAATGGAGTTGCTGAGATCCATAGTGAAATTGTGAAGGAGGAGGTTTTCAATGACTTCTATGAG : 1800
Pho1a(4) : CATGGCCAACTTGTGTGTTGTGGGCGGCCATGCTGTTAATGGAGTTGCTGAGATCCATAGTGAAATTGTGAAGGAGGAGGTTTTCAATGACTTCTATGAG : 1800
Pho1a-HA : CATGGCCAACTTGTGTGTTGTGGGCGGCCATGCTGTTAATGGAGTTGCTGAGATCCATAGTGAAATTGTGAAGGAGGAGGTTTTCAATGACTTCTATGAG : 1800
Pho1a(6) : CATGGCCAACTTGTGTGTTGTGGGCGGCCATGCTGTTAATGGAGTTGCTGAGATCCATAGTGAAATTGTGAAGGAGGAGGTTTTCAATGACTTCTATGAG : 1800
Pho1a(7) : CATGGCCAACTTGTGTGTTGTGGGCGGCCATGCTGTTAATGGAGTTGCTGAGATCCATAGTGAAATTGTGAAGGAGGAGGTTTTCAATGACTTCTATGAG : 1800
Pho1a(8) : CATGGCCAACTTGTGTGTTGTGGGCGGCCATGCTGTTAATGGAGTTGCTGAGATCCATAGTGAAATTGTGAAGGAGGAGGTTTTCAATGACTTCTATGAG : 1800
Pho1a(9) : CATGGCCAACTTGTGTGTTGTGGGCGGCCATGCTGTTAATGGAGTTGCTGAGATCCATAGTGAAATTGTGAAGGAGGAGGTTTTCAATGACTTCTATGAG : 1800

                    *     1820          *     1840          *     1860          *     1880          *     1900
Pho1a-HR : CTCTGGCCGGAAAAGTTCCAAAACAAAACAAATGGAGTGACTCCAAGAAGATGGATTCGTTTCTGCAATCCTCCTCTTAGTGCCATCATAACTAAGTGGA : 1900
Pho1a(2) : CTCTGGCCGGAAAAGTTCCAAAACAAAACAAATGGAGTGACTCCAAGAAGATGGATTCGTTTCTGCAATCCTCCTCTTAGTGCCATCATAACTAAGTGGA : 1900
Pho1a(3) : CTCTGGCCGGAAAAGTTCCAAAACAAAACAAATGGAGTGACTCCAAGAAGATGGATTCGTTTCTGCAATCCTCCTCTTAGTGCCATCATAACTAAGTGGA : 1900
Pho1a(4) : CTCTGGCCGGAAAAGTTCCAAAACAAAACAAATGGAGTGACTCCAAGAAGATGGATTCGTTTCTGCAATCCTCCTCTTAGTGCCATCATAACTAAGTGGA : 1900
Pho1a-HA : CTCTGGCCGGAAAAGTTCCAAAACAAAACAAATGGAGTGACTCCAAGAAGATGGATTCGTTTCTGCAATCCTCCTCTTAGTGCCATCATAACTAAGTGGA : 1900
Pho1a(6) : CTCTGGCCGGAAAAGTTCCAAAACAAAACAAATGGAGTGACTCCAAGAAGATGGATTCGTTTCTGCAATCCTCCTCTTAGTGCCATCATAACTAAGTGGA : 1900
Pho1a(7) : CTCTGGCCGGAAAAGTTCCAAAACAAAACAAATGGAGTGACTCCAAGAAGATGGATTCGTTTCTGCAATCCTCCTCTTAGTGCCATCATAACTAAGTGGA : 1900
Pho1a(8) : CTCTGGCCGGAAAAGTTCCAAAACAAAACAAATGGAGTGACTCCAAGAAGATGGATTCGTTTCTGCAATCCTCCTCTTAGTGCCATCATAACTAAGTGGA : 1900
Pho1a(9) : CTCTGGCCGGAAAAGTTCCAAAACAAAACAAATGGAGTGACTCCAAGAAGATGGATTCGTTTCTGCAATCCTCCTCTTAGTGCCATCATAACTAAGTGGA : 1900

                    *     1920          *     1940          *     1960          *     1980          *     2000
Pho1a-HR : CTGGTACAGAGGATTGGGTCCTGAAAACTGAAAAGTTGGCAGAATTGCAGAAGTTTGCTGATAATGAAGATCTTCAAAATGAGTGGAGGGAAGCAAAAAG : 2000
Pho1a(2) : CTGGTACAGAGGATTGGGTCCTGAAAACTGAAAAGTTGGCAGAATTGCAGAAGTTTGCTGATAATGAAGATCTTCAAAATGAGTGGAGGGAAGCAAAAAG : 2000
Pho1a(3) : CTGGTACAGAGGATTGGGTCCTGAAAACTGAAAAGTTGGCAGAATTGCAGAAGTTTGCTGATAATGAAGATCTTCAAAATGAGTGGAGGGAAGCAAAAAG : 2000
Pho1a(4) : CTGGTACAGAGGATTGGGTCCTGAAAACTGAAAAGTTGGCAGAATTGCAGAAGTTTGCTGATAATGAAGATCTTCAAAATGAGTGGAGGGAAGCAAAAAG : 2000
Pho1a-HA : CTGGTACAGAGGATTGGGTCCTTAAAACTGAAAAGTTGTCAGAATTGCAGAAGTTTGCTGATAATGAAGATCTTCAAAATGAGTGGAGGGAAGCAAAAAG : 2000
Pho1a(6) : CTGGTACAGAGGATTGGGTCCTTAAAACTGAAAAGTTGTCAGAATTGCAGAAGTTTGCTGATAATGAAGATCTTCAAAATGAGTGGAGGGAAGCAAAAAG : 2000
Pho1a(7) : CTGGTACAGAGGATTGGGTCCTGAAAACTGAAAAGTTGGCAGAATTGCAGAAGTTTGCTGATAATGAAGATCTTCAAAATGAGTGGAGGGAAGCAAAAAG : 2000
Pho1a(8) : CTGGTACAGAGGATTGGGTCCTGAAAACTGAAAAGTTGGCAGAATTGCAGAAGTTTGCTGATAATGAAGATCTTCAAAATGAGTGGAGGGAAGCAAAAAG : 2000
Pho1a(9) : CTGGTACAGAGGATTGGGTCCTGAAAACTGAAAAGTTGGCAGAATTGCAGAAGTTTGCTGATAATGAAGATCTTCAAAATGAGTGGAGGGAAGCAAAAAG : 2000

                    *     2020          *     2040          *     2060          *     2080          *     2100
Pho1a-HR : GAGCAACAAGATTAAAGTTGTCTCCTTTCTCAAAGAAAAGACAGGGTATTCTGTTGTCCCAGATGCAATGTTTGATATTCAGGTAAAACGCATTCATGAG : 2100
Pho1a(2) : GAGCAACAAGATTAAAGTTGTCTCCTTTCTCAAAGAAAAGACAGGGTATTCTGTTGTCCCAGATGCAATGTTTGATATTCAGGTAAAACGCATTCATGAG : 2100
Pho1a(3) : GAGCAACAAGATTAAAGTTGTCTCCTTTCTCAAAGAAAAGACAGGGTATTCTGTTGTCCCAGATGCAATGTTTGATATTCAGGTAAAACGCATTCATGAG : 2100
Pho1a(4) : GAGCAACAAGATTAAAGTTGTCTCCTTTCTCAAAGAAAAGACAGGGTATTCTGTTGTCCCAGATGCAATGTTTGATATTCAGGTAAAACGCATTCATGAG : 2100
Pho1a-HA : GAGCAACAAGATTAAAGTTGTCTCCTTTCTCAAAGAAAAGACAGGGTATTCTGTTGTCCCAGATGCAATGTTTGATATTCAGGTAAAACGCATTCATGAG : 2100
Pho1a(6) : GAGCAACAAGATTAAAGTTGTCTCCTTTCTCAAAGAAAAGACAGGGTATTCTGTTGTCCCAGATGCAATGTTTGATATTCAGGTAAAACGCATTCATGAG : 2100
Pho1a(7) : GAGCAACAAGATTAAAGTTGTCTCCTTTCTCAAAGAAAAGACAGGGTATTCTGTTGTCCCAGATGCAATGTTTGATATTCAGGTAAAACGCATTCATGAG : 2100
Pho1a(8) : GAGCAACAAGATTAAAGTTGTCTCCTTTCTCAAAGAAAAGACAGGGTATTCTGTTGTCCCAGATGCAATGTTTGATATTCAGGTAAAACGCATTCATGAG : 2100
Pho1a(9) : GAGCAACAAGATTAAAGTTGTCTCCTTTCTCAAAGAAAAGACAGGGTATTCTGTTGTCCCAGATGCAATGTTTGATATTCAGGTAAAACGCATTCATGAG : 2100

                    *     2120          *     2140          *     2160          *     2180          *     2200
Pho1a-HR : TACAAGCGACAACTGTTAAATATCTTCGGCATCGTTTATCGGTATAAGAAGATGAAAGAAATGACAGCTGCAGAAAGAAAGACTAACTTCGTTCCTCGAG : 2200
Pho1a(2) : TACAAGCGACAACTGTTAAATATCTTCGGCATCGTTTATCGGTATAAGAAGATGAAAGAAATGACAGCTGCAGAAAGAAAGACTAACTTCGTTCCTCGAG : 2200
Pho1a(3) : TACAAGCGACAACTGTTAAATATCTTCGGCATCGTTTATCGGTATAAGAAGATGAAAGAAATGACAGCTGCAGAAAGAAAGACTAACTTCGTTCCTCGAG : 2200
Pho1a(4) : TACAAGCGACAACTGTTAAATATCTTCGGCATCGTTTATCGGTATAAGAAGATGAAAGAAATGACAGCTGCAGAAAGAAAGACTAACTTCGTTCCTCGAG : 2200
Pho1a-HA : TACAAGCGACAACTGTTAAATATCTTCGGCATCGTTTATCGGTATAAGAAGATGAAAGAAATGACAGCTGCAGAAAGAAAGACTAACTTCGTTCCTCGAG : 2200
Pho1a(6) : TACAAGCGACAACTGTTAAATATCTTCGGCATCGTTTATCGGTATAAGAAGATGAAAGAAATGACAGCTGCAGAAAGAAAGACTAACTTCGTTCCTCGAG : 2200
Pho1a(7) : TACAAGCGACAACTGTTAAATATCTTCGGCATCGTTTATCGGTATAAGAAGATGAAAGAAATGACAGCTGCAGAAAGAAAGACTAACTTCGTTCCTCGAG : 2200
Pho1a(8) : TACAAGCGACAACTGTTAAATATCTTCGGCATCGTTTATCGGTATAAGAAGATGAAAGAAATGACAGCTGCAGAAAGAAAGACTAACTTCGTTCCTCGAG : 2200
Pho1a(9) : TACAAGCGACAACTGTTAAATATCTTCGGCATCGTTTATCGGTATAAGAAGATGAAAGAAATGACAGCTGCAGAAAGAAAGACTAACTTCGTTCCTCGAG : 2200

                    *     2220          *     2240          *     2260          *     2280          *     2300
Pho1a-HR : TATGCATATTTGGGGGAAAAGCTTTTGCCACATATGTGCAAGCCAAGAGGATTGTAAAATTTATCACAGATGTTGGTGCTACTATAAATCATGATCCAGA : 2300
Pho1a(2) : TATGCATATTTGGGGGAAAAGCTTTTGCCACATATGTGCAAGCCAAGAGGATTGTAAAATTTATCACAGATGTTGGTGCTACTATAAATCATGATCCAGA : 2300
Pho1a(3) : TATGCATATTTGGGGGAAAAGCTTTTGCCACATATGTGCAAGCCAAGAGGATTGTAAAATTTATCACAGATGTTGGTGCTACTATAAATCATGATCCAGA : 2300
Pho1a(4) : TATGCATATTTGGGGGAAAAGCTTTTGCCACATATGTGCAAGCCAAGAGGATTGTAAAATTTATCACAGATGTTGGTGCTACTATAAATCATGATCCAGA : 2300
Pho1a-HA : TATGCATATTTGGGGGAAAAGCTTTTGCCACATATGTGCAAGCCAAGAGGATTGTAAAATTTATCACAGATGTTGGTGCTACTATAAATCATGATCCAGA : 2300
Pho1a(6) : TATGCATATTTGGGGGAAAAGCTTTTGCCACATATGTGCAAGCCAAGAGGATTGTAAAATTTATCACAGATGTTGGTGCTACTATAAATCATGATCCAGA : 2300
Pho1a(7) : TATGCATATTTGGGGGAAAAGCTTTTGCCACATATGTGCAAGCCAAGAGGATTGTAAAATTTATCACAGATGTTGGTGCTACTATAAATCATGATCCAGA : 2300
Pho1a(8) : TATGCATATTTGGGGGAAAAGCTTTTGCCACATATGTGCAAGCCAAGAGGATTGTAAAATTTATCACAGATGTTGGTGCTACTATAAATCATGATCCAGA : 2300
Pho1a(9) : TATGCATATTTGGGGGAAAAGCTTTTGCCACATATGTGCAAGCCAAGAGGATTGTAAAATTTATCACAGATGTTGGTGCTACTATAAATCATGATCCAGA : 2300

                    *     2320          *     2340          *     2360          *     2380          *     2400
Pho1a-HR : AATCGGTGATCTGTTGAAGGTAGTCTTTGTGCCAGATTACAATGTCAGTGTTGCTGAATTGCTAATTCCTGCTAGCGATCTATCAGAACATATCAGTACG : 2400
Pho1a(2) : AATCGGTGATCTGTTGAAGGTAGTCTTTGTGCCAGATTACAATGTCAGTGTTGCTGAATTGCTAATTCCTGCTAGCGATCTATCAGAACATATCAGTACG : 2400
Pho1a(3) : AATCGGTGATCTGTTGAAGGTAGTCTTTGTGCCAGATTACAATGTCAGTGTTGCTGAATTGCTAATTCCTGCTAGCGATCTATCAGAACATATCAGTACG : 2400
Pho1a(4) : AATCGGTGATCTGTTGAAGGTAGTCTTTGTGCCAGATTACAATGTCAGTGTTGCTGAATTGCTAATTCCTGCTAGCGATCTATCAGAACATATCAGTACG : 2400
Pho1a-HA : AATCGGTGATCTGTTGAAGGTAGTCTTTGTGCCAGATTACAATGTCAGTGTTGCTGAATTGCTAATTCCTGCTAGCGATCTATCAGAACATATCAGTACG : 2400
Pho1a(6) : AATCGGTGATCTGTTGAAGGTAGTCTTTGTGCCAGATTACAATGTCAGTGTTGCTGAATTGCTAATTCCTGCTAGCGATCTATCAGAACATATCAGTACG : 2400
Pho1a(7) : AATCGGTGATCTGTTGAAGGTAGTCTTTGTGCCAGATTACAATGTCAGTGTTGCTGAATTGCTAATTCCTGCTAGCGATCTATCAGAACATATCAGTACG : 2400
Pho1a(8) : AATCGGTGATCTGTTGAAGGTAGTCTTTGTGCCAGATTACAATGTCAGTGTTGCTGAATTGCTAATTCCTGCTAGCGATCTATCAGAACATATCAGTACG : 2400
Pho1a(9) : AATCGGTGATCTGTTGAAGGTAGTCTTTGTGCCAGATTACAATGTCAGTGTTGCTGAATTGCTAATTCCTGCTAGCGATCTATCAGAACATATCAGTACG : 2400

                    *     2420          *     2440          *     2460          *     2480          *     2500
Pho1a-HR : GCTGGAATGGAGGCCAGTGGAACCAGTAATATGAAGTTTGCAATGAATGGTTGTATCCAAATTGGTACATTGGATGGCGCTAATGTTGAAATAAGGGAAG : 2500
Pho1a(2) : GCTGGAATGGAGGCCAGTGGAACCAGTAATATGAAGTTTGCAATGAATGGTTGTATCCAAATTGGTACATTGGATGGCGCTAATGTTGAAATAAGGGAAG : 2500
Pho1a(3) : GCTGGAATGGAGGCCAGTGGAACCAGTAATATGAAGTTTGCAATGAATGGTTGTATCCAAATTGGTACATTGGATGGCGCTAATGTTGAAATAAGGGAAG : 2500
Pho1a(4) : GCTGGAATGGAGGCCAGTGGAACCAGTAATATGAAGTTTGCAATGAATGGTTGTATCCAAATTGGTACATTGGATGGCGCTAATGTTGAAATAAGGGAAG : 2500
Pho1a-HA : GCTGGAATGGAGGCCAGTGGAACCAGTAATATGAAGTTTGCAATGAATGGTTGTATCCAAATTGGTACATTGGATGGCGCTAATGTTGAAATAAGGGAAG : 2500
Pho1a(6) : GCTGGAATGGAGGCCAGTGGAACCAGTAATATGAAGTTTGCAATGAATGGTTGTATCCAAATTGGTACATTGGATGGCGCTAATGTTGAAATAAGGGAAG : 2500
Pho1a(7) : GCTGGAATGGAGGCCAGTGGAACCAGTAATATGAAGTTTGCAATGAATGGTTGTATCCAAATTGGTACATTGGATGGCGCTAATGTTGAAATAAGGGAAG : 2500
Pho1a(8) : GCTGGAATGGAGGCCAGTGGAACCAGTAATATGAAGTTTGCAATGAATGGTTGTATCCAAATTGGTACATTGGATGGCGCTAATGTTGAAATAAGGGAAG : 2500
Pho1a(9) : GCTGGAATGGAGGCCAGTGGAACCAGTAATATGAAGTTTGCAATGAATGGTTGTATCCAAATTGGTACATTGGATGGCGCTAATGTTGAAATAAGGGAAG : 2500

                    *     2520          *     2540          *     2560          *     2580          *     2600
Pho1a-HR : AGGTTGGAGAAGAAAACTTCTTTCTCTTTGGTGCTCAAGCTCATGAAATTGCAGGGCTTAGAAAAGAAAGAGCTGACGGAAAGTTTGTACCTGATGAACG : 2600
Pho1a(2) : AGGTTGGAGAAGAAAACTTCTTTCTCTTTGGTGCTCAAGCTCATGAAATTGCAGGGCTTAGAAAAGAAAGAGCTGACGGAAAGTTTGTACCTGATGAACG : 2600
Pho1a(3) : AGGTTGGAGAAGAAAACTTCTTTCTCTTTGGTGCTCAAGCTCATGAAATTGCAGGGCTTAGAAAAGAAAGAGCTGACGGAAAGTTTGTACCTGATGAACG : 2600
Pho1a(4) : AGGTTGGAGAAGAAAACTTCTTTCTCTTTGGTGCTCAAGCTCATGAAATTGCAGGGCTTAGAAAAGAAAGAGCTGACGGAAAGTTTGTACCTGATGAACG : 2600
Pho1a-HA : AGGTTGGAGAAGAAAACTTCTTTCTCTTTGGTGCTCAAGCTCATGAAATTGCAGGGCTTAGAAAAGAAAGAGCTGACGGAAAGTTTGTACCTGATGAACG : 2600
Pho1a(6) : AGGTTGGAGAAGAAAACTTCTTTCTCTTTGGTGCTCAAGCTCATGAAATTGCAGGGCTTAGAAAAGAAAGAGCTGACGGAAAGTTTGTACCTGATGAACG : 2600
Pho1a(7) : AGGTTGGAGAAGAAAACTTCTTTCTCTTTGGTGCTCAAGCTCATGAAATTGCAGGGCTTAGAAAAGAAAGAGCTGACAGAAAGTTTGTACCTGATGAACG : 2600
Pho1a(8) : AGGTTGGAGAAGAAAACTTCTTTCTCTTTGGTGCTCAAGCTCATGAAATTGCAGGGCTTAGAAAAGAAAGAGCTGACAGAAAGTTTGTACCTGATGAACG : 2600
Pho1a(9) : AGGTTGGAGAAGAAAACTTCTTTCTCTTTGGTGCTCAAGCTCATGAAATTGCAGGGCTTAGAAAAGAAAGAGCTGACAGAAAGTTTGTACCTGATGAACG : 2600
```

```
                        *        2620          *        2640          *        2660          *        2680          *        2700
Pho1a-HR : TTTTGAAGAGGTGAAGGAATTTGTTAGAAGCGGTGCTTTTGGCTCTTATAACTATGATGACCTAATTGGATCGTTGGAAGGAAATGAAGGTTTTGGCCGT : 2700
Pho1a(2) : TTTTGAAGAGGTGAAGGAATTTGTTAGAAGCGGTGCTTTTGGCTCTTATAACTATGATGACCTAATTGGATCGTTGGAAGGAAATGAAGGTTTTGGCCGT : 2700
Pho1a(3) : TTTTGAAGAGGTGAAGGAATTTGTTAGAAGCGGTGCTTTTGGCTCTTATAACTATGATGACCTAATTGGATCGTTGGAAGGAAATGAAGGTTTTGGCCGT : 2700
Pho1a(4) : TTTTGAAGAGGTGAAGGAATTTGTTAGAAGCGGTGCTTTTGGCTCTTATAACTATGATGACCTAATTGGATCGTTGGAAGGAAATGAAGGTTTTGGCCGT : 2700
Pho1a-HA : TTTTGAAGAGGTGAAGGAATTTGTTAGAAGCGGTGCTTTTGGCTCTTATAACTATGATGACCTAATTGGATCGTTGGAAGGAAATGAAGGTTTTGGCCGT : 2700
Pho1a(6) : TTTTGAAGAGGTGAAGGAATTTGTTAGAAGCGGTGCTTTTGGCTCTTATAACTATGATGACCTAATTGGATCGTTGGAAGGAAATGAAGGTTTTGGCCGT : 2700
Pho1a(7) : TTTTGAAGAGGTGAAGGAATTTGTTAGAAGCGGTGCTTTTGGCTCTTATAACTATGATGACCTAATTGGATCGTTGGAAGGAAATGAAGGTTTTGGCCGT : 2700
Pho1a(8) : TTTTGAAGAGGTGAAGGAATTTGTTAGAAGCGGTGCTTTTGGCTCTTATAACTATGATGACCTAATTGGATCGTTGGAAGGAAATGAAGGTTTTGGCCGT : 2700
Pho1a(9) : TTTTGAAGAGGTGAAAGAATTTGTTAGAAGCGGTGCTTTTGGCTCTTATAACTATGATGACCTAATTGGATCGTTGGAAGGAAATGAAGGTTTTGGCCGT : 2700

                        *        2720          *        2740          *        2760          *        2780          *        2800
Pho1a-HR : GCTGACTATTTCCTTGTGGGCAAGGACTTCCCCAGTTACATAGAATGCCAAGAGAAAGTTGATGAGGCATATCGCGACCAGAAAAGGTGGACAACGATGT : 2800
Pho1a(2) : GCTGACTATTTCCTTGTGGGCAAGGACTTCCCCAGTTACATAGAATGCCAAGAGAAAGTTGATGAGGCATATCGCGACCAGAAAAGGTGGACAAAGATGT : 2800
Pho1a(3) : GCTGACTATTTCCTTGTGGGCAAGGACTTCCCCAGTTACATAGAATGCCAAGAGAAAGTTGATGAGGCATATCGCAACCAGAAAAGGTGGACAAAGATGT : 2800
Pho1a(4) : GCTGACTATTTCCTTGTGGGCAAGGACTTCCCCAGTTACATAGAATGCCAAGAGAAAGTTGATGAGGCATATCGCGACCAGAAAAGGTGGACAACGATGT : 2800
Pho1a-HA : GCTGACTATTTCCTTGTGGGCAAGGACTTCCCCAGTTACATAGAATGCCAAGAGAAAGTTGATGAGGCATATCGCAACCAGAAAAGGTGGACAAAGATGT : 2800
Pho1a(6) : GCTGACTATTTCCTTGTGGGCAAGGACTTCCCCAGTTACATAGAATGCCAAGAGAAAGTTGATGAGGCATATCGCGACCAGAAAAGGTGGACAAAGATGT : 2800
Pho1a(7) : GCTGACTATTTCCTTGTGGGCAAGGACTTCCCCAGTTACATAGAATGCCAAGAAAAAGTTGATGAGGCATATCGCGACCAGAAAAGGTGGACAAAGATGT : 2800
Pho1a(8) : GCTGACTATTTCCTTGTGGGCAAGGACTTCCCCAGTTACATAGAATGCCAAGAAAAAGTTGATGAGGCATATCGCGACCAGAAAAGGTGGACAAAGATGT : 2800
Pho1a(9) : GCTGACTATTTCCTTGTGGGCAAGGACTTCCCCAGTTACATAGAATGCCAAGAGAAAGTTGATGTGGCATATCGCGACCAGAAAAGGTGGACAAAGATGT : 2800

                        *        2820          *        2840          *        2860          *        2880          *        2900
Pho1a-HR : CAATCTTGAATACAGCGGGATCGTACAAGTTCAGCAGTGACAGAACAATCCATGAATATGCCAAAGACATTTGGAACATTGAAGCTGTGGAAATAGCATA : 2900
Pho1a(2) : CAATCTTGAATACAGCGGGATCCTACAAGTTCAGCAGTGACAGAACAATCCATGAATATGCCAAAGACATTTGGAACATTGAAGCTGTGGAAATAGCATA : 2900
Pho1a(3) : CAATCTTGAATACAGCGGGATCCTACAAGTTCAGCAGTGACAGAACAATCCATGAATATGCCAAAGACATTTGGAACATTGAAGCTGTGGAAATAGCATA : 2900
Pho1a(4) : CAATCTTGAATACAGCGGGATCGTACAAGTTCAGCAGTGACAGAACAATCCATGAATATGCCAAAGACATTTGGAACATTGAAGCTGTGGAAATAGCATA : 2900
Pho1a-HA : CAATCTTGAATACAGCGGGATCCTACAAGTTCAGCAGTGACAGAACAATCCATGAATATGCCAAAGACATTTGGAACATTGAAGCTGTGGAAATAGCATA : 2900
Pho1a(6) : CAATCTTGAATACAGCGGGATCCTACAAGTTCAGCAGTGACAGAACAATCCATGAATATGCCAAAGACATTTGGAACATTGAAGCTGTGGAAATAGCATA : 2900
Pho1a(7) : CAATCTTGAATACAGCGGGATCCTACAAGTTCAGCAGTGACAGAACAATCCATGAATATGCCAAAGACATTTGGAACATTGAAGCTGTGGAAATAGCATA : 2900
Pho1a(8) : CAATCTTGAATACAGCGGGATCGTACAAGTTCAGCAGTGACAGAACAATCCATGAATATGCCAAAGACATTTGGAACATTGAAGCTGTGGAAATAGCATA : 2900
Pho1a(9) : CAATCTTGAATACAGCGGGATCGTACAAGTTCAGCAGTGACAGAACAATCCATGAATATGCCAAAGACATTTGGAACATTGAAGCTGTGGAAATAGCATA : 2900


Pho1a-HR : A : 2901
Pho1a(2) : A : 2901
Pho1a(3) : A : 2901
Pho1a(4) : A : 2901
Pho1a-HA : A : 2901
Pho1a(6) : A : 2901
Pho1a(7) : A : 2901
Pho1a(8) : A : 2901
Pho1a(9) : A : 2901
```

Figure S1 Sequence alignment of nine Pho1a cDNA alleles.
