# Supplementary material for: SNPs in Genes Functional in Starch-Sugar Interconversion Associate with Natural Variation of Tuber Starch and Sugar Content of Potato (Solanum tuberosum L.)
Source: G3 (Bethesda). 2014 Jul 31;4(10):1797–811. doi: 10.1534/g3.114.012377 (PMC4199688; doi:10.1534/g3.114.012377)
Supplement: Supporting Information [file supp_g3.114.012377_FigureS2.pdf]

```
                    *        20         *        40         *        60         *        80         *       100         *       120
SOLTU-HA : --------------MATANGAYLFNHYSSNSRFIHFTSRNTSSKLFLTKTS----HFRRP--------KRCFHVNNT----LSEKIHHPITEQGGE---SDLSSFAPDAASITSSIKYHA :  87
SOLTU-HR : --------------MATANGAHLFNHYSSNSRFIHFTSRNTSSKLFLTKTS----HFRRP--------KRCFHVNNT----LSEKIHHPITEQGGE---SDLSSFAPDAASITSSIKYHA :  87
SPIOL    : MATLPLSSTTPSTGRTENCFSSYYSSSISRVMEFGLKNGCNSKLLFSSVNY----KPMIMR-----GSRRCIVIRNV-FSESKPKSEEPIIEQETPSILNPLSNLSPDSASRQSSIKYHA : 110
SOLTUb   : ----------------MATFAVSGLNSISSISSFNNNFRSKNSNILLSRRRILLFSFRRR--------RRSFSVSSV-ASDQKQKTKDSSSDEG-----FTLDVFQPDSTSVLSSIKYHA :  90
POPTRa   : --------------MATLPFSAAQSSSVS-----GFNYRASHSNLFFVRTP----RFFNR------LKRRNLSVKNI-TSDQRQELKDPSVNGE-----ASLETLEPDSASIAASIQYHA :  85
RICCOb   : --------------MANLPFPTAKSNSRFSSTFIDFNYGTAHSKLFFIRSPQ---RFSRS-------RTRTLSVKNI-ASDQRQQDLQEHITQG-----DSLASFIPDSASIASSIKYHA :  90
WHEAT    : ------------MATASPPLATAFR----PLAAAGGAGGGGAHAVGAAGRV----APRRG--------RRGFVVRSV---ASDREVRGPASTEEE----LSAVLTSIDSSAIASNIQHHA :  85
ORYSJa   : ------------MATASAPLQLATASRPLPVGVGCGGGGGGGLHVGGARGG----GAAPA--------RRRLAVRSV---ASDRGVQGSVSPEEE----ISSVLNSIDSSTIASNIKHHA :  89
ORYSJb   : ------------MATASAPLQLATASRPLPVGVGCGGGGGGGLHVGGARGG----GAAPA--------RRRLAVRSV---ASDRGVQGSVSPEEE----ISSVLNSIDSSTIASNIKHHA :  89
CUCMA    : -------MAASSHLTVALNRAEPFSGSNSLPSLIGLSTRYGKSRLLLVRTS----SWRSP--------KRTLVVRNV-SSEPTQKLKDPVADEESA---TGASTFEPDSSSIAASIKYHA :  97
IPOBA    : --------------------MSRLSGITPRARDDRSQFQNPRLEIAVPDRTA---GLQRT--------KRTLLVKCV-LDETKQTIQHVVTEKNEG--------TLLDAASIASSIKYHA :  80
VICFA    : ---------MASMTMRFHPNSTAVTESVPRRGSVYGFIGYRSSSLFVRTNVI---KYRSVKRNLEFRRRSAFSVKCGSGNEAKQKVKDQEVQQEAK---TSPSSFAPDTTSIVSSIKYHA : 105
POPTRb   : ---------------------------------------MNSRRVVKRRSV-------------------SVSIKNVSSSEPKQKLKDDALIEEEVP--RILNPSTPNASSIASSIKYHA :  60
RICCOa   : -------MASSSGTGSHLCRSWQCSGVSRFVHFGAKSSSKWRSNLLLIRTF----RSRPV--------STSFSVRNV-STEPKTKIVDSLSHEAAP---SNRSLFNLDSSSIASSIKYHA :  97
GOSHI    : --------------------------------------------------------------------GTRFSVRNV-SSEQQQKVKDLVTQQQESP--GSYNPFPPDASSIASSIKYHS :  49
ARATH    : -----MDTMRISGVSTGAEVLIQCNSLSSLVSRRC-DDGKWRTRMFPARNR----DLRPSP-----TRRSFLSVKSI-SSEPKAKVTDAVLDSEQEVFISSMNPFAPDAASVASSIKYHA : 104
ARALY    : -----MDTMRISGVSTGAEILVQCNSLSSLVSRRC-DDGRLRTRMFPARSR----NWRPSP-----TRRSSLSVKSV-SSEPKAKVTDAVLDSEQEVF-SSMNPFAPDAASVASSIKYHA : 103

                    *       140         *       160         *       180         *       200         *       220         *       240
SOLTU-HA : EFTPVFSPERFELPKAFFATTQSVRDSLLINWNATYDIYEKLNMKQAYYLSMEFLQGRALLNAIGNLELTGAFAEALKNLGHNLENVASQEPDAALGNGGLGRLASCFLDSLATLNYPAW : 207
SOLTU-HR : EFTPVFSPERFELPKAFFATAQSVRDSLLINWNATYDIYEKLNMKQAYYLSMEFLQGRALLNAIGNLELTGAFAEALKNLGHNLENVASQEPDAALGNGGLGRLASCFLDSLATLNYPAW : 207
SPIOL    : EFTPLFAPNDFSLPKAFFAAAQSVRDSLIINWNATYAHYEKMNMKQAYYLSMEFLQGRALLNAIGNLELTDAYGDALKKLGHNLEAVACQERDAALGNGGLGRLASCFLDSLATLNYPAW : 230
SOLTUb   : EFTPSFSPEKFELPKAYYATAESVRDTLIINWNATYEFYEKMNVKQAYYLSMEFLQGRALLNAIGNLGLTGPYADALTKLGYSLEDVARQEPDAALGNGGLGRLASCFLDSMATLNYPAW : 210
POPTRa   : EFTPLFSPEHFDLPKAFVATAESVRDSLIINWNATYKYYEKMNVKQAYYLSMEYLQGRALLNAIGNLELSGAYADALRKLGHELEDVAGQEPDAALGNGGLGRLASCFLDSLATLNYPAW : 205
RICCOb   : EFTPSFSPEHFELPKAFVATAESVRDSLIINWNATYDYYAKIHVKQAYYLSMEFLQGRALLNAIGNLELSGAYAEALKKLGHNLEDVARQEPDAALGNGGLGRLASCFLDSLATLNYPAW : 210
WHEAT    : DFTPLFSPEHSSPLKAYHATAKSVFDSLIINWNATYDYYNKVNAKQAYYLSMEFLQGRALTNAIGNLELTGQYAEALKQLGQNLEDVASQEPDPALGNGGLGRLASCFLDSMATLNYPAW : 205
ORYSJa   : EFTPVFSPEHFSPLKAYHATAKSVLDTLIMNWNATYDYYDRTNVKQAYYLSMEFLQGRALTNAVGNLELTGQYAEALQQLGHSLEDVATQEPDAALGNGGLGRLASCFLDSLATLNYPAW : 209
ORYSJb   : EFTPVFSPEHFSPLKAYHATAKSVLDTLIMNWNATYDYYDRTNVKQAYYLSMEFLQGRALTNAVGNLELTGQYAEALQQLGHSLEDVATQEPDAALGNGGLGRLASCFLDSLATLNYPAW : 209
CUCMA    : EFTPLFSPDRFDLPKAYFATAQSVRDALIINWNETYELYEKLNVKQAYYLSMEFLQGRALLNAIGNLELTGAYAEALSKLGCELENVACQEPDAALGNGGLGRLASCFLDSLATLNYPAW : 217
IPOBA    : EFSPAFSPERFELPKAYFATAQSVRDALIVNWNATYDYYEKLNMKQAYYLSMEFLQGRALLNAIGNLELTGEYAEALNKLGHNLENVASKEPDAALGNGGLGRLASCFLDSLATLNYPAW : 200
VICFA    : EFTPLFSPEKFELPQAFIATAQSVRDALIINWNATYDYYEKLNVKQAYYLSMEFLQGRALLNAIGNLELTGPYAEALSQLSYKLEDVAHQEPDAALGNGGLGRLASCFLDSLATLNYPAW : 225
POPTRb   : EFTPLFSPERFELPKAYYATAQSVRDALIINWNSTYESYERLNAKQAYYLSMEFLQGRALLNAIGNLELTGAYAEALSKLGHSLENVACQEPDAALGNGGLGRLASCFLDSLATLNYPAW : 180
RICCOa   : EFTPSFSPEQFELPKAFFATAQSVRDSLIINWNSTYEYYEKLNVKQAYYMSMEFLQGRALLNAVGNLELTGAYAEALTKLGHNLENVARQEPDAALGNGGLGRLASCFLDSLATLNYPAW : 217
GOSHI    : EFTPLFSPEKFDPPKAFFATAQSIRDALIINWNATYDYYERLNVKQAYYLSMEFLQGRALLNAIGNLGLTGAYAEALSKLGHNLENIASQEPDAALGNGGLGRLASCFLDSLATLNYPAW : 169
ARATH    : EFTPLFSPEKFELPKAFFATAQSVRDALIMNWNATYEYYNRVNVKQAYYLSMEFLQGRALSNAVGNLGLNSAYGDALKRLGFDLESVASQEPDPALGNGGLGRLASCFLDSMATLNYPAW : 224
ARALY    : EFTPLFSPEKFELPKAFFATAQSVRDALIMNWNATYEYYNRVNVKQAYYLSMEFLQGRALSNAVGNLGLNSTYGDALKRLGFDLESVVSQEPDPALGNGGLGRLASCFLDSMATLNYPAW : 223

                    *       260         *       280         *       300         *       320         *       340         *       360
SOLTU-HA : GYGLRYKYGLFKQRITKDGQEEVAEDWLEIGSPWEVVRNDVSYPIKFYGKVSTGSDGKRYWIGGEDIRAVAYDVPIPGYKTRTTISLRLWSTQVPSADFDLSAFNAGEHTKACEAQANAE : 327
SOLTU-HR : GYGLRYKYGLFKQRITKDGQEEVAEDWLEIGSPWEVVRNDVSYPIKFYGKVSTGSDGKRYWIGGEDIKAVAYDVPIPGYKTRTTISLRLWSTQVPSADFDLSAFNAGEHTKACEAQANAE : 327
SPIOL    : GYGLRYKYGLFKQMITKDGQEEVAENWLEIANPWELVRNDVSYSIKFYGKVVSGSDGRSHWTGGEDIRAVAYDVPIPGYQTKTTINLRLWCTTVSSEDFDLSAFNAGEHAKANEARANAE : 350
SOLTUb   : GYGLRYQYGLFKQLITKDGQEEVAENWLEMGNPWEIVRNDISYPVKFYGKVIEGADGRKEWAGGEDITAVAYDVPIPGYKTKTTINLRLWTTKLAAEAFDLYAFNNGDHAKAYEAQKKAE : 330
POPTRa   : GYGLRYKYGLFKQLITKDGQEEVAENWLEMGNPWEIVRNDVSYSVKFYGEVISKPDGSKEWIGGENITAVAYDVPIPGYKTKTTINLRLWSTKVAPNEFDLRAYNAGDHAKACAALKNAE : 325
RICCOb   : GYGLRYKYGLFKQLITKDGQEEVAESWLEMGNPWEIVRNDVSYPVKFYGKVILKPDGSKEWIGGENILAVAYDVPIPGYKTKTTINLRLWSTKVSPQEFDLSAFNTGDHAKAYAAVKNAE : 330
WHEAT    : GYGLRYRYGLFKQIIAKDGQEEVAENWLEMGNPWEIVRNDVSYPVKFYGKVVEGTDGRKHWIGGENIKAVAHDVPIPGYKTKTTNNLRLWSTTVPSQNFDLGAFNAGDHAKANEAHLNAE : 325
ORYSJa   : GYGLRYKHGLFKQIITKDGQEEVAENWLEMGNPWEIVRTDVSYPVKFYGKVVEGTDGRMHWIGGENIKVVAHDIPIPGYKTKTTNNLRLWSTTVPSQDFDLEAFNAGDHASAYEAHLNAE : 329
ORYSJb   : GYGLRYKHGLFKANHTKDGQEEVAENWLEMGNPWEIVRTDVSYPVKFYGKVVEGTDGRMHWIGGENIKVVAHDIPIPGYKTKTTNNLRLWSTTVPSQDFDLEAFNAGDHASAYEAHLNAE : 329
CUCMA    : GYGLRYKYGLFKQKITKDGQQEAAENWLEMAT-LGIVRNDIRYPIKFYGKVVVGSDGKKNWIGGEDIEAVAYDVPIPGYKTKNTISLRLWSTKAPTEDFDLAAFNAGEHSRASEALASAE : 336
IPOBA    : GYGLRYKYGLFKQRITKDGQEEVAEDWLELGNPWEIIRMDVSYPVKFFGKVITGSDGKKHWIGGEDILAVAYDVPIPGYKTRTTISLRLWSTKVPSEDFDLYSFNAGEHTKACEAQANAE : 320
VICFA    : GYGLRYKYGLFKQRITKDGQEEVAEDWLEMGNPWEIVRNDVSYPVRFYGKVVSGSDGKKHWVGGEDIKAVAHDVPIPGYKTRSTINLRLWSTKAASEEFDLNAFNSGRHTEASEALANAE : 345
POPTRb   : GYGLRYKYGLFKQQITKDGQEEVAEDWLEMGNPWEILRNDISYPIKFYGKVVSGSDGKKHWIGGEDIKAVAYDVPIPGYKTKTTINLRLWSTKAPSEDLDLYAFNAGDHTKAYEALSNAE : 300
RICCOa   : GYGLRYKYGLFKQRITKDGQEEVAEDWLEMGNPWEIVRNDVAYPVKFYGKVVSGSDGRKHWIGGEDIKAVAYDVPIPGYKTKSTINLRLWSTKAPAEDLDLSAFNSGDHTKAYETLANAE : 337
GOSHI    : GYGLRYRYGLFKQHITKDGQEEVAENWLEMSNPWEIVRNDVAYPIKFYGKVLTDSDGKKHWIGGEDIQAVAYDVPIPGYETKTTINLRLWSTKAPSGDFDLSVFNSGKHTQAAEALYNAE : 289
ARATH    : GYGLRYKYGLFKQRITKDGQEEAAEDWLELSNPWEIVRNDVSYPIKFYGKVVFGSDGKKRWIGGEDIVAVAYDVPIPGYKTKTTINLRLWSTKAPSEDFDLSSYNSGKHTEAAEALFNAE : 344
ARALY    : GYGLRYKYGLFKQRITKDGQEEAAEDWLELSNPWEIVRNDVSYPIKFYGKVVFGSDGKKRWIGGEDIVAVAYDVPIPGYKTKTTINLRLWSTKAPSEDFDLSSYNSGKHTEAAEALFNAE : 343

                    *       380         *       400         *       420         *       440         *       460         *       480
SOLTU-HA : KICYILYPGDESEEGKILRLKQQYTLCSASLQDIISRFERRSGDRIKWEEFPEKVAVQMNDTHPTLCIPELMRILIDLKGLNWNEAWNITQRTVAYTNHTVLPEALEKWSYELMQKLLPR : 447
SOLTU-HR : KICYILYPGDESEEGKILRLKQQYTLCSASLQDIISRFERRSGDRIKWEEFPEKVAVQMNDTHPTLCIPELMRILIDLKGLNWNEAWNITQRTVAYTNHTVLPEALEKWSYELMQKLLPR : 447
SPIOL    : KICSVLYPGDESMEGKILRLKQQYTLCSASLQDIISQFERRSGEHVNWEEFPEKVAVQMNDTHPTLCIPELMRILIDVKGLAWKEAWNITQRTVAYTNHTVLPEALEKWSFELMQSLLPR : 470
SOLTUb   : KICYVLYPGDESLEGKTLRLKQQYTLCSASLQDIIARFEKRSGNAVNWDQFPEKVAVQMNDTHPTLCIPELLRILMDVKGLSWKQAWEITQRTVAYTNHTVLPEALEKWSFTLLGELLPR : 450
POPTRa   : KICYILYPGDESTEGKILRLKQQYTLCSASLQDIIAHFERRSGKPVNWENFPDKVAVQMNDTHPTLCIPELIRILIDLKGLSWKESWYITQRTVAYTNHTVLPEALEKWSLDLLQKLLPR : 445
RICCOb   : KICYILYPGDESIEGKTLRLKQQYTLCSASLQDIIAHFERRSGEAVKWENFPDKVAVQMNDTHPTLCIPELIRILMDVKGLSWDKAWNITRRTVAYTNHTVLPEALEKWSLDLLQELLPR : 450
WHEAT    : KICHVLYPGDESSEGKILRLKQQYTLCSASLQDIISRFESRAGDSLNWEDFPSKVAVQMNDTHPTLCIPELMRILMDIKGLSWNEAWSITERTVAYTNHTVLPEALEKWSLDIMQKLLPR : 445
ORYSJa   : KICHVLYPGDESPEGKVLRLKQQYTLCSASLQDIIARFERRAGDSLSWEDFPSKVAVQMNDTHPTLCIPELMRILIDVKGLSWNEAWSITERTVAYTNHTVLPEALEKWSLDIMQKLLPR : 449
ORYSJb   : KPHY---------------------------RDIIARFERRAGDSLSWEDFPSKVAVQMNDTHPTLCIPELMRILIDVKGLSWNEAWSITERTVAYTNHTVLPEALEKWSLDIMQKLLPR : 422
CUCMA    : KICHVLYPGDDSFEGKVLRLKQQYTLCSASLQDIVARFERRSGANKKWGEFPEKVAVQMNDTHPTLCIPELMRILLDLKGLSWEEAWNITQRTVAYTNHTVLPEALEKWSFELMQKLLPR : 456
IPOBA    : KICYILYPGDESIEGKILRLKQQYTLCSASLQDIIARFERRSGEYVKWEEFPEKVAVQMNDTHPTLCIPELIRILIDLKGLSWKEAWNITQRTVAYTNHTVLPEALEKWSYELMEKLLPR : 440
VICFA    : KICYILYPGDESIEGKTLRLKQQYTLCSASLQDIIARFERRSGASVNWEDFPEKVAVQMNDTHPTLCIPELMRILIDIKGLSWKDAWNITQRTVAYTNHTVLPEALEKWSMDLMEKLLPR : 465
POPTRb   : KICHVLYPGDDSLEGKILRLKQQYTLCSASLQDIISCFERRSGSNIDWEKFPEKVAVQMNDTHPTLCIPELMRILIDLKGLSWKEAWNITQRTVAYTNHTVLPEALEKWSLELMQKLLPR : 420
RICCOa   : KICHILYPGDDSVEGKILRLKQQYTLCSASLQDIIVRFERRSGSHVKWEEFPEKVAVQMNDTHPTLCIPELMRILMDLKGLSWKEAWNITQRTVAYTNHTVLPEALEKWSLDLMQKLLPR : 457
GOSHI    : KICYVLYPGDESLEGQILRLKQQYTLCSASLQDIIARFERRSGAKVKWDEFPDKVAVQMNDTHPTLCIPELMRILIDVKGLSWKEAWNITQRTVAYTNHTVLPEALEKWSLELMQKLLPR : 409
ARATH    : KICFVLYPGDESTEGKALRLKQQYTLCSASLQDIVARFETRSGGNVNWEEFPEKVAVQMNDTHPTLCIPELMRILMDLKGLSWEDAWKITQRTVAYTNHTVLPEALEKWSLELMEKLLPR : 464
ARALY    : KICFVLYPGDESNEGKALRLKQQYTLCSASLQDI-CQLGR----------IPEKVAVQMNDTHPTLCIPELMRILMDLKGLSWEDAWKITQRTVAYTNHTVLPEALEKWSLELMEKLLPR : 452

                    *       500         *       520         *       540         *       560         *       580         *       600
SOLTU-HA : HVEIIEAIDEELVHEIVLKYGSMDLNKLEEKLTTMRILENFDLPSSVAELFIKPEISVDD-------DTE------------TVEVHDKVEASDKVVTNDEDDTGKKTSVKIEAAAEKDI : 548
SOLTU-HR : HVEIIEAIDEELVHEIVLKYGSMDLNKLEEKLTTMRILENFDLPSSVAELFIKPEISVDD-------DTE------------TVEVHDKVEASDKVVTNDEDDTGKKTSVKIEAAAEKDI : 548
SPIOL    : HVEIIEKIDEELVDTIVSEYGTDDPKLLMGKLNELRILENFHLPSSVASI-IKDKITCQV----------------------DEDKKIEISDEVDGLVVVEES--------------EEG : 553
SOLTUb   : HVEIIAMIDEELLHTILAEYGTEDLDLLQEKLNQMRILDNVEIPSSVLELLIKAEESAAD-----VEKAADEE---------QEEEGKDDSKDEETEAVKAETTNEEEETEVKKVEVEDS : 556
POPTRa   : HVEIIRMIDEELIHTIIAEYGTGDLDLLQHKLKQMRILDNIELPDSVLELLVKQEESS------------------------SVDSIKEVKVSDAETESTDEE----------QSEEQDT : 531
RICCOb   : HIEIIKMIDEELIHTIIAEYGMEDLDLLEQKLKEMRILDNVELPESVVQLLVKSDKSFAV-------ESVIEDI--------EVEDSEQETKSTAEDKHTEEKKDEEEEEEEEEEEEEEG : 555
WHEAT    : HVEIIETIDEKLMNNIVSKYGTADISLLKQKLKDMRILDNVDLPASVAKLFIKPKEKTGK------------LLVQSLESIAEGDEKTESQEEENILSETAEKKGGSDSEEAPDAEKEDP : 553
ORYSJa   : HVEIIEKIDGELMNIIISKYGTEDTSLLKKKIKEMRILDNIDLPDSIAKLFVKPKEKKES-----PAKLKEKLLVKSL----EPSVVVEEKTVSKVEINEDSEEVEVDSEEVVEAENEDS : 560
ORYSJb   : HVEIIEKIDGELMNIIISKYGTEDTSLLKKKIKEMRILDNIDLPDSIAKLFVKPKEKKES-----PAKLKEKLLVKSL----EPSVVVEEKTVSKVEINEDSEEVEVDSEEVVEAENEDS : 533
CUCMA    : HVEIIELIDEELVRSIISEYGMEDKVLLRGKLKELRVLENVDLPAAYSDIFIEPEESSII----------------------TSAEGHVISNSVDDKNSSKLAKSVDGDETVKGNDELES : 554
IPOBA    : HIEIIEMIDEQLINEIVSEYGTSDLDMLEKKLNDMRILENFDIPSSIANLFTKPKETSIV----------------------DPSEEVEVSGKVVTESVEVSDKVVTES-EKDELEEKDT : 537
VICFA    : HVEIIEMIDEELIRTIIAEYGTADSDLLDKKLKEMRILENVELPAEFADILVKTKEATDISSEEVQISKEGGEEEETSKEGGEEEEEKEVGGGREEGDDGKEDEVEKAIAEKDGTVKSSI : 585
POPTRb   : HVEIIELIDEELICTIVSEYGTEDSDLLEKKLKEMRILENVDLPSAFAELIVKPKQSSVE-------TKRANDFEEETKRANDLEEETNLEEETKRANDFEEEMELVD--EKDESKSKVT : 531
RICCOa   : HVEIIEMIDEELIRTIVSEYGREDLDLLNKKLKEMRILENVDLPDAFADLIIKTKESSAA------STTKEPE---------DADDEIKLVNEKDELESKEESENKDEAERKDELENKNT : 562
GOSHI    : HMEIIEMIDEELIRTIVSEHDKADSNLLEKKLKQMRILENVELPAAFSDLLVKPKKSPVA-----VPSDEFGESE-------EEEEEEEEAEAEAEAEEEKEEEKLKPAGGKIKSVKEGT : 517
ARATH    : HVEIIEKIDEELVRTIVSEYGTADPDLLEEKLKAMRILENVELPSAFADVIVKPVNKPVT----------------------AKDAQNGVKTEQEEEKTAGEE----------------- : 545
ARALY    : HVEIIEKIDEELVRTIVSEYGTADPDLLEEKLKAMRILENVELPSAFADVIVKPENKPVT----------------------AKDAQNGVKTEQEEEKIAGEE----------------- : 533
```

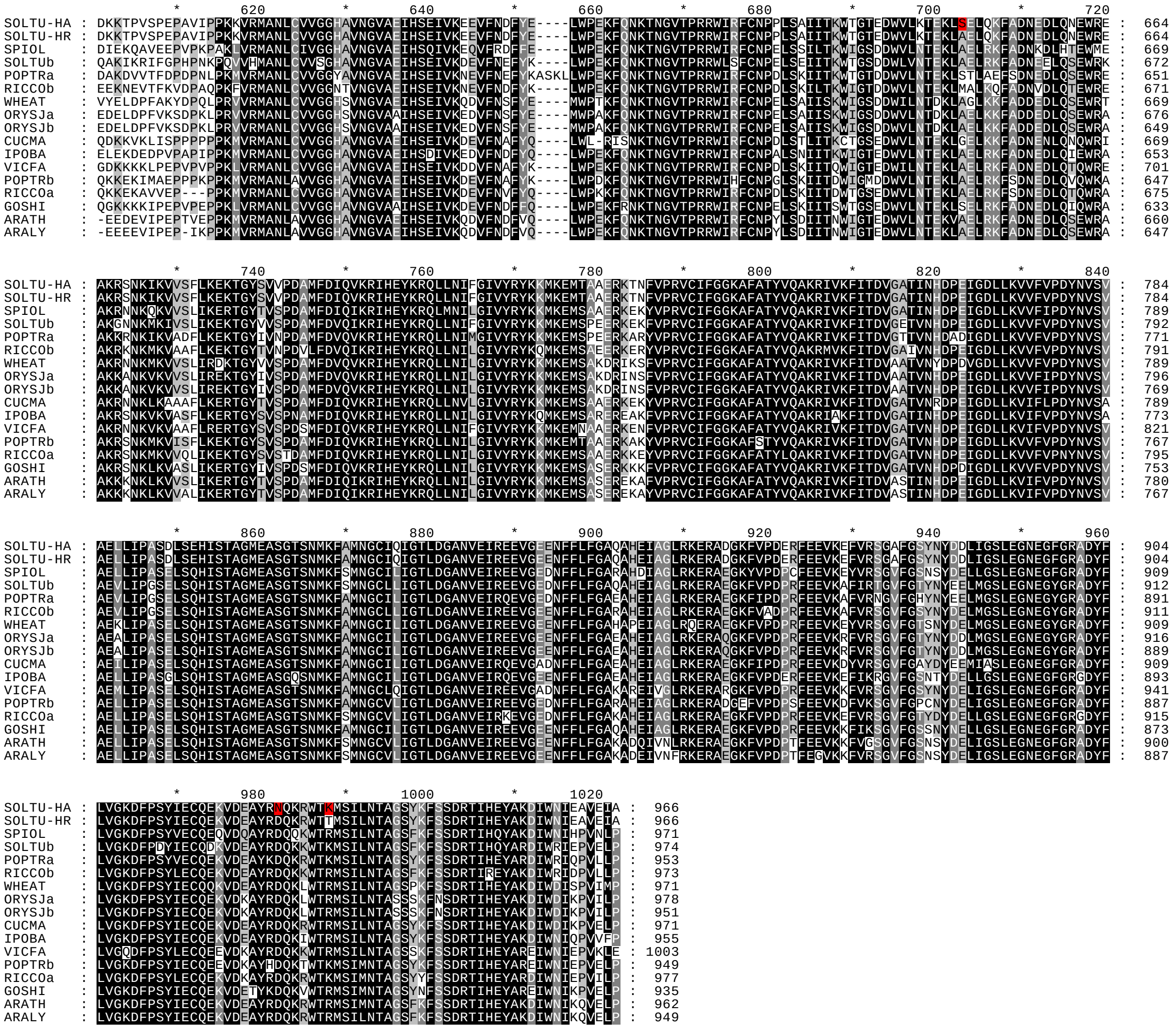

Figure S2 Alignment of plant PHO1a deduced protein sequences. *Solanum tuberosum*: SOLTU-HA (Pho1a-H_A, this paper), SOLTU-HR (P04045); *Spinacea oleracea*: SPIOL (O24363); *Populus trichocarpa:* POPTRa (B9HXL0), POPTRb (B9H0D3); *Ricinus communis*: RICCOa (B9SJB6); RICCOb (B9RCW0); *Triticum aestivum*: WHEAT (B2LXU4); *Oryza sativa* ssp. *japonica*: ORYSJa (B3IYE3), ORYSJb (Q9AUX8); *Cucurbita maxima*: CUCMA (B2DG13); *Ipomea batatas*: IPOBA (P27598); *Vicia faba*: VICFA (P53536); *Gossypium hirsutum*: GOSHI (D2D337); *Arabidopsis thaliana*: ARATH (Q9LIB2); *Arabidopsis lyrata*: ARALY (D7LNX4). The six amino acid changes in Pho1a-H_A versus Pho1a-H_R are highlighted red.
