# Supplementary material for: SNPs in Genes Functional in Starch-Sugar Interconversion Associate with Natural Variation of Tuber Starch and Sugar Content of Potato (Solanum tuberosum L.)
Source: G3 (Bethesda). 2014 Jul 31;4(10):1797–811. doi: 10.1534/g3.114.012377 (PMC4199688; doi:10.1534/g3.114.012377)
Supplement: Supporting Information [file supp_g3.114.012377_FileS1.pdf]

File S1

Amplified potato genomic fragments including SNP positions, nucleotide alleles and identifications.

Sequences were retrieved from pseudomolecules (v4.03) at http://potato.plantbiology.msu.edu/cgi-bin/gbrowse/potato/. Primer sequences are underlined. SNP numbering starts with A = 1 in the ATG initiation codon in the genomic locus PGSC0003DMG*********.

1. Glucan water dikinase, *GWD* (PGSC0003DMG400007677)

chr05:9827209..9827979

```
ATAAAAGTCAAAGCAAAGAAGAGCCTCTTCATGTAACAAAGAGTGAAATACCTGATGACCTTGCCCAAGCACAAGCTTACATT
AGGTGGGAGAAAGCAGGAAAGCCGAACTATCCTCCAGAAAA
                    3058              3072                3089
GCAAATTGTAAATGCTGAAC(T/C)TTTCTTACAGTTT(A/T)TGTAGTGTGTGTATAT(G/
    3092            3104         3113
C)TC(T/C)AGGCGCATAAA(T/G)CTGGGTAT(T/G)CTGTCTTCTATAGATGTGAGACTT
       3145
TGATCAA(G/A)CATTGTTTTATTAACAGGAAGAACTCGAAGAAGCAAGAAGAGAATTGCAA
                                                  3246
CTTGAGCTTGAGAAAGGCATTACCCTTGATGAGTTGCGGAAAAAGATTAC(A/C)AAAGGGG
     3259          3269           3280  3281           3291
AGATA(A/G)AAACTAAGG(C/T)GGAAAAGCAC(G/C)(T/A/C)GAAAAGAAG(C/T)TC
    3298                                         3339
TTTT(G/T)CCGTTGAAAGAATCCAAAGAAAGAAGAGAGACTTTGGGCA(G/T)CTTATTAA
       3355                3372                  3389
TAAGTAT(C/A)CTTCCAGTCCTGCAGT(A/G)CAAGTACAAAAGGTCT(/C)GGAAGAAC
  3400                   3419  3421
CA(C/G)CAGCCTTATCTAAAATTA(A/G)G(C/A)TGTATGCCAAGGAGAAGGAGGAGCAG
    3452             3465    3469                  3487
ATTG(A/G)TGATCCGATCCT(A/T)AAT(G/A)AAAAGATCTTTAAGGTC(G/A)ATGATG
          3504                                            3550
GGGAGCTACT(G/T)GTGAGTGCTAACTTATTCTTGACTGTAACCACTAATCTCAGAAGC(T
          3558                                          3600
/A)AGTTTGA(A/G)TGCATATTGAGAATCTTCCATTGTCTCCACATTTTAAGGTA(C/G)T
    3606
GGTA(G/T)CAAAGTCCTCTGGGAAGACAAAAGTACATCTAGCTACAGATCTGAATCAGCCAATTACTCTTCACTGGGCATTA
TCC
```

2. Phosphoglucan water dikinase, *PWD* (PGSC0003DMG400016613)

chr09:60571111..60571981

```
GGTCTGATGATCTATCTGATTGCAGTTTATAGTGACCAAGGTGCACCAGCATCATTTAACGT
      10503                                       10543  10547
TCCTGC[A/T]GGAGCAGTTATTCCATTTGGTTCCATGGAAACGGCATTG[G/A]AAA[T/C
                             10576
]GAACAAGTTAATGGAGACCTTCACATTG[C/T]TTGTCGAACAGATAGAAACAGCTGAAAT
                        10629                           10657
TGATGGCGGTGAACTTGATAAACA[T/C]TGTGAGGATCTCCAGAAGTTAATATCT[T/A]C
                              10689         10700 10701
TCTATTGCCTGGACAAGATGTCATTGAAAG[C/G]TTGGGAGAAG[T/C][A/G]TTTCCCG
                                     10746           10758
GTAATGCACGTTTAATAGTGCGTTCAAGTGCTAATGT[C/T]GAGGACTTGGC[G/A]GG[G
10761      10770  10773
/A]ATGTCAGC[T/A]GC[T/C]GGACTTTATGATTCAATTCCTAATGTTAGCCCTTCAGAT
  10815
CC[G/A]ATAAGGTTTGGACATGCTGTAGCCCGTGTTTGGGCCTCGTTGTATACTAGAAGAG
                                        10911   10916 10917
CAGTACTGAGCCGCAGAGCTGCTGGTGTGTCCCAGAAAGA[C/T]GCTA[C/A][A/G]ATG
  10923        10932                             10962
GC[C/T]GTGCTAGT[G/T]CAAGAAATGCTTTCACCAGATTTATCTTT[C/T]GTCCTCCA
                                                   11022
CACACTGAGCCCAACGGACAACAATCATAACTTCATTGAGGCTGAAATTGC[A/T]CCTGGA
                                   11064
CTCGGTGAAACACTCGCTTCAGGAACAAGGGGTAC[A/T]CCGTGGCGTCTATCTAGTGGTA
                         11112     11218
AATTTGACGACACAGTGCGCACACT[G/A]GCATT[C/T]GCCAACTTTAGTGAGGAGATG[
11140      11148 11151 11152
G/A]TTGTAGG[T/A]GG[C/T][A/C]ATTCCCCTGCTGACGGAGAAGTTATTCTCTTGACTGTCGATTATAGCAAGAAACC
TTTAACAATCGACCCCATTTTCAGACGTCAGCTTGGTCAGAGGCTTGGCGCTGTTGGTTTCTACCTAGAACGCAAGTTTGGTT
CTCCTCAAGATGTCG
```

3. Beta amylase 1, *BMY1* (PGSC0003DMG400001855)

Chr08:50594548..50595107

```
GCTACTGGAGCATGGTGACAGAATAGTAGCAGCAGGAGAAAGTATATACCAAGGAACTGGGGCTAAACTATCTGGAAAGGTAG
CTGGGATTCATTGGCATTACAATACTAGATCACATGCTGCA
                                     2533
GAGTTAACTTCAGGATATTATAATACAAGACACAGA(G/A)ATGGTTATCTACCTATAGCAC
           2565           2576       2583
GTATGTTAGC(G/A)AAACATGGTG(C/T)TGTACT(T/G)AACTTTACATGTATGGAAATG
         2613            2625                        2649
AGGGATGG(T/G)GAACAGCCCCA(G/A)AGTGCAAACTGTTCACCAGAAGG(C/A)TTAGT
   2657              2671              2686        2694
TC(A/G)ACAAGTTAAAACT(G/A)CAGCTAGAACTGCT(A/G)AAGTAGA(A/G)CTTGCT
                                                   2751
GGAGAAAATGCTCTAGAAAGGTATGATGGAGGAGCATTTTCTCAAGTTTT(G/A)GCAACAA
         2767                                            2811
GCATGTCA(A/G)ATTCTGGAAATGGATTGAGTGCATTTACATTCTTGCGAATGAA(C/T)AAACGGTTGTTTGAGCCAGAAA
ATTGGCGGAATCTAGTGCAATTTGTGAAGAGCATGTCTGAAGGAGGTCGAAATGCTAGCCTTCCAGAGTGTGACTCAAGCAGG
ACAGACCTCTATGTAA
```

4. Starch phosphorylase, chloroplastic/amyloplastic, *PHO1b* (PGSC0003DMG400028382)

Chr05:350160..351337

```
TGTTGCAAGAAAAGCTAAACCAAATGAGGATTCTGGATAATGTTGAAATACCAACTTCTGTTTTGGAGTTGCTTATAAAAGCC
GAAGAAAATGCTGCTGATGTCGAAAAAGCAGCAGATGAAGAACAAGAAGAAGAAGGTAAGGATGACAGTAAAGATGAGGAAAC
TGAGGCTGTAAAGGCAGAAACTACGAACGAAGAGGAGGAAACTGAGGTTAAGAAGGTTGAGGTGGAGGATAGTCAAGCAAAAA
TAAAACGTATATTTGGGCCACATCCAAATAAATCACAGGTGGTTCACATGGCAAATCTATGTGTAGTTAGCGGGCATGCAGTT
AACGGTGTTGCTGAGATTCATAGTGAAATAGTTAAAGATGAAGTTTTCAATGAATTTTACAAGGTACGATGATTATCACACGG
CTAGAAGTGGCAGATATCA

                                                        3882
TTTTCAGTTCTCCTCTTTCAATGTTGTTGTTTTGATTGATCCAAACTCTTTGTTC(A/C)TC
                                              3931   3934
GACAGTTATGGCCAGAGAAATTCCAAAACAAGACAAATGGTGTGAC(A/G)CC(A/T)AGAA
                    3958                           3982      3990
GATGGCTAAGTTTCTGTAA(T/C)CCAGAGTTGAGTGAAATTATAAC(A/C)AAGTGGA(C/
                              4018                   4038
T)AGGATCTGATGATTGGTTAGTAAACAC(T/A)GAAAAATTGGCAGAGCTTC(G/A)AAAG
       4049    4052                        4076           4088
GTAAAC(G/A)AG(T/C/G)TGTATCTGACTGTGATCTTATT(G/A)ATTCCTAAACC(G/A
                   4106   4109     4112          4122
)TCGTCAACAGCTTTCAT(A/G)AA(C/G/A)GG(A/G)TTATTTCCT(T/C)TAGTCCTTC
                                                 4181
TATGTGGAAAGAGATCTTTATAAACATCATAAGAACATGTGAAATGTAT(G/A)TACTAA(C
4188         4198        4206   4207
/T)TTCATGCAT(T/G)TAACTGT(T/C/G)(G/T/A)AACAGTTTGCTGATAACGAAGAAC
     4236            4248          4258
TCCA(G/A)TCTGAGTGGAG(G/A)AAGGCAAAA(G/A)GAAATAACAAAATGAAGATTGTC
                     4302                4319 4320
TCTCTCATTAAAGAAAAAAC(A/T)GGATACGTGGTCAGTC(C/G)(C/T)GATGCAATGTT
                   4350      4356         4365
TGATGTTCAGATCAAGCG(C/T)ATCCA(T/C)GAGTATAA(A/G)AGGCAGCTATTAAATA
  4383      4389              4404   4407      4413
T(A/T)TTTGG(A/T)ATCGTTTATCGCTA(T/C)AA(G/A)AAGAT(G/T)AAAGAAATGA
        4431                         4457                4437
GCCCTGA(A/T)GAACGAAAAGAAAAGTTTGTCCCTC(G/A)AGTTTGCATATTTGG(G/A)GGAAAAGCATTTGCTACATAT
GTTCAGGCCAAGAGAATTGTAAAATTTATCACTGATGTAGGGGCAACAGTCAACCATGATCCCGAGATTGGTGATC
```

5. Soluble starch synthase I, *SssI* (PGSC0003DMG402018552)

Chr03:45608638..45609659

```
GGATACTCATGGGAAATAACAACTCCTGAAGGGGGATATGGGCTACATGAGCTGTTGAGCAGTAGACAGTCTGTTCTTAATGG
TATGCGTGTTAGATGATTCCTTATGAGTGCTTTCTGATATTCTTCTTTTCCTCTTACTCGAATTGTAATTTGGTAGGATATAT
GTTGATTGACGAGGCTTATGTACCAGCATTAGCTTCATTTCTAGTCAAGTCCTAACTTTTGTTAAAACATGACCAGAGCCAAG
GCTAATTGTATTAATGACCTTCACCATGCATTTCTGTTGTCTCTGAACTTTAGAAATTACTAGTTAATCTATCTCTACTTTTA
TGCAGGAATTACTAATGGAATAGATGTTAATGATTGGAACCCGTCGACAGATGAGCATATTGCTTCGCATTACTCCATCAATG
ACCTCTCCGGAAAGGTTATTAAACTTCTCAGTTTCACAATTTTGCAAATTAAGTGATAAAGTGCAGCTAGAGAAAAGGACTAT
TAGCATATTTGGCCAATCTTCCATAAATTGCTTATTTTGGGAAGTGTTTTTTATTAGAAGTACTTTTGGAGAATAGTAGTTTG
TATTTGGCTAATCAATTTGAAAAAAAGCCTTTGTCAATATTAGAGCAGTAATTTGTGCTTGACAAAGGTTCCAATAGTGCTTC
GAGGGGAAAAGCTATTTT
                                         5803
TTTCTTCCAACTTCAGCTACTACTCAAAAGTCAAAAGCAC(indelA)GCTTGGCTGAACAG
       5824              5839             5850        5858
ACAATA(C/T)GTCTACAAAAGCTT(C/A/T)TTAACTGCTG(C/T)GAAATTC(A/G)ATC
          5871     5877   5880
CAAATGAGG(A/G)TTCCT(C/T)TA(G/A)AGGATTGTCCATAATCCATTAAAAGGGAACA
                                                    5964
GTCCAAAGGATATCATAGCATTTACTTACTTTTCATGTTTATTCTACATGCA(G/A)TTGTG
                                6001         6010
CACAAACTCTCTTTTTCTCTGTTCTCTACAT(A/G)CCACTGCT(C/T)AAATTGTCTCTGCTAACTTTCCATTTAATTATAC
AGGTTCAGTGCAAGACTGATCTGCAAAAGGAACTGGGCCTTCCAATTCGACCTGATTG
```

6. Phosphoglucomutase, *PGM-3* (not annotated)

Chr03:41510113..41509054

```
ATGGCTATGGAGAGTGCATTGACATCCACACGAGTTTCAATTCCATCGTTGTGTTCTGGGAT
                              93
CAGTTCATCTCATCATCACCATAGATCCTT[C/A/T]TCATTTCTCAATTTCCCCAAATTGT
       125
CTTCAT(T/G)CAAATACTCTTTTCGAACAATTTCACCTGTTCCTTTCGTTGTCAGTGCATCCTCTGTTTCTCCATCATCTCC
TTCTACATCTGTTGCCCAATCTCAGGATCTCAAGGTATCTA
   241    242             254
AT(T/A/C)(G/A)CTTCCAAATAT(C/T)ACTATATAACGTGTATATTTTTATGTGATTTA
                                  320                   339
ATGGAAAATTTGATTTACTTGAATGCCAGATTA(A/T)ATCAGTTCCTACCAAGCC(T/A)ATTGAAGGTCAAAAGACCGGTA
CCAGTGGTCTACGCAAAAAGGTGCCTTTTCTTTTGGTTTTA
      408  409  410  411   413                          440
TTAAA(A/T)(T/A)(T/A)(T/A)T(A/T)CACTTTGTTTGGATGATTGTTACCCG(C/T)
                          467  468
TGTATTGTATTGTTAGTTCAAATACG(A/G)(T/C)GTTTATTTTGATTGTTATATAAATTTGTTGTTTAGTATCATTTAAAT
TCATTTTCATTGTTAGCTACAAAAAGTCCCATTTTGTGTAAGGACCAATTTGGTGTGATTGCCTTGGTTACTTTTAATATTTT
CTTCTCATTTATCACATTTTAATGTTTTATCCTTTACCCTATTTTTTCGTAGTAGCTACATCCCGTGCCTTACTTTTCTCGTA
GGCTTATCAGACATATCGCAATGACGGAGAATGATACAATCTATCCAAATGTTGTATTTATTAAAATAATATTGTACGATACA
ATACAACACAATATGATACATAATGAAATAATACATAACAGCCCTCCAAGCAGAGTGAACTCCGCCTGCGTGAAGGTGTGCCA
AAATAAATAGGAAAACCTCTCTACCACTTAAGGTAGGGTTAAGGCTGCGTATATTTTACCTTCCTCAGACCTCACTTGTGCGA
TTACACTGGTTTGTTATTGTTGTTGGTTTATTAAATTTTTTATTGCTTACTGTTCATTTGGATGACTTCTGCTTCTTTTGTCT
ATAGGTTAAAGTGTTTATGCAGGACAATTACCTTGCCAATTGGATAC
```

7. ADP-glucose pyrophosphorylase S, *AGPaseS* (PGSC0003DMG400000735)

Chr01: 86095512..86094580

```
GGAGATGGATTTGTCGAGGTTTGAGACTATACTTTTGAAATTCTTGAAGGAAACTGGATTAGTCTTGCTAGATATCACAGCTG
AGCTAATTAATATGAGTAAATTTGTTGGTCCTCCAAGTGAT
                                                1259      1265
TTGTTCACACAAAATCTGTAACAATTTCGGATGATTTTTTTCATTGTT(T/C)TTCTG(A/C
   1268         1276        1284  1286
)TT(A/T)CTTGCTT(C/A)GTTTTAC(T/C)G(A/G)CTGAAAGGAATTCCGTCATTAAAC
                          1336            1348
AGGTACTAGCTGCAACTCAGACACC(C/T)GGGGAAGCAGG(A/G)AAAAAATGGTTTCAAG
                           1392                    1411
GAACAGCAGATGCTGTTAGAAAATTTA(T/C)ATGGGTTTTTGAGGTTTGT(C/T)TCTACA
        1426                          1456 1457 1459
ACTCTTTA(A/G)GACACACTAATCCACCACTGCTCTTCTGT(T/C)(G/)A(C/)CTGATA
                 1482                   1501       1509
TAATTTCTGAAAAAATG(A/T)TTCATTATTTTTTCCTGA(T/A)TCAGTTA(G/)TATGTAC
   1520          1529                 1546 1547
AGA(T/C)ATATAGTA(C/T)TTTTGTGTTTACAAGG(T/C)(G/A)ACCAATTCTTGCAGG
  1564                          1592                    1612
A(C/T)GCTAAGAACAAGAATATTGAAAATATC(G/C)TTGTACTATCTGGGGATCA(T/C)CTTTATAGGATGGATTATATG
GAGTTGGTGCAGGTATGTCCACTTTAACTACCTATGCACTC
               1689
CTCATTCTTTGTTT(A/G)CTTTTTTTTTTTTTTTTTTTAAACAACAAAGTTTCTGACTGCCTCCGATCTTCAAACAGAACCA
TATTGACAGGAATGCTGATATTACTCTTTCATGTGCACCAGCTGAGGACAGGTTTGTTTAAATTCCAATAAGAAACAACTAGT
AATGGTTAAGTGAATTGAGTGAGATCAGAAACAATATGGAGAAAGAGTGCTTCTCCTTTAATATTCGTATGCATTACTTCGAA
GTAAAAACATCACCACATTTCTTTATACCTTATTGTGATTATGTTAAGCTGAGATTTAAGCTTTACTTATCAGCCGAGCATCA
GATTTTGGGCTGGTCA
```

8. Invertase, *INV-8/2* (PGSC0003DMG400004790)

Chr08:52703547..52704642

```
GTTCTCATCCCACCACCCGGAATTGGGCTCAAGGACTTCCGTGACCCCACCACCGCGTGGACCACACCGGAAGGCAAATGGCG
TATTACTATTGGTTCAAAGATTAATAAAACCGGAATCTCAT
                                      2065         2075  2076
TGGTCTATGACACAATTGATTTTAAAAAATTTGAATT[G/A]TTGAAGGGT[G/A][C/T]G
                                       2116
TTACATGGTGTACCGGGTACGGGTATGTGGGAATGTGT[G/A]GATTTTTACCCGGTTTC[T
2134                                        2175       2182
/G]AAAATTGTTGAAAATGGGCTTGACACTTCAGAAAATGGGC[C/A]TGCAGT[G/A]AAA
           2197
CATGTTTTGAA[G/A]TCCAGTCTTGACGATGATCGAAATGACTATTATGCCCTTGGAACTT
            2255                 2272
ATGATGCAGGG[G/A]CAGGAAAATGGGTACC[T/A]GATAATCCAATAATTGATGTTGGTA
     2302                  2320
TTGG[G/A]TTAAGATATGATTATGG[T/C]AATTTTTATGCATCAAAAACATTTTATGATC
                  2369  2371
AAGAAAAAAAGAGAAGA[G/A]T[T/A]CTTTGGGCTTGGATTAAAGAAACTGATAGTGAAG
                   2425
CTGCTGATATTTGCAGGG[T/C]TTGGGCCTCACTTCAGGTATCTCTTTTTTAGCGTATCATTTAGATTTTGTCCTTATTTTT
AAAAGTCGTGCAAGTAGATACATAATTTGAAGTGTGACAAGTAATCAAAAATCAGACAATGACAGAACGGCCTATGATTAAGA
ACTAAAAACTAAAACTTTATACAACTTTTACAATTCATTGAACAAATTGGTAACGTGTGATTTTTTATTATTTTATTTCGAAA
ACAGTCAATTCCAAGAACTATAAAGTATGATAAGAAGACAGGAAGCAACATAATTACATGGCCAGTGGCTGAGGTAGAGAATT
TGAGATCAAACAACAATGAATTCAACAAGGTTGTAGTTAAACCAGGTTCAATTGTCCCACTAGAAGTTGGTTCTGCCACTCAG
GTTAGTACATTTTGTCGATTAAATTCAACTTATATACATTGACAATATATATACTTATATCGTTGAATTATTGTTACAGTTGG
ACATAATGGCTGAATTTGAAATAGACCAAAATGTGTTGAAGAAAGTAGATGGAAGTAATGCTACATATGATTGTATAAAGAGT
GGTGGATCTGGTGAACG
```
