# Supplementary material for: SNPs in Genes Functional in Starch-Sugar Interconversion Associate with Natural Variation of Tuber Starch and Sugar Content of Potato (Solanum tuberosum L.)
Source: G3 (Bethesda). 2014 Jul 31;4(10):1797–811. doi: 10.1534/g3.114.012377 (PMC4199688; doi:10.1534/g3.114.012377)
Supplement: Supporting Information [file supp_g3.114.012377_TableS1.pdf]

Table S1 Genes functional in plant starch-sugar interconversion as annotated in the potato genome and GeneBank accessions of corresponding cloned and characterized genes from potato and/or tomato. Loci highlighted green have been probed for association with tuber quality traits (this paper, BALDWIN *et al*. 2011, DRAFFEHN *et al*. 2010, KAWCHUK *et al*. 2008, LI *et al*. 2008, LI *et al*. 2005, LI *et al*. 2013, URBANY *et al*. 2011).

Enzyme/protein	Locus acronym [a]	Locus ID	GenBank accessions	Position (v4.03)
ADP-glucose pyrophosphorylase, large subunit	*AGPaseS-1/1*	PGSC0003DMG400009026	NM_001247048	chr01:62144770..62149140
ADP-glucose pyrophosphorylase, large subunit	*AGPaseS-1/2*	PGSC0003DMG400000735	X61187	chr01:86092270..86097270
ADP-glucose pyrophosphorylase, large subunit	*AGPaseS-7*	PGSC0003DMG400015952	NM_001247018	chr07:21365000..21370700
ADP-glucose pyrophosphorylase, small subunit	*AGPaseB-7*	PGSC0003DMG400031084	NM_001288195	chr07:51451500..51457400
ADP-glucose pyrophosphorylase, small subunit	*AGPaseB-12*	PGSC0003DMG400046891 [c]		chr12:1226600..1230220
1,4-alpha-glucan-maltohydrolase	*AMY-1*	PGSC0003DMG400010664	AJ849377	chr01:59361270..59364450
Alpha-amylase	*AMY-3*	PGSC0003DMG400020603	A21347	chr03:35999180..36002230
Alpha-amylase, AMY21	*AMY-4/1*	PGSC0003DMG400007974	M81682	chr04:68256940..68260200
Alpha-amylase, AmyZ	*AMY-4/2*	PGSC0003DMG400009891	M79328	chr04:71332570..71337470
Alpha-amylase	*AMY-5*	PGSC0003DMG401017626		chr05:5918690..5923240
Beta-amylase	*BMY-1*	PGSC0003DMG400000169		chr01:72104900..72114100
Beta-amylase	*BMY-7/1*	PGSC0003DMG400026199		chr07:47346060..47350380
Beta-amylase	*BMY-7/2*	PGSC0003DMG400026166		chr07:47402480..47403710
Beta-amylase	*BMY-7/3*	PGSC0003DMG400026198 [d]		chr07:47419377..47419678
Beta-amylase,PCT-BMYI	*BMY-8/1*	PGSC0003DMG402020509		chr08:1075900..1079370
Beta-amylase, plastidic, PCT-BMYI	*BMY-8/2*	PGSC0003DMG400001855	AF393847	chr08:50592100..50595690
Beta-amylase, BMYI	*BMY-8/3*	PGSC0003DMG400012129		chr08:56381800..56388400
Beta-amylase	*BMY-9*	PGSC0003DMG400001549	BD247483	chr09:57420530..57423450
Debranching enzyme, isoamylase isoform 3	*DBE-6/1*	PGSC0003DMG402007274	BD080224	chr06:368200..374700
Debranching enzyme, isoamylase isoform 3	*DBE-6/2*	PGSC0003DMG401007274	BD080224	chr06:375900..383000
Debranching enzyme, isoamylase isoform 1	*DBE-7*	PGSC0003DMG400020699	BD080223, A93395	chr07:7890210..7894280
Debranching enzyme, isoamylase isoform 2	*DBE-9*	PGSC0003DMG400000954	AY132997, BD080222	chr09:46684830..46688580

Debranching enzyme, isoamylase isoform 1	*DBE-10*	PGSC0003DMG400030253		chr10:38081140..38083210
Debranching enzyme	*DBE-11*	Not annotated	A52190	chr11:3945240..3949550
Disproportionating enzyme, 4-alpha-glucanotransferase	*DPE-2*	Not annotated	AY510449	chr02:5166700..5174600
Disproportionating enzyme, 4-alpha glucanotransferase, plastidic	*DPE-4*	PGSC0003DMG400016589	X68664	chr04:53993900..54001200
Fructokinase, StFrk3	*FRK-2*	PGSC0003DMG400030653	JX839755	chr02:41888630..41893510
Fructokinase, StFrk1	*FRK-3*	PGSC0003DMG400024246	U64817	chr03:41525810..41530530
Fructokinase	*FRK-5*	PGSC0003DMG400028311		chr05:687340..692010
Fructokinase	*FRK-6/1*	PGSC0003DMG400027017		chr06:53746100..53749420
Fructokinase, StFrk2	*FRK-6/2*	PGSC0003DMG400026916	JX576230, Z12823, U64818	chr06:53749700..53752940
Fructokinase	*FRK-9*	PGSC0003DMG400020361	NM_001288518	chr09:707190..710110
Alpha-glucan water dikinase	*GWD*	PGSC0003DMG400007677	Y09533	chr05:9823400..9839000
Glucose-6-phosphate/phosphate translocator, plastidic	*G6PPT-0*	PGSC0003DMG400005602		chr00:38861730..38863900 (DMB0000017769)
Glucose-6-phosphate/phosphate translocator, plastid	*G6PPT-1*	PGSC0003DMG400044320		chr01:67459560..67460820
Glucose-6-phosphate/phosphate translocator, plastid	*G6PPT-4*	PGSC0003DMG402012710		chr04:10972680..10976630
Glucose-6-phosphate/phosphate translocator, plastid	*G6PPT-5*	PGSC0003DMG400005269	AY163867, AF020816	chr05:42300210..42303690
Glucose-6-phosphate/phosphate translocator	*G6PPT-7/1*	PGSC0003DMG400025495		chr07:47074660..47077700
Glucose-6-phosphate/phosphate translocator, plastidic	*G6PPT-7/2*	PGSC0003DMG400001041		chr07:54548020..54553010
Hexokinase	*HXK-2*	PGSC0003DMG400030624		chr02:42162900..42168200
Hexokinase	*HXK-3*	PGSC0003DMG400002525	X94302	chr03:60521600..60527100
Hexokinase, plastidic	*HXK-4*	PGSC0003DMG400009861	NM_001288386, AF118134	chr04:70783660..70787820
Hexokinase	*HXK-6*	PGSC0003DMG400016521	NM_001287912	chr06:49126980..49131660
Hexokinase	*HXK-11*	PGSC0003DMG400013187		chr11:36684900..36690400
Hexokinase	*HXK-12*	PGSC0003DMG400000295	DQ056861	chr12:4039600..4049300
Hexose transporter	*HT-1*	PGSC0003DMG400045929		chr01:63002770..63005240
Hexose transporter	*HT-2/1*	PGSC0003DMG400022402	AJ132223, NM_001247920	chr02:34969850..34973440

Hexose transporter, plastidic	*HT-2/2*	PGSC0003DMG400026402	AF215853	chr02:41402000..41411600
Hexose transporter	*HT-4/1*	PGSC0003DMG400025054 [c]		chr04:61467750..61469290
Hexose transporter	*HT-4/2*	PGSC0003DMG402006401		chr04:63373150..63377240
Hexose transporter	*HT-4/3*	PGSC0003DMG400009994		chr04:71865900..71871700
Hexose transporter	*HT-7*	PGSC0003DMG402018758	AJ132225, NM_001247926	chr07:2681670..2686150
Hexose transporter	*HT-9*	PGSC0003DMG400031832	AJ132224, NM_001247856	chr09:53280800..53287100
Invertase, beta-fructofuranosidase, neutral	*INV-n-1/1*	PGSC0003DMG400013088		chr01:37067200..37072500
Invertase, beta-fructofuranosidase	*INV-1/2*	PGSC0003DMG400002756		chr01:78430100..78436100
Invertase, beta-fructofuranosidase	*INV-1/3*	PGSC0003DMG400001596		chr01:87160540..87164480
Invertase, beta-fructofuranosidase, soluble acid	*INV_Pain-1*	PGSC0003DMG400013856	X70368, NM_001288064	chr03:39255060..39259540
Invertase, beta-fructofuranosidase, cell wall	*INV-cw-3*	PGSC0003DMG400002583		chr03:61076900..61079820
Invertase, beta-fructofuranosidase	*INV-4*	PGSC0003DMG400009936	JX576233	chr04:70804760..70809220
Invertase, beta-fructofuranosidase, neutral /alkaline	*INV-na-6/1*	PGSC0003DMG400026107		chr06:46660300..46665800
Invertase, beta-fructofuranosidase, cell wall	*INV-6/2*	PGSC0003DMG400033142		chr06:47626000..47633600
Invertase, beta-fructofuranosidase, cell wall	*INV-cw-8/1*	PGSC0003DMG400027925		chr08:27921320..27925690
Invertase, beta-fructofuranosidase	*INV-8/2*	PGSC0003DMG400004790	NM_001247140	chr08:52701630..52705710
Invertase, beta-fructofuranosidase, cell wall	*InvGE*	PGSC0003DMG400008942	AJ133765, NM_001287894	chr09:2469540..2471550
Invertase, beta-fructofuranosidase, cell wall	*InvGF*	PGSC0003DMG400008943	AJ133765, NM_001288126	chr09:2474700..2477140
Invertase, beta-fructofuranosidase, cell wall	*INV-cw-10/1*	PGSC0003DMG400046915 [c]		chr10:15877770..15881930
Invertase, beta-fructofuranosidase	*INV-10/2*	PGSC0003DMG400008388		chr10:53235940..53238050
Invertase, beta-fructofuranosidase	*INV-10/3*	PGSC0003DMG400011037		chr10:53706680..53710620
Invertase, beta-fructofuranosidase, cell wall	*InvCD111*	PGSC0003DMG401028252	X95820, NM_001287886	chr10:55840810..55843010
Invertase, beta-fructofuranosidase, cell wall	*InvCD141*	PGSC0003DMG402028252	X95821, NM_001288398	chr10:55851950..55856800
Invertase, beta-fructofuranosidase	*INV-11/1*	PGSC0003DMG400019494		chr11:5046800..5053000
Invertase, beta-fructofuranosidase, neutral /alkaline	*INV-na-11/2*	PGSC0003DMG400009257		chr11:13676200..13683400
Invertase, beta-fructofuranosidase, neutral	*INV-n-11/3*	PGSC0003DMG400026530		chr11:39907600..39913800
Invertase/pectin methylesterase inhibitor family protein	*InvInh-3/1*	PGSC0003DMG400045232 [c]		chr03:39515876..39516269

Invertase inhibitor, Kunitz-type	*KT-InvInh-3/2*	PGSC0003DMG400010146	AF459077, AY594178, KC184127	chr03:49448370..49449150
Invertase inhibitor	*InvInh-5*	PGSC0003DMG400039425 [c]		chr05:42860389..42860866
Invertase inhibitor	*InvInh-8*	PGSC0003DMG400001844		chr08:50229740..50233130
Invertase/pectin methylesterase inhibitor family protein	*InvInh-10/1*	PGSC0003DMG400001276 [c]		chr10:22401030..22401750
Invertase inhibitor	*InvInh-10/2*	PGSC0003DMG400037656 [c]		chr10:50545200..50545720
Invertase inhibitor	*InvInh-10/3*	PGSC0003DMG400040471 [c]		chr10:51072890..51073410
Invertase inhibitor	*InvInh-10/4*	PGSC0003DMG400037039 [c]		chr10:59551460..59551980
Invertase inhibitor	*InvInh-11*	PGSC0003DMG400038811 [c]		chr11:7433850..7434380
Invertase inhibitor	*InvInh-12/1*	PGSC0003DMG400004616	KC007445, GU980595	chr12:59956850..59957600
Invertase inhibitor	*InvInh-12/2*	PGSC0003DMG400004617	NM_001247862, AY864820	chr12:59963540..59965500
Invertase inhibitor	*InvInh-12/3*	PGSC0003DMG400004618	GU980592	chr12:59967330..59968500
Maltose transporter	*MT*	PGSC0003DMG400024812		chr04:58261800..58269200
Glucose-6-phosphate isomerase	*PGI-0*	PGSC0003DMG400009848		chr00:32605770..32609090 (PGSC0003DMB000001156)
Glucose-6-phosphate isomerase	*PGI-1*	PGSC0003DMG400030128		chr01:20860570..20862590
Glucose-6-phosphate isomerase	*PGI-12*	PGSC0003DMG400015341 [d]	DQ451687	chr12:147900..160900
Phosphoglucomutase, plastidic	*PGM-0*	PGSC0003DMG400001912		chr00:12338800..12351200 (PGSC0003DMB000000431)
Phosphoglucomutase, plastidic, PGM1	*PGM-3*	Not annotated	AJ240053	chr03:41493531..41510113
Phosphoglucomutase, cytosolic	*PGM-4*	Not annotated	AJ240054	chr04:35685500..35706600
Phosphoglucomutase, plastidic	*PGM-8*	PGSC0003DMG400024224		chr08:50101900..50108200
Alpha-glucan phosphorylase, L-type, plastidic, Stp23	*PHO1a*	PGSC0003DMG400033858+ PGSC0003DMG400003495+ PGSC0003DMG400007782+ PGSC0003DMG400002479	D00520, X52385	chr03 (Chen et al. 2001), not on physical map
Alpha-glucan phosphorylase, L-type, plastidic, StpL	*PHO1b*	PGSC0003DMG400028382	X73684, BD056944	chr05:346700..352900

Alpha-glucan phosphorylase, H-type, cytosolic, StpH	*PHO2*	Not annotated	M69038, NM_001288189, L13771	chr09 (Chen et al. 2001), not on physical map (PGSC0003DMB000000644)
Phosphoglucan water dikinase	*PWD*	PGSC0003DMG400016613	AY747068	chr09:60560500..60572400
Starch branching enzyme I	*SBE I*	PGSC0003DMG400009981	X08786	chr04:71587800..71595800
Starch branching enzyme III	*SBE III*	PGSC0003DMG400022307		chr07:55034530..55039270
Starch branching enzyme II	*SBE II*	Not annotated	AJ000004	chr09:3737100..3755900
Phosphoglucan phosphatase (At3g52180)	*SEX4*	PGSC0003DMG400015246		chr03:50875700..50885600
Sucrose phosphate synthase	*SPS-7*	PGSC0003DMG400027936	X73477	chr07:3888900..3897800
Sucrose phosphate synthase	*SPS-8*	PGSC0003DMG400029892	JQ086765	chr08:20206590..20210220
Sucrose phosphate synthase isoform B	*SPS-9*	PGSC0003DMG400026428	JQ086766	chr09:59231200..59240200
Starch synthase IV	*SS IV*	PGSC0003DMG400008322	GQ221264	chr02:30142700..30152300
Starch synthase, soluble, plastidic	*Sss III*	PGSC0003DMG400016481	NM_001287873	chr02:36377150..36379320
Starch synthase V	*SS V*	PGSC0003DMG400030619	EU661369	chr02:42100900..42107600
Granule bound starch synthase II, plastidic, SSII	*GbssII*	PGSC0003DMG400001328	NM_001288048	chr02:46189600..46196700
Starch synthase, soluble	*SssI*	PGSC0003DMG402018552 [b]	NM_001288145	chr03:45887500..45896600
Starch synthase VI	*SS VI-7/1*	PGSC0003DMG401013540		chr07:40921600..40929800
Starch synthase VI	*SS VI-7/2*	PGSC0003DMG402013540		chr07:40932600..40939900
Granule bound starch synthase I, plastidic, waxy	*GbssI*	PGSC0003DMG400012111	X58453	chr08:56782190..56785790
Sucrose synthase	*SUS-2*	PGSC0003DMG400016730		chr02:36937430..36941850
Sucrose synthase	*SUS-3*	PGSC0003DMG400031046		chr03:48949000..48950010
Sucrose synthase	*SUS-7/1*	PGSC0003DMG400013547		chr07:40610340..40613980
Sucrose synthase, Sus3	*SUS-7/2*	PGSC0003DMG400013546	U24088, AY205084	chr07:40638500..40644900
Sucrose synthase	*SUS-9*	PGSC0003DMG400006672	AY205302	chr09:61464900..61472300
Sucrose synthase, Sus4	*SUS-12*	PGSC0003DMG400002895	U24087, DQ793135	chr12:3085100..3091500
Sucrose transporter	*Sut2*	PGSC0003DMG400025610	NM_001288509, AY291289	chr05:5692340..5694730
Sucrose transporter	*Sut1*	PGSC0003DMG400009213	X69165	chr11:9052430..9057350

Sucrose transporter	*Sut4*	Not annotated	NM_001288141, AF237780	chr04:65861800..65873100
UDP-glucose pyrophosphorylase	*UGPase-1*	PGSC0003DMG401031123		chr01:67210700..67220100
UDP-glucose pyrophosphorylase	*UGPase-11*	PGSC0003DMG401013333	D00667, NM_001288019	chr11:808300..814800
		Total 123	Total 66	

[a] Loci without established names from the literature are distinguished based on chromosomal location, loci tested for association are highlighted green.

[b] Not annotated in genome browser v4.03

[c] Very low or no expression, pseudogene?

[d] Only small gene fragment, incomplete annotation
