# Supplementary material for: SNPs in Genes Functional in Starch-Sugar Interconversion Associate with Natural Variation of Tuber Starch and Sugar Content of Potato (Solanum tuberosum L.)
Source: G3 (Bethesda). 2014 Jul 31;4(10):1797–811. doi: 10.1534/g3.114.012377 (PMC4199688; doi:10.1534/g3.114.012377)
Supplement: Supporting Information [file supp_g3.114.012377_TableS2.pdf]

Table S2 Correspondence between presence (1) or absence (0) of the Stp23-8b SSCP marker and PHO1a cDNA SNPs in 34 standard varieties of the CHIPS-ALL population (Li *et al*. 2008).

Variety	Stp23-8b	$C_{22}T$	$G_{322}A$	G_{824}	A_{2776}
Leyla	0	CCCC	GGGG	0	0
Marabel	0	CCCC	GGGG	0	0
Solara	0	CCCC	GGGG	0	0
Vitara	0	CCCC	GGGG	0	0
Milva	0	CCCC	GGGG	0	0
Ponto	1	TCCC	AGGG	1	1
Tomensa	1	TCCC	AGGG	1	1
Marlen	1	TCCC	AGGG	1	1
Eldena	1	TCCC	AGGG	1	1
Theresa	1	TCCC	AGGG	1	1
Goldika	1	TCCC	AGGG	1	1
Saturna	1	TTCC	AAGG	1	1
Karlena	0	CCCC	GGGG	0	0
Kolibri	0	CCCC	GGGG	0	0
Fasan	0	CCCC	GGGG	0	0
Terra	0	TCCC	AGGG	1	1
Solist	0	CCCC	GGGG	0	0
Melina	1	TCCC	AGGG	1	1
Molli	0	CCCC	GGGG	0	0
Likaria	0	CCCC	GGGG	0	0
Valisa	0	CCCC	GGGG	0	0
Apart	0	CCCC	GGGG	0	0
Artis	0	CCCC	GGGG	0	0
Aula	0	CCCC	GGGG	0	0
Christa	0	CCCC	GGGG	0	0
Diana	1	TCCC	AGGG	1	1
Novara	0	CCCC	GGGG	0	0
Ilona	0	CCCC	GGGG	0	0
Orlando	0	CCCC	GGGG	0	0
Panda	1	TCCC	AGGG	1	1
Satina	0	CCCC	GGGG	0	0
Sempra	0	CCCC	GGGG	0	0
Sirius	1	TCCC	AGGG	1	1
Velox	0	CCCC	GGGG	0	0
